# Brain‐derived plasma p‐tau217 shows enhanced dynamic range for Alzheimer's disease neuropathological change

**DOI:** 10.1002/alz.71773

**Published:** 2026-09-01

**Authors:** Marisa N. Denkinger, Wagner S. Brum, Antoine Leuzy, Alpana Singh, Taina M. Marques, Valentina Ghisays, Hillary Protas, Yi Su, Ali Atri, Thomas G. Beach, Geidy E. Serrano, Kinal Bhatt, Corey M. Carlson, Katharine Hoffmann, Eric M. Reiman, Nicholas J. Ashton

**Affiliations:** ^1^ Banner Sun Health Research Institute Sun City Arizona USA; ^2^ ZRO Imaging Santiago de Compostela Spain; ^3^ Banner Alzheimer's Institute Phoenix Arizona USA; ^4^ Beckman Coulter Inc. Chaska Minnesota USA

**Keywords:** Alzheimer's disease, amyloid, blood biomarkers, brain‐derived tau, diagnosis, neuropathology, phosphorylated tau, plasma biomarkers, p‐tau217

## Abstract

**INTRODUCTION:**

Plasma phosphorylated tau (p‐tau), particularly p‐tau217, is a highly specific biomarker of Alzheimer's disease (AD) pathology. However, plasma p‐tau217 can be elevated in rare non‐AD conditions. Brain‐derived (BD) p‐tau217 may reduce these off‐target effects, but its performance against neuropathology has not been evaluated.

**METHODS:**

We compared p‐tau217, BD p‐tau217, their amyloid beta 42 (Aβ42) ratios, BD p‐tau217/p‐tau217, and BD p‐tau217/BD tau in *end‐of‐life* plasma from 288 neuropathologically characterized participants using a fully automated immunoassay. Biomarkers were assessed against National Institute on Aging–Alzheimer's Association (NIA‐AA) classification, Thal phase, Braak stage, cognitive decline, and tau‐PET (positron emission tomography).

**RESULTS:**

All markers tracked neuropathological severity, with BD p‐tau217 having larger fold‐changes than p‐tau217 but BD p‐tau217/Aβ42 enhancing this further. BD p‐tau217/BD tau achieved the highest area under the curve (AUC) for distinguishing Intermediate/High from Not/Low AD neuropathological change (ADNC) (0.89 vs 0.82 for p‐tau217). Although BD p‐tau217/p‐tau217 showed smaller fold‐changes, it had the strongest association with continuous tangle burden in AD (*R^2^ =* 0.68) and best predicted Clinical Dementia Rating Sum of Boxes (CDR‐SB decline) (*R^2^ =* 0.26).

**DISCUSSION:**

BD p‐tau217 and BD‐based ratios enhance dynamic range and prognostic performance while maintaining diagnostic accuracy, supporting further clinical evaluation.

## Highlights

- We validated brain‐derived (BD) phosphorylated tau 217 (p‐tau217) in 288 neuropathologically confirmed cases using a fully automated platform.
- BD p‐tau217/amyloid beta 42 (Aβ42) showed an 8.3‐fold increase in High Alzheimer's disease (AD) versus Not AD.
- BD p‐tau217 biomarkers more strongly tracked quantitative plaque and tangle burden.
- BD variants improved the prediction of longitudinal cognitive decline.
- A fully automated platform supports scalable clinical implementation.

## 1 BACKGROUND

Alzheimer's disease (AD) is the leading cause of dementia globally, characterized biologically by the accumulation of amyloid beta (Aβ) plaques and tau neurofibrillary tangles.^1^ Historically, a definitive AD diagnosis relied on postmortem neuropathological assessment, but the development of highly accurate cerebrospinal fluid (CSF) and positron emission tomography (PET) biomarkers has transformed this paradigm, shifting the disease definition to a clinico‐biological construct.^1^, ^2^, ^3^ Despite their high diagnostic accuracy for clinically manifest AD pathology, PET and CSF procedures are associated with high cost, specialized infrastructure requirements, and invasiveness, which limit widespread implementation in clinical settings or large‐scale screening and trial recruitment efforts. Recent developments in blood‐based biomarkers (BBMs) have revolutionized the field by providing minimally invasive and highly accurate tools to identify AD pathological changes.^4^, ^5^ Phosphorylated tau (p‐tau) biomarkers, especially p‐tau217, demonstrate superior diagnostic accuracy and disease specificity over other BBMs, consistently exhibiting high accuracy in detecting abnormal Aβ and tau pathologies.^6^, ^7^, ^8^, ^9^

In CSF, total‐tau, a non‐phospho‐specific tau biomarker, is a well‐established marker of neurodegeneration, but its translation to blood has faced challenges.^2^, ^10^ Conventional plasma total tau (t‐tau) assays often perform poorly because the tau protein, encoded by the microtubule‐associated protein tau (*MAPT*) gene, exists in multiple isoforms, including a high molecular weight variant (“big tau”) that is abundant in peripheral tissues.^10^, ^11^, ^12^ This peripheral interference has complicated interpretation of standard t‐tau measurements, leading to the development of a brain‐derived (BD) assay for non‐phosphorylated tau, designed using an antibody that selectively targets the exon 4–5 junction, enabling specific quantification of central nervous system (CNS)–derived tau isoforms and minimizing peripheral interference.^13^

The research value of BD‐tau remains under investigation, and comparable “brain‐derived” adaptations have been less explored for p‐tau biomarkers, leaving open the question of whether BD p‐tau variants could further enhance diagnostic performance and disease specificity. Recent evidence has suggested that peripheral neurodegenerative processes, most prominently amyotrophic lateral sclerosis (ALS), are associated with modest but significant elevations in plasma p‐tau.^14^, ^15^, ^16^, ^17^ A peripheral contribution to this increased p‐tau signal in ALS, for instance, is supported by findings indicating the presence of p‐tau181 and p‐tau217 immunoreactivity in muscle tissue, with increased reactivity in atrophic muscle fibers, suggesting denervated muscle or degenerating peripheral axons as potential sources of circulating p‐tau species.^15^ Immunoassay design influences the susceptibility to this confounding effect. A study comparing different assays found that p‐tau assays preferentially targeting low molecular weight tau (CNS‐type) showed substantially smaller increases in ALS versus controls, compared with assays that also detect high molecular weight (peripheral) tau.^16^ This raises AD‐specificity concerns that may be of higher importance as plasma p‐tau implementation moves toward screening in larger unselected populations, primary care use, or even differential diagnosis in settings where peripheral neuropathy or neuromuscular diseases may be more common, suggesting that a “CNS‐specific” or “BD p‐tau” assay could be fundamentally important for clinical implementation.^18^

To address this gap, we evaluated a novel fully automated assay on a clinical laboratory platform for p‐tau217, BD p‐tau217 and Aβ 1‐42 (Aβ42) to compare the performance between these two p‐tau variants and their ratios to Aβ42. The inclusion of Aβ42 was motivated by the U.S. Food and Drug Administration (FDA) approval of the plasma p‐tau217/Aβ42 ratio, which has been used increasingly in AD research.^19^ Leveraging a large, well‐characterized clinicopathological cohort of nearly 300 participants from the Banner Sun Health Institute's Brain and Body Donation Program (BBDP), we examined the associations of p‐tau217, BD p‐tau217, and their respective Aβ42 ratios, as well as the BD p‐tau217/p‐tau217 and BD p‐tau217/BD tau ratios with AD neuropathological change (ADNC). We aimed to determine whether a BD p‐tau217 assay provides value beyond a non–BD‐specific plasma p‐tau217 variant in a postmortem research cohort.

## 2 METHODS

### 2.1 Participants

We included participants from the Arizona Study of Aging and Neurodegenerative Disorders (AZSAND) and the Brain and Body Donation Program (BBDP), who underwent annual medical, neurological, and movement examinations, as well as cognitive assessments. Participants could additionally opt into genetic testing, fluid biomarker studies, and neuroimaging protocols. Participants included in this study provided longitudinal plasma samples during life and later underwent postmortem neuropathological assessment.^20^ For analyses focused on neuropathological outcomes, we used biomarker measurements from the plasma samples collected closest to autopsy. For analyses of the predictive value of BBMs for subsequent cognitive decline, we used biomarker measurements from the first plasma sample collected during study participation. A subset of participants also underwent tau‐PET imaging before death; for these analyses, we used plasma samples collected closest to the tau‐PET scan when available, rather than those collected closest to death.

**RESEARCH IN CONTEXT**

1. **Systematic review**: We reviewed the prior literature using PubMed and major conference proceedings focusing on plasma phosphorylated tau 217 (p‐tau217), brain‐derived (BD) tau assays, and clinicopathological validation studies. Although plasma p‐tau217 is well established as a marker of Alzheimer's disease (AD), limited data exist comparing BD p‐tau217 to conventional assays against neuropathologic gold standards or evaluating dynamic range and prognostic performance.
2. **Interpretation**: In a neuropathologically confirmed cohort, BD‐based measures demonstrated substantially expanded dynamic range and stronger associations with quantitative plaque and tangle burden, while maintaining excellent diagnostic accuracy. BD variants also improved longitudinal prediction of cognitive decline, supporting enhanced disease specificity.
3. **Future directions**: Future studies should validate BD p‐tau217 in intended‐use clinical populations, assess performance in non‐AD comorbidities (e.g., neuromuscular disease), and determine whether improved dynamic range translates to fewer equivocal classifications and better therapeutic monitoring.

### 2.2 Neuropathological assessment

ADNC levels were determined according to the National Institute on Aging–Alzheimer's Association (NIA‐AA) criteria, which integrates Thal amyloid phase, Braak neurofibrillary tangle stage, and the Consortium to Establish a Registry for Alzheimer's Disease (CERAD) neuritic plaque score into a composite “ABC” classification.^21^ Participants were assigned to one of four levels of ADNC (Not AD, Low, Intermediate, or High ADNC) based on the likelihood of AD indicated by the combined burden of amyloid plaques and neurofibrillary tangles. These classifications were used for postmortem pathological stratification and research analyses. In addition to categorical ADNC classification, each participant had detailed semi‐quantitative regional measures of pathology. Plaque total score and tangle total scores were defined as the sum of senile plaque and neurofibrillary tangle density scores (none, sparse, moderate, and frequent; 0–3 for statistical purposes) in the frontal, temporal, parietal, hippocampal, and entorhinal cortices (total possible score of 15). For sensitivity analyses focused on evaluating the effect of co‐pathologies over BBM signal, we included: cerebral amyloid angiopathy (CAA), Lewy bodies, trans‐activation response (TAR) DNA‐binding protein 43 (TDP‐43), and vascular pathology. Co‐pathology status was determined by the presence or absence of each pathology in the final pathological diagnosis report.

### 2.3 Cognitive and clinical assessment

Cognitive and clinical assessments were performed annually using a comprehensive, standardized battery spanning neurological, neuropsychological, and movement disorder domains. The evaluation included general medical and medication history, substance use (tobacco and alcohol), and a general neurological examination. Global cognition and function were assessed using the Mini‐Mental State Examination (MMSE), Clinical Dementia Rating (CDR) scale, Montreal Cognitive Assessment, Mattis Dementia Scale, Geriatric Depression Scale, and Functional Assessment Staging Test. The neuropsychological battery included the Rey Auditory Verbal Learning Test, Stroop test, Trail Making Test Parts A and B, Controlled Oral Word Association Test, Judgment of Orientation, clock drawing, and Digit Span (forward and backward). Movement and related non‐motor features were assessed using the Unified Parkinson's Disease Rating Scale, Hoehn and Yahr scale, timed tapping test, Purdue Pegboard Test, tremor rating, restless legs syndrome score, Mayo Clinic Sleep Questionnaire, the Scales for Outcomes in Parkinson's Disease‐Autonomic (SCOPA‐AUT) questionnaire, and the University of Pennsylvania Smell Identification Test. For the present study, analyses focused on the CDR Sum of Boxes (CDR‐SB) and MMSE as primary measures of cognitive performance.

### 2.4 Plasma sampling

Blood samples were collected in the morning from non‐fasting participants using K_2_EDTA tubes (Vacutainer, BD Diagnostics). Samples were centrifuged at 1500 × *g* for 15 min at 4°C. After centrifugation, plasma from all tubes was combined into a single 50 mL polypropylene tube, gently mixed, and aliquoted in 0.5 mL portions into 1.5 mL polypropylene tubes. Aliquots were stored at −80°C within ≈30–60 min of collection. No additional pre‐analytical harmonization procedures were applied beyond standard handling protocols.

### 2.5 Plasma biomarker quantification

Plasma biomarkers were measured using fully automated immunoassays intended for research use only (RUO) on the DxI 9000 Access Immunoassay Analyzer (Beckman Coulter). The commercially available Access p‐Tau217 (RUO), Access β‐Amyloid 1‐42 (RUO), and Access BD‐tau (RUO) assays were used to quantitate p‐tau217, Aβ42, and BD tau (non‐phosphorylated tau), respectively. BD p‐tau217 was measured using a prototype assay in development at the time of the study. K_2_EDTA plasma samples were thawed, vortexed for 10 s, and centrifuged at 2000 × *g* for 5 min at 4°C. Single measurements were obtained for each sample for each assay blinded to clinical, imaging, and neuropathological data. Biomarker results were not used for clinical diagnosis or decision‐making. No samples had concentrations below the limit of detection for any of the biomarkers, and imprecision ranged from 1.9% to 9.0% for all assays (Table S1). Of *n =* 288 eligible donors, the availability of BBM measurements was as follows: p‐tau217, *n =* 281; p‐tau217/Aβ42, *n =* 272; BD p‐tau217, *n =* 284; BD p‐tau217/Aβ42, *n =* 274; BD p‐tau217/p‐tau217, *n =* 281; and BD p‐tau217/BD tau, *n =* 279.

### 2.6 Tau‐PET imaging

[^18^F]‐Flortaucipir (FTP) scans were acquired on one of the two scanners (General Electric MIDR PET/CT and General Electric Discovery 710 PET/CT [computed tomography]). FTP scans were acquired with a 10 mCi (± 10%) bolus injection, and a 30‐min dynamic emission scan starting at 75 min after injection in six frames. All T1 magnetic resonance imaging (MRI) studies for a subject were co‐registered with each other and rigidly aligned with the Montreal Neurological Institute (MNI) template. FreeSurfer v7.1 and Computational Anatomy Toolbox (CAT12) (Jena University Hospital, Germany) were used to process the co‐registered T1 images to define spatial normalization deformation fields. The FTP PET scans were processed through the PET unified pipeline (https://github.com/ysu001/PUP).^22^, ^23^ In this pipeline, the PET images undergo several steps including smoothing to a common resolution, between‐frame motion correction, frame summation, PET‐to‐MRI co‐registration, and standardized uptake value ratio (SUVR) estimation in FreeSurfer‐defined regions of interest (ROIs). All FTP regional SUVRs went through partial volume correction using regional spread function.^22^ FTP PET images were subsequently transformed to the MNI template space with the deformation field from their matching T1 image. For this study, we evaluated the middle temporal, inferior temporal, and a weighted average of all cortical ROIs.^24^ The cerebellar cortex was used as the reference region.^25^, ^26^

### 2.7 Statistical analysis

Continuous and categorical variables were summarized as medians with interquartile ranges (IQRs) and counts with percentages (%), respectively. For main analyses involving neuropathological variables, the plasma measurement closest to death was used. For analyses within the subset with tau‐PET, the measurement closest to the scan date was used instead.

Group comparisons of plasma biomarker levels across neuropathological categories (NIA‐AA ADNC, Thal phase, and Braak stage) used linear models adjusted for age at death, sex, and the plasma‐autopsy interval. Adjusted pairwise group differences were estimated from model‐based marginal means with Sidak correction.^27^ Due to their multiplicative nature and to enable adjustment for age at death, sex, and interval between plasma sampling and autopsy, fold‐changes (FCs) were estimated from log‐linear models. Coefficients were exponentiated to obtain adjusted mean fold‐changes and 95% confidence intervals (CIs) relative to prespecified reference categories (Not AD, Thal A0, and Braak I–II, respectively). Between‐biomarker differences in fold‐change were evaluated on the log scale using nonparametric bootstrap resampling (*n =* 5000), with percentile 95% CIs and two‐sided bootstrap *p*‐values, adjusted for false discovery rate (FDR) using the Benjamini–Hochberg procedure.^28^

Associations between plasma biomarkers and continuous plaque and tangle scores were examined using regression models with the neuropathology score as the dependent variable, adjusted for age at death, sex, and the plasma‐autopsy interval. For each biomarker–outcome combination, we compared an adjusted linear model with an adjusted generalized additive model (GAM) incorporating a penalized regression spline (basis dimension *k = *5). The GAM was selected when it improved fit by at least 2 Akaike Information Criterion (AIC) points relative to the linear model (ΔAIC ≤−2). Explained variance (R^2^) was computed from observed and fitted values; the biomarker contribution was summarized as ΔR^2^ relative to a covariate‐only base model. Analyses were performed in the full analytic cohort and repeated after stratification by neuropathological AD status (Non‐AD: Not AD/Low ADNC; AD: Intermediate/High ADNC). Pairwise between‐biomarker differences in GAM R^2^ were quantified using paired bootstrap resampling (*n =* 5000), with all six models refit on the same resampled dataset in each replicate; bootstrap *p*‐values were FDR‐adjusted within each outcome‐population stratum. As a sensitivity analysis, the same framework was applied to adjusted linear models, with between‐biomarker differences in standardized β coefficients and linear R^2^ evaluated using the same paired bootstrap design.^29^

Discriminative accuracy for dichotomized neuropathological outcomes was evaluated using receiver operating characteristic (ROC) curves and areas under the ROC curve (AUCs) with 95% CIs. Paired DeLong tests compared AUCs across biomarkers, with FDR adjustment applied across pairwise contrasts. Outcomes involved the following neuropathological contrasts: NIA‐AA, Not AD/Low ADNC versus Intermediate/High ADNC, Not AD versus High ADNC; Thal phase, A0–A1 versus A2–A3, A0 versus A3; Braak stages, I–II versus III–VI, I–II versus V–VI.

For longitudinal analyses, linear mixed‐effects models with participant‐level random intercepts and correlated random slopes for time were used to relate baseline plasma biomarker levels to subsequent cognitive trajectories. Time was defined as years since each participant's first blood draw and was capped at 8 years to reduce instability in estimates arising from sparse observations at longer follow‐up durations. Baseline biomarker values were defined as each participant's first non‐missing measurement and were z‐standardized before analysis. Longitudinal mixed‐effects models were fit separately for CDR‐SB and MMSE, with cognition modeled as a function of time, baseline biomarker level, and their interaction. Models adjusted for age at blood draw, sex, and years of education. CDR‐SB was inverted so that, for both outcomes, higher values indicated better cognition. For comparability, all biomarker models for a given outcome were fit on a common analytic sample requiring non‐missing baseline values for all six biomarkers. Between‐biomarker differences in the standardized time × biomarker interaction estimate and in marginal R^2^ were evaluated using participant‐level cluster bootstrap resampling (*n =* 5000), with FDR correction applied separately within each outcome for pairwise differences in β, pairwise ΔR^2^, and ΔR^2^ relative to a covariate‐only model.

In the tau‐PET imaging subset, associations between plasma biomarkers and regional [^1^ ^8^F]‐flortaucipir uptake were assessed using Spearman correlations. As an exploratory sensitivity analysis, associations between plasma biomarkers and non‐AD co‐pathologies (CAA, Lewy body pathology, TDP‐43 pathology, and vascular pathology) were examined using nested linear models with each log‐transformed, z‐standardized biomarker as the dependent variable and co‐pathology presence as the binary predictor. Models were fit sequentially: unadjusted; adjusted for age at death, sex, and the plasma‐autopsy interval; additionally adjusted for plaque burden; and additionally adjusted for both plaque and tangle burden. All tests were two‐sided with α = 0.05; FDR correction is noted where applied. Analyses were conducted in R version 4.5.2.

## 3 RESULTS

### 3.1 Participants

Participants (*N =* 288) were categorized into four neuropathological groups according to the NIA‐AA ADNC staging scheme: Not AD (*n =* 63; 21.9%), Low ADNC (*n =* 55; 19.1%), Intermediate ADNC (*n =* 114; 39.6%), and High ADNC (*n =* 56; 19.4%). The median age at death was 86 years (IQR, 80–92), 44.4% were female, 78 (27.1%) were apolipoprotein E (*APOE*) ε4 carriers and median years of education was 16 (IQR, 13–18) across all groups. Cognitive performance, as measured by the last available MMSE, remained similar across Not AD and Low and Intermediate ADNC categories with a median of 26–27, dropping to a median score of 13.5 in the High ADNC group. The median interval between the last blood sample and autopsy was 1.4 years (IQR, 0.7–2.6) in the main sample. Regarding co‐pathologies, evidence of CAA was present in 168 participants (58.3%), Lewy body pathology in 124 (44.3%), TDP‐43 pathology in 87 (31.9%), and cerebrovascular pathology in 131 (45.5%). For comparability, several neuropathology‐related analyses were performed in a complete‐case subset comprising 269 participants with non‐missing data for all six biomarkers evaluated in the study. Demographic, cognitive, and co‐pathology characteristics were similar in this subset and the overall cohort, with a median follow‐up of 2.5 years (IQR, 1.3–4.1) (Table S2). Overall, the cohort captured a broad spectrum of AD neuropathological severity with balanced sex distribution and an age profile typical of community‐based autopsy series (Table 1).

**TABLE 1:** Participant information.

Characteristic	Not AD *N* = 63	Low ADNC *N* = 55	Int. ADNC *N* = 114	High ADNC *N* = 56	Overall *N* = 288
Age at death (years)	86.0 (80.0–93.0)	82.0 (76.0–89.0)	87.5 (82.0–93.0)	85.0 (79.0–90.5)	86.0 (80.0–92.0)
Female	32 (50.8%)	22 (40.0%)	53 (46.5%)	21 (37.5%)	128 (44.4%)
APOEε4 carriers	3 (4.8%)	14 (25.5%)	29 (25.4%)	32 (57.1%)	78 (27.1%)
Years of education	16.0 (14.0–17.0)	16.0 (14.0–18.0)	16.0 (13.0–18.0)	14.0 (12.0–16.0)	16.0 (13.0–18.0)
Follow‐up, baseline to last assessment (years)	2.9 (1.2–4.8)	2.7 (1.8–4.8)	2.2 (1.4–3.3)	2.1 (1.1–3.2)	2.4 (1.2–4.0)
Interval between last blood draw and autopsy (years)	1.1 (0.7–2.4)	1.0 (0.7–1.8)	1.8 (0.9–2.8)	1.7 (0.7–3.0)	1.4 (0.7–2.6)
Baseline MMSE	28.0 (24.0–29.0)	27.0 (25.0–29.0)	27.0 (24.0–29.0)	20.5 (16.0–25.0)	26.0 (23.0–29.0)
Last MMSE	26.0 (23.0–28.0)	27.0 (24.0–28.0)	26.0 (22.0–28.0)	13.5 (6.0–23.0)	25.0 (20.0–28.0)
Baseline CDR‐SoB	0.5 (0.0–3.0)	1.0 (0.0–2.5)	1.0 (0.0–3.5)	9.0 (4.5–11.5)	1.0 (0.0–5.0)
Last CDR‐SoB	1.0 (0.0–4.5)	1.0 (0.0–2.5)	1.0 (0.0–5.0)	11.0 (5.0–15.0)	1.5 (0.0–7.0)
Co‐pathology, CAA	7/63 (11.1%)	29/55 (52.7%)	78/114 (68.4%)	54/56 (96.4%)	168/288 (58.3%)
Co‐pathology, Lewy body	23/63 (36.5%)	23/52 (44.2%)	48/109 (44.0%)	30/56 (53.6%)	124/280 (44.3%)
Co‐pathology, TDP‐43	15/57 (26.3%)	9/52 (17.3%)	32/112 (28.6%)	31/52 (59.6%)	87/273 (31.9%)
Co‐pathology, vascular	30/63 (47.6%)	21/55 (38.2%)	58/114 (50.9%)	22/56 (39.3%)	131/288 (45.5%)

Abbreviations: CDR‐SoB, Clinical Dementia Rating Sum of Boxes; MMSE, Mini‐Mental State Examination; TDP‐43, transactive response DNA‐binding protein 43.

Participant information is presented according to NIA‐AA ADNC stages. Continuous variables are summarised as median (interquartile range); categorical variables as n (%). Co‐pathology variables are shown as n/N (%), where N is the number of participants with available data. Follow‐up is the interval between the baseline blood sample and the last cognitive assessment. Last MMSE and CDR‐SoB are taken from the latest available longitudinal visit. ADNC = Alzheimer's disease neuropathologic change; CAA = cerebral amyloid angiopathy.

### 3.2 Group differences and fold‐changes across neuropathological stages

**FIGURE 1 alz71773-fig-0001:**
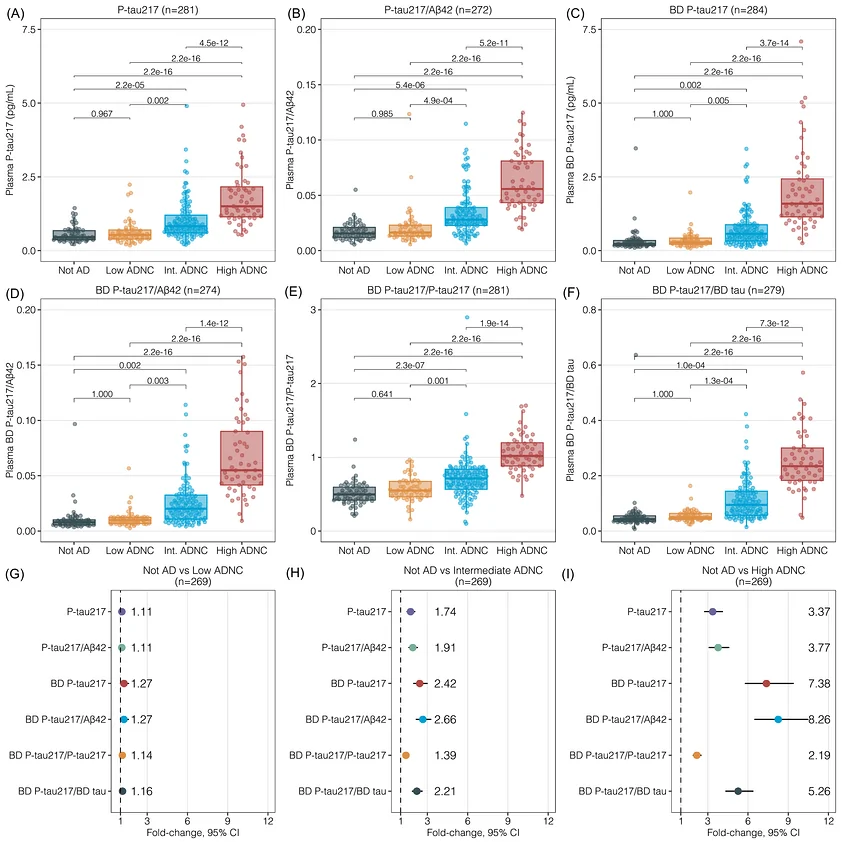
Plasma biomarker levels and adjusted fold‐changes across ADNC categories. Boxplots with individual datapoints show group‐wise distributions of p‐tau217 (A), p‐tau217/Aβ42 (B), BD p‐tau217 (C), BD p‐tau217/Aβ42 (D), BD p‐tau217/p‐tau217 (E), and BD p‐tau217/BD tau (F) across Not, Low, Intermediate, and High ADNC groups. For boxplots, each biomarker was analyzed using the maximum available analytic sample for that biomarker, restricted to participants with non‐missing NIA‐AA ADNC status and model covariates, with the effective sample size shown in each panel title. The *p*‐values are from post hoc contrasts from linear models adjusted for age at death, sex, and interval between plasma sampling and autopsy, with Sidak adjustment for multiple pairwise comparisons. Forest plots show mean fold‐changes and 95% CIs for Low (G), Intermediate (H), and High (I) ADNC groups compared with Not AD as the reference group, adjusted for age at death, sex, and interval between plasma sampling and autopsy. For comparability, fold‐changes were estimated in a common complete‐case subset with non‐missing values for all six biomarkers, with the effective sample size shown in each forest plot title. Aβ, amyloid beta; AD, Alzheimer's disease; ADNC, AD neuropathological change; BD, brain‐derived; CI, confidence interval; NIA‐AA, National Institute on Aging–Alzheimer's Association; p‐tau, phosphorylated tau.

Generally, as pathological burden increased, levels of all six plasma biomarkers (p‐tau217, p‐tau217/Aβ42, BD p‐tau217, BD p‐tau217/Aβ42, BD p‐tau217/p‐tau217, and BD p‐tau217/BD tau) demonstrated progressive, stepwise, and significant increases across pathology‐defined categories, with boxplots constructed using the maximum number of biomarker measurements available. When evaluating biomarker levels according to NIA‐AA ADNC stages (Not AD and Low, Intermediate, and High ADNC; Figure 1A–F), all biomarkers remained stable between the Not AD and Low ADNC categories. In contrast, Intermediate ADNC cases showed marked elevations in all biomarkers, with values significantly higher than the Not AD and Low ADNC groups, yet significantly lower than in the High ADNC group.

To complement group‐level differences shown by boxplots, we calculated the mean fold‐change for each biomarker relative to the respective reference group within the complete case subset with non‐missing values for all six biomarkers. For the Low ADNC group (Figure 1G), fold‐changes relative to the Not AD reference were modest for all biomarkers (range: 1.11–1.27) (Table S3). Despite the overall small magnitude, bootstrapped comparisons indicated that BD p‐tau217 showed a significantly greater fold‐change than p‐tau217 (*P_FDR_* = 0.044), as did BD p‐tau217/Aβ42 relative to p‐tau217/Aβ42 (*P_FDR_* = 0.044); the remaining pairwise comparisons did not reach statistical significance (Table S4).

For the Intermediate ADNC group (Figure 1H), fold‐changes diverged more clearly across biomarker classes (Table S4). The p‐tau217 reached a fold‐change of 1.74 (95% CI 1.47–2.06) and p‐tau217/Aβ42 a fold‐change of 1.91 (95% CI 1.62–2.25), with p‐tau217/Aβ42 significantly exceeding p‐tau217 (*P_FDR_* = 0.041). BD p‐tau217 (2.42, 95% CI 1.98–2.97) and BD p‐tau217/Aβ42 (2.66, 95% CI 2.17–3.25) both showed significantly larger fold‐changes than either non‐BD biomarker (all *P*_FDR_ < 0.001), and BD p‐tau217/Aβ42 additionally exceeded BD p‐tau217 (*P*_FDR_ = 0.041). BD p‐tau217/BD tau (2.21, 95% CI 1.88–2.60) was also significantly greater than p‐tau217 (*P*_FDR_ = 0.007). In contrast, BD p‐tau217/p‐tau217 had the smallest fold‐change in this group (1.39, 95% CI 1.25–1.56), which was significantly lower than p‐tau217 (*P*_FDR_ = 0.041) and markedly lower than both BD p‐tau217 and BD p‐tau217/Aβ42 (both *P*_FDR_ < 0.001).

For the High ADNC group (Figure 1I), differences between BD and non‐BD biomarkers were substantially amplified (Table S4). The p‐tau217 showed a fold‐change of 3.37 (95% CI 2.77–4.10) and p‐tau217/Aβ42 a fold‐change of 3.77 (95% CI 3.11–4.58), with p‐tau217/Aβ42 significantly exceeding p‐tau217 (*P*_FDR_ = 0.024). BD p‐tau217 (7.38, 95% CI 5.81–9.37) and BD p‐tau217/Aβ42 (8.26, 95% CI 6.51–10.47) were both significantly greater than their non‐BD counterparts (all *P*_FDR_ < 0.001), with BD p‐tau217/Aβ42 additionally exceeding BD p‐tau217 (*P*_FDR_ = 0.024). BD p‐tau217/BD tau showed an intermediate fold‐change (5.26, 95% CI 4.35–6.38) that was significantly greater than both non‐BD biomarkers (both *P*_FDR_ < 0.001), yet significantly lower than BD p‐tau217 and BD p‐tau217/Aβ42 (both *P*_FDR_ < 0.001). BD p‐tau217/p‐tau217 again showed the lowest fold‐change in this comparison group (2.19, 95% CI 1.92–2.50) and was the only biomarker for which fold‐change was significantly lower than all others, including both non‐BD markers (all *P*_FDR_ < 0.001).[Fig alz71773-fig-0001]

Consistent with these findings, all biomarkers also showed significant increases with advancing pathological severity but with BD variants demonstrating substantially larger fold‐changes when pathological groups were defined by Thal amyloid phases (Figure S1, Tables S5–S6) and Braak tau stages (Figure S2, Table S7–S8). When examining fold changes using the maximum available number of individuals with non‐missing outcome and blood biomarker, magnitude and order of BBM associations remained similar (Figure S3) for all outcomes and contrasts.

### 3.3 Relationship between plasma biomarkers and continuous plaque and tangle counts

**FIGURE 2:**
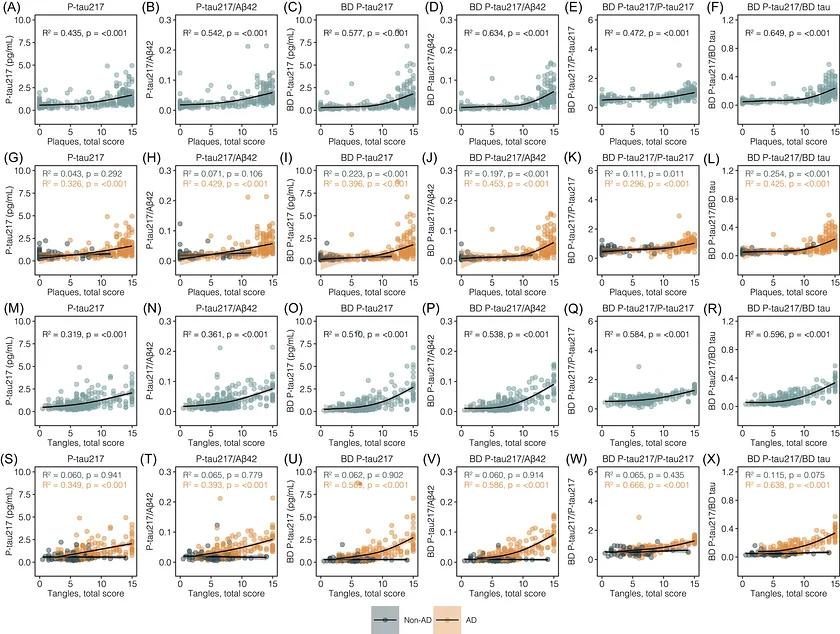
Continuous associations between plasma biomarkers and neuropathological measures of plaques and tangles. Scatterplots show associations of plasma biomarkers with semiquantitative plaque and tangle measures at autopsy. Panels include p‐tau217, p‐tau217/Aβ42, BD p‐tau217, BD p‐tau217/Aβ42, BD p‐tau217/p‐tau217, and BD p‐tau217/BD tau. Panels A–F represent biomarker–plaque associations for the unstratified whole population; G–L for the pathology‐stratified Non‐AD (Not AD or Low ADNC) and AD (Intermediate and High ADNC) populations. Panels M–R represent biomarker‐tangle associations for the unstratified whole population; S–X for the pathology‐stratified Non‐AD and AD populations. Model annotations show R^2^ and *p*‐values for the smooth term from pathology‐biomarker models adjusted for age at death, sex, and interval between plasma sampling and autopsy. Curves represent generalized additive model smooths. Analyses were performed in the complete‐case subset with non‐missing values for all six blood biomarkers (*n =* 269). AD, Alzheimer's disease; ADNC, AD neuropathological change; BD, brain‐derived; p‐tau, phosphorylated tau.

We examined continuous associations between plasma biomarkers and continuous measures of amyloid plaque and tau tangle pathology in the unstratified cohort, followed by stratified analyses according to pathological AD status (Non‐AD: Not AD/Low ADNC; AD: Intermediate/High ADNC) (Figure 2). Associations were characterized using regression models adjusted for age at death, sex, and the interval between plasma sampling and autopsy, with GAMs selected over linear models when they improved fit by at least 2 AIC points. GAMs were preferred for virtually all biomarker‐outcome combinations (Tables S9–S10) and therefore chosen for main analyses. Between‐biomarker differences in explained variance were evaluated using paired bootstrap comparisons (Tables S11–S14). For comparability, these main analyses were performed in the complete case subset containing non‐missing data for all six biomarkers (*n =* 269), with similar associations found when using the maximum number of individuals available per blood biomarker (Figure S4).

In the unstratified cohort, GAMs outperformed linear models for all six plasma biomarkers when relating biomarker levels to total plaque counts (ΔAIC relative to linear models ranging from −51 to −186; Figure 2A–F, Table S9). Explained variance differed across biomarkers: p‐tau217 showed the weakest association (*R^2^ =* 0.45), followed by BD p‐tau217/p‐tau217 (*R^2^ =* 0.49) and p‐tau217/Aβ42 (*R^2^ =* 0.56). BD‐based markers generally performed better, with *R^2^ =* 0.59 for BD p‐tau217, *R^2^ =* 0.65 for BD p‐tau217/Aβ42, and *R^2^ =* 0.66 for BD P‐tau217/BD tau. Bootstrap pairwise comparisons confirmed that BD P‐tau217/Aβ42 and BD p‐tau217/BD tau each significantly outperformed p‐tau217 and p‐tau217/Aβ42 (all FDR‐adjusted p≤0.041), whereas the two top‐performing markers did not differ significantly from each other (Δ*R^2^ =* −0.014, 95% CI −0.093 to 0.065; Table S11).

When participants were stratified by neuropathological status, differences emerged (Figure 2G–L). In the Non‐AD group (*n =* 111), all biomarkers showed modest associations with plaque burden; BD‐based markers reached somewhat higher R^2^ values (up to *R^2^ =* 0.30 for BD p‐tau217/BD tau), although no significant between‐biomarker differences were identified on bootstrap testing (Table S12). In the AD group (*n =* 158), associations were substantially stronger for all biomarkers, again favoring GAMs (ΔAIC relative to linear models ranging from −18 to −74; Table S9). Explained variance ranged from *R^2^ =* 0.32 for BD p‐tau217/p‐tau217 to *R^2^ =* 0.48 for BD p‐tau217/Aβ42, with BD p‐tau217/Aβ42 performing significantly better than BD p‐tau217/p‐tau217 on bootstrap testing (Δ*R^2^ =* 0.153, 95% CI 0.052 to 0.232; FDR‐adjusted *p =* 0.030; Table S12).

All six plasma biomarkers were also associated with continuous tau tangle counts in the unstratified cohort, with GAMs again providing superior fit in all cases (ΔAIC relative to linear models ranging from −11 to −112; Figure 2 M–R, Table S10). Non‐BD markers showed lower explained variance, with *R^2^ =* 0.33 for p‐tau217 and *R^2^ =* 0.37 for p‐tau217/Aβ42. BD‐based markers demonstrated substantially stronger associations: *R^2^ =* 0.52 for BD p‐tau217, *R^2^ =* 0.55 for BD p‐tau217/Aβ42, *R^2^ =* 0.59 for BD p‐tau217/p‐tau217, and *R^2^ =* 0.60 for BD p‐tau217/BD tau. Bootstrap pairwise comparisons confirmed that all four BD‐based markers significantly outperformed both non‐BD biomarkers (FDR‐adjusted *p* < 0.001 for all comparisons involving non‐BD markers), whereas BD p‐tau217/p‐tau217 and BD p‐tau217/BD tau were jointly the strongest performers and did not differ significantly from each other (Δ*R^2^ =* −0.012, 95% CI −0.056 to 0.042; Table S13).

Stratified tangle analyses showed a distinct pattern from that observed for plaques (Figure 2S–X). In the Non‐AD group, none of the biomarkers showed meaningful associations with tangle counts, with negligible R^2^ values and no significant between‐biomarker differences on bootstrap testing (Table S14). In the AD group, GAMs again outperformed linear models for all biomarkers (ΔAIC relative to linear models ranging from −14 to −93; Table S10). Explained variance increased progressively from non‐BD to BD markers, with *R^2^ =* 0.37 for p‐tau217 and *R^2^ =* 0.42 for p‐tau217/Aβ42, rising to *R^2^ =* 0.59 for BD p‐tau217, *R^2^ =* 0.60 for BD p‐tau217/Aβ42, and *R^2^ =* 0.65 for BD p‐tau217/BD tau. The BD p‐tau217/p‐tau217 ratio showed the strongest overall association (*R^2^ =* 0.68), and bootstrap testing confirmed it significantly outperformed both non‐BD biomarkers and BD p‐tau217 (FDR‐adjusted *p* ≤ 0.028), while not significantly differing from BD p‐tau217/BD tau (Δ*R^2^ =* 0.027, 95% CI −0.031 to 0.098; *p =* 0.332; Table S14).

As a sensitivity analysis, biomarker‐pathology associations were re‐examined using linear models, with between‐biomarker differences in standardized β coefficients and R^2^ evaluated via the same bootstrap framework (Tables S15–S18; Figures S5–S6). Under linear parameterization, between‐biomarker differences were substantially attenuated and did not reach statistical significance after FDR correction, suggesting that the advantages of BD‐based markers over non‐BD markers are captured primarily through non‐linear components of their associations with neuropathological burden.

### 3.4 Discriminative accuracy for AD pathology

**FIGURE 3:**
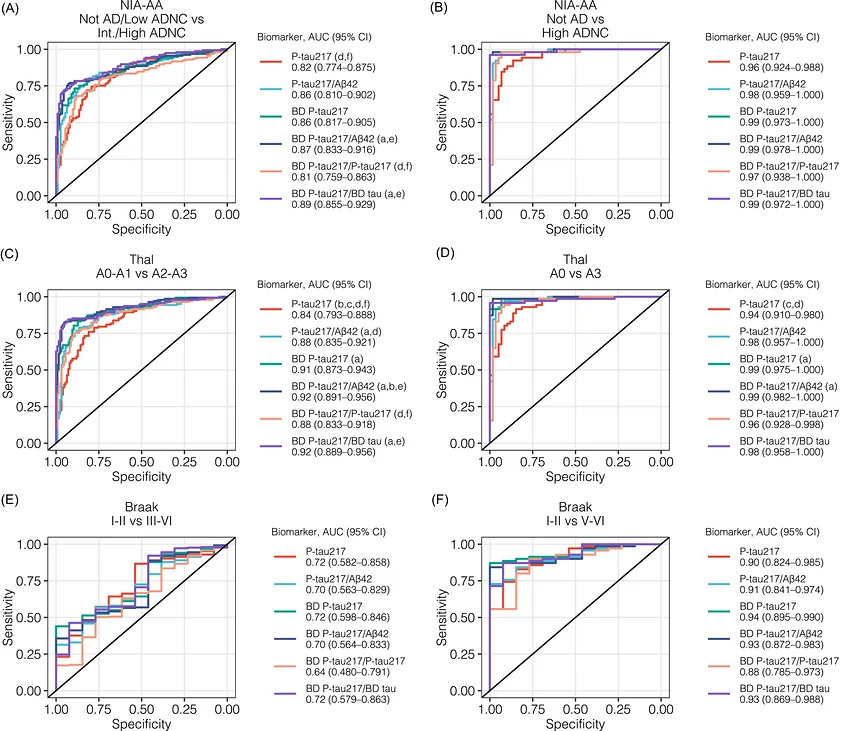
Discriminative performance of plasma biomarkers for AD neuropathology. Receiver operating characteristic curves show the performance of plasma biomarker measures for binary neuropathological classifications. Panels show classification of NIA‐AA AD neuropathologic change as Not AD/Low ADNC versus Intermediate/High ADNC (A) and Not AD versus High ADNC (B); Thal amyloid phase A0‐A1 versus A2‐A3 (C) and A0 versus A3 (D); and Braak stage I–II versus III–VI (E) and I–II versus V–VI (F). Legend entries show AUCs with 95% CIs. All analyses were performed in the complete‐case subset with non‐missing values for the six plasma biomarker measures (*n =* 268). Letters in parentheses indicate significant differences computed with FDR‐adjusted paired DeLong *p*‐values < 0.05. Significantly different from: a, p‐tau217; b, p‐tau217/Aβ42; c, BD p‐tau217; d, BD p‐tau217/Aβ42; e, BD p‐tau217/p‐tau217; f, BD p‐tau217/BD tau. AD, Alzheimer's disease; ADNC, AD neuropathological change; AUC, area under the curve; BD, brain derived; CI, confidence interval; FDR, false discovery rate; NIA‐AA, National Institute on Aging–Alzheimer's Association; p‐tau, phosphorylated tau.

The discriminative ability of all six BBMs was evaluated using ROC curves (Figure 3). For NIA‐AA ADNC classification, AUCs for the distinction between Not/Low (*n =* 111) and Intermediate/High ADNC (*n =* 157; Figure 3A, Table S19) ranged from 0.811 (BD p‐tau217/p‐tau217) to 0.892 (BD p‐tau217/BD tau, 95% CI 0.855–0.929). BD p‐tau217/p‐tau217 showed significantly lower accuracy than both BD p‐tau217/Aβ42 (0.874; *P*_FDR _= 0.026) and BD p‐tau217/BD tau (*P*_FDR_ = 0.002). When restricting to pathological extremes, Not AD (*n =* 59) versus High ADNC (*n =* 52; Figure 3B), all biomarkers performed strongly (AUCs 0.956–0.993) with no significant pairwise differences.

Significant differences in discriminative accuracy were observed for Thal amyloid phase classification. For Thal A0–A1 (*n =* 126) versus A2–A3 (*n =* 142; Figure 3C, Table S20), AUCs ranged from 0.841 (p‐tau217) to 0.924 (BD p‐tau217/Aβ42, 95% CI 0.891–0.956). The p‐tau217 was significantly outperformed by BD p‐tau217 (0.908; *P*_FDR _< 0.001), BD p‐tau217/Aβ42 (*P*_FDR_ < 0.001), and BD p‐tau217/BD tau (0.923; *P*_FDR _= 0.004). The p‐tau217/Aβ42 (0.878) was also outperformed by BD p‐tau217/Aβ42 (*P*_FDR _= 0.004). For the pathological extremes of Thal A0 (*n =* 59) versus A3 (*n =* 71; Figure 3D), all biomarkers showed high accuracy (AUCs 0.945–0.993) with no significant pairwise differences.

For Braak stage classification, discriminative accuracy was moderate for the distinction between early (I–II; *n =* 13) and any advanced pathology (III–VI; *n =* 255; Figure 3E, Table S21), with AUCs ranging from 0.635 (BD p‐tau217/p‐tau217) to 0.722 (BD p‐tau217); wide 95% CIs reflected the limited number of early‐stage participants, and no significant pairwise differences were found. When restricting to Braak I–II versus V–VI (*n =* 83; Figure 3F), all biomarkers performed strongly (AUCs 0.879–0.943), again without significant pairwise differences.

### 3.5 Baseline plasma biomarkers and longitudinal cognitive decline

Linear mixed‐effects models were used to evaluate whether baseline plasma biomarker levels modified the rate of longitudinal cognitive change. The β coefficients from the time × baseline biomarker interaction quantify the difference in annual cognitive change per one standard deviation (1 SD) higher baseline biomarker concentration. CDR‐SB was analyzed in inverted form (higher values indicating better cognition), such that negative β estimates indicate steeper longitudinal clinical deterioration to be comparable with MMSE. The longitudinal analytic sample, requiring non‐missing results for all six biomarkers at the baseline visit, included 606 CDR‐SB observations from 273 participants and 518 MMSE observations from 209 participants, with a median follow‐up of 2.41 years (IQR 1.22–3.50).

**FIGURE 4:**
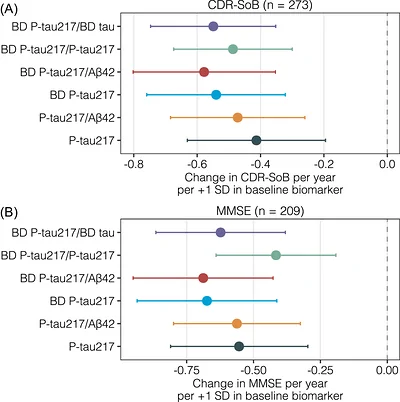
Associations between baseline plasma biomarkers and longitudinal cognitive change. Forest plots show fixed‐effect estimates and 95% CIs for the interaction between follow‐up time and baseline biomarker concentration in longitudinal mixed‐effects models of CDR‐SB (A) and MMSE (B). Estimates represent the difference in annual cognitive change per 1 SD higher baseline biomarker concentration. CDR‐SB was modeled as –CDR‐SB so that higher values indicate better cognition; therefore, for both CDR‐SB and MMSE, more negative estimates indicate faster cognitive worsening over time. Models included follow‐up time, baseline biomarker, their interaction, age, years of education, sex, and a participant‐level random intercept and random slope for time. Analyses used the longitudinal complete‐case subset, defined separately from the pathology complete‐case subset: participants were required to have complete baseline data for all six blood biomarker variables, non‐missing age and years of education, and an available cognitive measure at the first visit for the relevant outcome, but neuropathology variables such as NIA‐AA ADNC, Braak stage, and Thal phase were not required. The effective participant sample size is shown in each panel title. AD, Alzheimer's disease; ADNC, AD neuropathological change; CDR‐SB, Clinical Dementia Rating Sum of Boxes; CI, confidence interval; MMSE, Mini‐Mental State Examination; NIA‐AA, National Institute on Aging–Alzheimer's Association; SD, standard deviation.

Higher baseline levels were consistently associated with faster subsequent cognitive decline across all six biomarkers, with significant negative time × baseline biomarker interactions for both CDR‐SB and MMSE (all *p*’s* <* 0.001; Table S22). For CDR‐SB (Figure 4A), BD‐based biomarkers showed numerically larger effect sizes than their non‐BD counterparts. The strongest associations were observed for BD p‐tau217/Aβ42 (*β =* −0.578, 95% CI −0.802 to −0.353), BD p‐tau217/BD tau (*β =* −0.549, 95% CI −0.747 to −0.352), and BD p‐tau217 (*β =* −0.540, 95% CI −0.759 to −0.322), exceeding BD p‐tau217/p‐tau217 (*β =* −0.487, 95% CI −0.674 to −0.300), p‐tau217/Aβ42 (*β =* −0.472, 95% CI −0.684 to −0.260), and p‐tau217 (*β =* −0.413, 95% CI −0.631 to −0.195). For MMSE (Figure 4B), BD p‐tau217/Aβ42 (*β =* −0.688, 95% CI −0.950 to −0.427) and BD p‐tau217 (*β =* −0.674, 95% CI −0.935 to −0.413) again showed the largest effects, followed by BD p‐tau217/BD tau (*β =* −0.623, 95% CI −0.865 to −0.381), p‐tau217/Aβ42 (*β =* −0.562, 95% CI −0.799 to −0.325), p‐tau217 (*β =* −0.554, 95% CI −0.810 to −0.297), and BD p‐tau217/p‐tau217 (*β =* −0.416, 95% CI −0.640 to −0.192).

Model fit metrics consistently favored BD biomarkers (Table S23). For CDR‐SB, AIC was lowest for BD p‐tau217/Aβ42 (2980, ΔAIC = −36 vs p‐tau217) and BD p‐tau217 (2982, ΔAIC = −34), followed by BD p‐tau217/BD tau (2986), BD p‐tau217/p‐tau217 (2989), and p‐tau217/Aβ42 (3004). Marginal R^2^ increased progressively from 0.161 (p‐tau217) and 0.203 (p‐tau217/Aβ42) to 0.225 (BD p‐tau217), 0.228 (BD p‐tau217/BD tau), 0.237 (BD p‐tau217/Aβ42), and 0.255 (BD p‐tau217/p‐tau217). For MMSE, BD p‐tau217/Aβ42 achieved the lowest AIC (2840, ΔAIC = −65), followed by BD p‐tau217 (2846, ΔAIC = −59), BD p‐tau217/BD tau (2848, ΔAIC = −57), and BD p‐tau217/p‐tau217 (2857, ΔAIC = −49), compared with p‐tau217/Aβ42 (2870, ΔAIC = −35) and p‐tau217 (2877, ΔAIC = −28). Marginal R^2^ increased from 0.199 (p‐tau217) and 0.236 (p‐tau217/Aβ42) to 0.284 (BD p‐tau217/p‐tau217 and BD p‐tau217/BD tau), 0.298 (BD p‐tau217), and 0.328 (BD p‐tau217/Aβ42).

Bootstrap pairwise comparisons were consistent with the general pattern of BD biomarker superiority, although none reached statistical significance after FDR correction for either outcome (all *P*_FDR_ > 0.05; Tables S24–S25). For CDR‐SB, bootstrap 95% CIs for the β difference between p‐tau217 and BD p‐tau217 (Δ*β =* 0.127, 95% CI 0.031–0.240) and between p‐tau217 and BD p‐tau217/Aβ42 (Δ*β =* 0.165, 95% CI 0.023–0.354) excluded zero, suggesting a potential advantage possibly discarded by FDR correction. For MMSE, the largest R^2^ differences relative to p‐tau217 were observed for BD p‐tau217/Aβ42 (ΔR^2^ = −0.129) and BD p‐tau217 (Δ*R^2^ =* −0.099), although again not reaching FDR‐corrected significance.

### 3.6 Tau‐PET, plasma biomarkers, and post‐mortem tangle pathology

**FIGURE 5:**
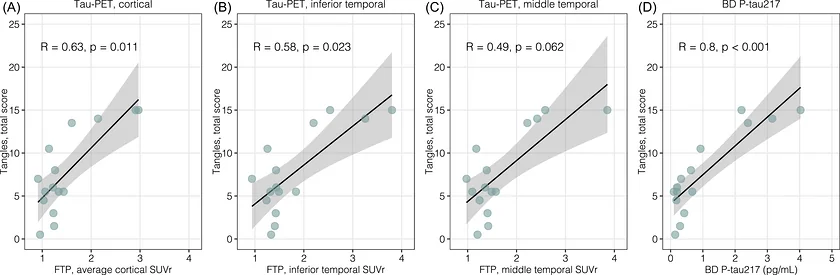
Associations of tau‐PET and BD p‐tau217 with neurofibrillary tangle burden in the tau‐PET subset. Scatterplots show associations between neurofibrillary tangle burden and tau‐PET measures or BD p‐tau217 in participants with available tau‐PET data. Panels show associations of total tangle score with average cortical FTP SUVR (A), inferior temporal FTP SUVR (B), middle temporal FTP SUVR (C), and plasma BD p‐tau217 concentration (D). Lines represent fitted linear trends and shaded regions represent 95% confidence intervals. Spearman correlation coefficients and *p*‐values are shown within each panel. Analyses were performed in the tau‐PET subset (*n =* 15), restricted to participants with available tau‐PET, total tangle score, and the biomarker or imaging measure shown in the panel. BD, brain‐derived; FTP, flortaucipir; p‐tau, phosphorylated tau; PET, positron emission tomography; SUVR = standardized uptake value ratio.

A Spearman correlation analysis was conducted in a subset of 15 participants to comparatively assess how plasma biomarkers and antemortem tau‐PET signal (inferior and middle temporal regions; also an average cortical region for closer correspondence with what is measured by the tangle total score) measured using [^18^F]‐flortaucipir relate to post‐mortem tangle pathology (Figure 5). For this analysis, rather than using the sample closest to end‐of‐life as in previous analyses, we used, when available, the sample closest to the tau‐PET scan. Median (IQR) absolute time difference between tau‐PET scan and BBM measurement was 0.15 (0.12–0.66) years. At ADNC autopsy assessment, *n =* 6 were Not AD, *n =* 2 were Low ADNC, *n =* 4 were Intermediate ADNC, and *n =* 3 were High ADNC.

In this sub‐sample, the average cortical tau‐PET SUVR showed a Spearman's rho of 0.63 (*p =* 0.011; Figure 5A) with tangle score, whereas the correlation for inferior temporal tau‐PET SUVR was 0.58 (*p =* 0.023; Figure 5B) and 0.49 (*p =* 0.062; Figure 5C) for middle temporal SUVR. In contrast, plasma BD p‐tau217, which was the BBM with the highest association with tangles in this subset, showed a significant correlation of 0.80 (*p <* 0.001; Figure 5D). Other BBMs also showed robust monotonic positive associations with tangle score (Figure S7), ranging from 0.50 for BD p‐tau217/p‐tau217 to 0.74 for p‐tau217/Aβ42, most presenting as numerically higher than those observed for tau‐PET measures.

When evaluating BBM associations with tau‐PET SUVR in the three ROIs, moderate‐to‐strong relationships were found (Figure S8). For the average cortical tau‐PET SUVR, Spearman's rho ranged from 0.54 for BD p‐tau217/p‐tau217 to 0.71 for BD p‐tau217 and BD p‐tau217/Aβ42. For the inferior temporal tau‐PET SUVR, significant Spearman associations ranged from 0.54 for BD p‐tau217/BD tau to 0.65 for p‐tau217/Aβ42, with BD p‐tau217/p‐tau217 not showing a significant association (*p* > 0.05). For the middle temporal tau‐PET SUVR, Spearman's rho ranged from 0.50 for p‐tau217/Aβ42 to 0.65 for BD p‐tau217/Aβ42.

### 3.7 Plasma biomarkers and non‐AD co‐pathologies

As an exploratory sensitivity analysis, we examined associations between plasma biomarkers and four common non‐AD co‐pathologies—CAA, Lewy body pathology, TDP‐43 pathology, and vascular pathology—using nested adjustment models to probe whether any observed associations were independent of AD pathological burden (Figure S9).

For CAA, all six biomarkers showed significant positive associations in unadjusted models and after adjustment for age, sex, and plasma–autopsy interval (all *p <* 0.001; Table S26). However, these associations were uniformly attenuated to non‐significance upon additional adjustment for plaque burden (all *p*’s* >* 0.05), and remained non‐significant after further adjustment for tangle burden. This pattern was consistent across all biomarkers, suggesting that the apparent association between CAA and plasma p‐tau217–based biomarkers is likely mediated by co‐occurring amyloid plaque pathology rather than reflecting an independent effect of CAA. For Lewy body pathology, no biomarker showed a nominally significant association at any level of adjustment (all *p*’s* >* 0.05; Table S27), with effect estimates close to zero across the adjustment ladder. For TDP‐43 pathology, significant positive associations were observed in unadjusted models and after age, sex, and interval adjustment for all six biomarkers (Table S28). For p‐tau217 (*β =* 0.364, 95% CI 0.101–0.626, *p =* 0.007) and p‐tau217/Aβ42 (*β =* 0.363, 95% CI 0.101–0.626, *p =* 0.007), associations were attenuated after adjustment for plaque burden alone. In contrast, BD‐based biomarkers showed more persistent associations, remaining nominally significant after plaque adjustment (e.g., BD p‐tau217/BD tau: *β =* 0.257, 95% CI 0.078–0.435, *p =* 0.005; BD p‐tau217: *β =* 0.209, 95% CI 0.020–0.399, *p =* 0.030), and were only fully attenuated after additional adjustment for tangle burden. For vascular pathology, no biomarker showed a significant association at any adjustment level (all *p*’s* >* 0.05; Table S29), with effect estimates consistently near zero and CIs spanning zero throughout.

## 4 DISCUSSION

This study leveraged a large clinicopathological cohort to perform a comparison of plasma p‐tau217 and its novel BD variant, together with their Aβ42 ratios and the BD p‐tau217/BD tau ratio, quantified in a random‐access, fully automated platform for research use only. Across all analyses, the BBMs robustly reflected the core pathological hallmarks of AD, showing strong associations with increasing severity of ADNC, Thal amyloid phase, and Braak tau stage. These results reinforce the growing body of evidence establishing plasma p‐tau217 as a highly accurate and disease‐specific marker of biological AD. Furthermore, despite the limited sample size for tau‐PET analyses, all six BBMs presented greater associations with post‐mortem tangle burden than tau‐PET did.

Our most notable finding was the markedly broader dynamic range of the BD p‐tau217 variants. When comparing individuals with High ADNC to those without AD, BD markers showed nearly a twofold greater fold‐change (FC) when compared with non‐BD p‐tau217 (BD p‐tau217/Aβ42, FC ≈ 8.3 vs p‐tau217/Aβ42, FC ≈ 3.8), with differences that were statistically supported by bootstrap pairwise comparisons (all *P*_FDR_ < 0.001). This enhanced separation persisted across both Thal phases and Braak stages. In contrast, the ratio of BD p‐tau217 to total p‐tau217 exhibited the lowest fold‐change (FC ≈ 2.2), suggesting that while isolating the BD fraction expands the dynamic range, normalizing it to the total phosphorylated pool compresses this signal, likely due to the simultaneous elevation of both isoforms in advanced disease. The magnitude of fold‐change is a critical determinant of biomarker robustness and clinical translatability, as it reduces susceptibility to pre‐analytical variability and minimizes overlap between diagnostic groups.^5^, ^30^ Accordingly, the larger dynamic range of BD p‐tau217 variants suggests improved differentiation potential. If further validated in larger studies with good analytical performance, this could reduce the proportion of cases falling into uncertain ranges that necessitate confirmatory PET or CSF testing.^31^ Whether this translates to fewer equivocal cases in clinical workflows requires prospective validation in living, intended‐use populations.

Despite these differences in magnitude, discriminative accuracy for ADNC was good‐to‐excellent in most cases. Nevertheless, the BD variants consistently yielded the highest numerical estimates across nearly all comparisons and reached statistical superiority over conventional p‐tau217 for amyloid‐related discrimination, with BD p‐tau217/BD tau showing comparable discriminative accuracy to BD p‐tau217/Aβ42 for ADNC and Thal phase classifications. They also exhibited the strongest correlations with continuous post‐mortem plaque and tangle scores using GAMs, which consistently provided a better fit than linear models. When associations were re‐examined under a strictly linear parameterization, between‐biomarker differences were substantially attenuated and did not reach statistical significance after FDR correction, indicating that the advantage of BD‐based markers may be captured primarily through non‐linear components of their relationship with neuropathological burden. Although the BD p‐tau217/p‐tau217 ratio showed lower discriminative ability for pathology and fold‐changes, it demonstrated the highest numerical association with neurofibrillary tangle counts (*R^2^ =* 0.68). This suggests that although the BD‐specific fraction drives higher dynamic ranges, ratios that normalize BD p‐tau217 to either total p‐tau217 or total BD tau may better reflect neocortical tau pathology burden. Both ratio approaches were included exploratorily, as potentially internally normalized metrics more reflective of the relative brain‐derived contribution to circulating p‐tau217 levels. Ratio‐based approaches are used increasingly in AD BBM research (e.g., p‐tau/Aβ42, p‐tau217/np‐tau217) to improve biological specificity, minimize confounder effect, and improve analytical robustness, and a p‐tau217/BD‐tau ratio has recently been proposed as a strategy to further enhance biomarker stability.^32^, ^33^, ^34^, ^35^

Longitudinal linear mixed‐effects models further underscored the prognostic utility of BD variants. Although all biomarkers’ baseline levels significantly predicted subsequent cognitive decline, models incorporating BD p‐tau217 provided the best overall statistical fit (lower AIC, higher R^2^) relative to those using non‐BD p‐tau217. Together, these findings indicate that isolating the BD fraction of p‐tau217 may enhance its disease specificity and statistical prognostic potential.

The tau‐PET findings provide complementary validation despite the small imaging subset (*n =* 15) but unique in its composition of *head‐to‐head* tau‐PET and plasma biomarker against neuropathological confirmation. Plasma biomarkers, particularly BD p‐tau217, showed stronger associations with post‐mortem tangle burden than regional [^1^ ^8^F]‐flortaucipir uptake, with BD p‐tau217 reaching the highest correlation (ρ = 0.80), whereas tau‐PET SUVR showed weaker relationships with tangles (ρ = 0.63 for average cortical; ρ = 0.58 for inferior temporal; ρ = 0.49 for middle temporal). Other plasma biomarkers also demonstrated meaningful associations with tangles (ρ = 0.71–0.74) that were numerically higher than those observed for tau‐PET, suggesting that plasma measures may be more sensitive to lower or intermediate tangle burden than tau‐PET, given that only 3 of 15 participants in this subset had a High ADNC stage diagnosis. These results were generated in a small subset of the cohort and must be interpreted cautiously, with larger studies comparing tau‐PET and plasma biomarkers against post‐mortem results needed to further evaluate relative sensitivity across disease stage.

To probe the biological specificity of BD‐based markers, we performed exploratory analyses of associations between plasma biomarkers and four common non‐AD co‐pathologies. No biomarker was associated with Lewy body or vascular pathology at any level of adjustment. Apparent associations with CAA were fully attenuated after adjustment for plaque burden, consistent with mediation by co‐occurring amyloid pathology rather than an independent CAA‐related signal. For TDP‐43 pathology, both non‐BD and BD‐based biomarkers showed positive associations in age‐ and sex‐adjusted models; however, associations for non‐BD p‐tau217 markers were attenuated upon adjustment for plaque burden alone, whereas BD‐based biomarkers retained nominally significant associations after plaque adjustment and were only fully attenuated after additional adjustment for tangle burden. Although exploratory, this pattern suggests that the BD signal may track more closely with the tangle‐related component of mixed pathologies, and further work in larger samples is warranted to determine whether this reflects biologically meaningful co‐pathology sensitivity or residual confounding by AD burden.

There is a growing body of evidence establishing plasma p‐tau217 as a highly accurate and disease‐specific marker of biological AD, with regulatory‐ approved tests available for clinical use in certain jurisdictions and intended‐use populations. Nonetheless, recent studies have identified modest, nonspecific elevations of peripheral p‐tau217 in some non‐AD disorders—most prominently in ALS—suggesting that conventional p‐tau217 may be partially influenced by peripheral or non‐AD neurodegenerative processes.^14^, ^15^, ^16^, ^17^ Of note, isolating the BD fraction has the potential to minimize these peripheral contributions, thereby improving the detection of AD‐specific signal and reducing diagnostic ambiguity.^16^ This has been demonstrated by a lower number of individual elevations in BD p‐tau217 compared to p‐tau217 in participants with no or low ADNC. Further work in clinical populations should continue to evaluate the p‐tau/Aβ42 ratios in plasma, since it is known that plasma Aβ42 presents more vulnerability to different sources of pre‐analytical, analytical, and biological sources of variation.^30^, ^36^, ^37^, ^38^

A major strength of this study is the use of a large, well‐characterized clinicopathological cohort with standardized post‐mortem assessment across multiple AD neuropathological scales and longitudinal ante‐mortem follow‐up. We have utilized novel and fully automated assay versions of p‐tau217, BDp‐tau217, and BD‐tau, which have direct clinical application over research grade or multiplex assays that include BD tau217. Several limitations should also be acknowledged. First, our tau‐PET analyses were available only in a small subset, limiting statistical power and requiring cautious interpretation of between‐biomarker comparisons. Second, this was an *end‐of‐life* cohort, and although it provides unparalleled pathological validation, performance estimates may differ in intended‐use and younger clinical populations.

In summary, this study demonstrates that adapting plasma p‐tau217 assays to selectively quantify BD variants, as well as BD tau, may improve their dynamic range across neuropathological strata while maintaining strong discriminative performance, without evidence of reduced association with neuropathology. BD p‐tau217 and its Aβ42 ratio emerge as promising candidates for translation into broader diagnostic and therapeutic monitoring applications, with the BD p‐tau217/BD tau ratio representing an additional candidate that warrants further evaluation. Future work should assess performance in intended‐use clinical settings, evaluate potential confounding by non‐AD comorbidities (including neuromuscular disease), and determine whether improved dynamic range translates into meaningful gains in classification efficiency or monitoring in therapeutic contexts.

## CONFLICT OF INTEREST STATEMENT

K.B., C.M.C., and K.H. are employees of Beckman Coulter. E.M.R. reports his institution received support for this work from the John & Tami Marick (JTMF) Foundation, GHR Foundation, Banner Alzheimer's Foundation, and Arizona Department of Health Services (AZ DHS); reports institutional grants or contracts with the following entities: National Institutes of Health (NIH) (Grants R01 AG058468, R01 AG086363, R01 AG055444, P30 AG072980, P30 AG019620, R01 AG069453, R01 NS 139383, OT2 D026549, OT2 OD037642, T2 HL161847, P01 AG052350, R01 AG070883, RF1 AG073424, U24 AG072122, and R01AG05467), JTMF Foundation, GHR Foundation, NOMIS Foundation, Banner Alzheimer's Foundation, Gates Ventures, and AZ DHS; reports consulting fees and/or stock options for serving as a scientific advisor to Alzheon, Beren Therapeutics, Cognition Therapeutics, Denali, Enigma, Jocasta Neuroscience, Retromer Therapeutics, and Vaxxinity; reports being the inventor of a 2005 patent issued to Banner Health for using biomarker endpoints to accelerate the evaluation Alzheimer's prevention therapies in people who are cognitively unimpaired at genetic or biomarker risk, which will not be deployed; reports serving as Board Chairman of the Flinn Foundation and the Arizona Alzheimer's Consortium; and reports having stock shares as a cofounder of ALZPath, maker of a capture antibody that has been licensed to several diagnostics companies for the performance of plasma p‐tau217 assays. N.J.A. reports payment for serving on scientific advisory boards, educational lectures and/or as a consultant for AbbVie, Alamar Biosciences, Beckman Coulter, Danaher, Eli Lilly, ImmunoBrain, New Amsterdam Therapeutics, Quanterix, Roche, and Spear Bio. All other authors declare no conflicts of interest. Author disclosures are available in the Supporting Information.

## CONSENT STATEMENT

All subjects provided written informed consent.

## Supporting information




**Supporting Information**: alz71773‐sup‐0001‐SuppMat.docx

**Supporting Information**: alz71773‐sup‐0002‐Disclosureforms.pdf
