# Supplementary material for: Brain‐derived plasma p‐tau217 shows enhanced dynamic range for Alzheimer's disease neuropathological change
Source: Alzheimers Dement. 2026 Sep 1;22(9):e71773. doi: 10.1002/alz.71773 (PMC13531372; doi:10.1002/alz.71773)
Supplement: Supplementary file 2 [file ALZ-22-e71773-s002.docx]

**Supplementary Table 1.** Analytical measuring range and within-laboratory imprecision for the plasma biomarker assays used in this study.

| **Assay** | **Analytical Measuring Range (pg/mL)*** | **Within-laboratory Imprecision (%CV)**** |
| --- | --- | --- |
| Access p-Tau217 (RUO) | 0.03 – 8 | 3.0 – 9.0 |
| Access β-Amyloid 1-42 (RUO) | 0.2 – 250 | 3.6 – 4.9 |
| Access BD-Tau (RUO) | 0.13 – 300 | 1.9 – 2.8 |
| Prototype BD p-tau217† | 0.03 – 10 | 3.2 – 7.3 |

This table summarizes the analytical measuring range and representative within-laboratory imprecision for the four plasma biomarker assays used to generate the biomarker measures analyzed in this study, all performed on the DxI 9000 Access Immunoassay Analyzer (Beckman Coulter). The Access p-Tau217 (RUO) and the prototype BD p-tau217 assays are two-step immunometric (sandwich) immunoassays that share the same anti-p-Tau217 capture antibody and differ in their detection antibody, with the prototype BD p-tau217 assay using a detection antibody directed at the exon 4/exon 5 junction of tau to preferentially capture brain-derived tau isoforms.

*The low end of the analytical measuring range corresponds to the 20% CV limit of quantitation estimate derived from a single representative study on the DxI 9000 Access Immunoassay Analyzer based on CLSI EP17-A2.
**Imprecision values represent the range of representative within-laboratory imprecision observed for samples reflecting the approximate concentrations measured in the present cohort. †The BD p-tau217 assay was a prototype at the time of this study; derived absolute concentrations (i.e., pg/mL values) are therefore subject to change. All biomarker measurements obtained in the present cohort fell within the analytical measuring range of each respective assay.

**Supplementary Table 2.** Participant information for the complete-case subset.

| **Characteristic** | **Not AD N = 59^1^** | **Low ADNC N = 52^1^** | **Int. ADNC N = 106^1^** | **High ADNC N = 52^1^** | **Overall N = 269^1^** |
| --- | --- | --- | --- | --- | --- |
| **Age at death (years)** | 86.0 (79.0–92.0) | 82.5 (77.0–89.5) | 87.0 (82.0–93.0) | 85.0 (78.5–89.5) | 86.0 (80.0–92.0) |
| **Female** | 30 (50.8%) | 22 (42.3%) | 49 (46.2%) | 20 (38.5%) | 121 (45.0%) |
| **APOEε4 carriers** | 3 (5.1%) | 13 (25.0%) | 29 (27.4%) | 30 (57.7%) | 75 (27.9%) |
| **Years of education** | 16.0 (14.0–16.0) | 16.0 (13.5–18.0) | 16.0 (13.0–18.0) | 14.0 (12.0–16.0) | 16.0 (13.0–18.0) |
| **Interval between last blood draw and autopsy (years)** | 1.1 (0.6–2.6) | 1.0 (0.7–1.8) | 1.8 (0.9–2.8) | 1.8 (0.7–3.0) | 1.4 (0.7–2.7) |
| **Last MMSE** | 27.0 (23.0–28.0) | 27.0 (24.0–28.0) | 26.0 (22.0–28.0) | 11.0 (5.0–23.0) | 25.0 (20.0–28.0) |
| **Last CDR-SoB** | 1.0 (0.0–5.0) | 1.0 (0.0–2.5) | 1.0 (0.0–5.0) | 11.0 (5.0–15.0) | 2.0 (0.0–7.5) |
| **Co-pathology, CAA** | 6 (10.2%) | 29 (55.8%) | 74 (69.8%) | 50 (96.2%) | 159 (59.1%) |
| **Co-pathology, Lewy body** | 23 (39.0%) | 23 (46.0%) | 45 (44.6%) | 27 (51.9%) | 118 (45.0%) |
| **Co-pathology, TDP-43** | 14 (25.9%) | 9 (18.4%) | 29 (27.9%) | 28 (58.3%) | 80 (31.4%) |
| **Co-pathology, vascular** | 29 (49.2%) | 20 (38.5%) | 51 (48.1%) | 21 (40.4%) | 121 (45.0%) |

Participant information is presented according to NIA-AA ADNC stages for the subset of donors with complete data for all six blood biomarker variables used in the study: P-tau217, P-tau217/Aβ42, BD P-tau217, BD P-tau217/Aβ42, BD P-tau217/P-tau217, and BD P-tau217/BD tau. Continuous variables are summarised as median (interquartile range); categorical variables as n (%). Last MMSE and CDR-SoB are taken from the latest available longitudinal visit. ADNC = Alzheimer's disease neuropathologic change; CAA = cerebral amyloid angiopathy; CDR-SoB = Clinical Dementia Rating Sum of Boxes; MMSE = Mini-Mental State Examination; TDP-43 = transactive response DNA-binding protein 43.

**Supplementary Table 3.** Observed adjusted fold-changes according to NIA-AA Alzheimer's disease neuropathologic change.

| **Biomarker** | **Low ADNC** | **Intermediate ADNC** | **High ADNC** |
| --- | --- | --- | --- |
| P-tau217 | 1.11 (0.92, 1.35) | 1.74 (1.47, 2.06) | 3.37 (2.77, 4.10) |
| P-tau217/Aβ42 | 1.11 (0.91, 1.34) | 1.91 (1.62, 2.25) | 3.77 (3.11, 4.58) |
| BD P-tau217 | 1.27 (1.00, 1.61) | 2.42 (1.98, 2.97) | 7.38 (5.81, 9.37) |
| BD P-tau217/Aβ42 | 1.27 (1.00, 1.60) | 2.66 (2.17, 3.25) | 8.26 (6.51, 10.47) |
| BD P-tau217/P-tau217 | 1.14 (1.00, 1.30) | 1.39 (1.25, 1.56) | 2.19 (1.92, 2.50) |
| BD P-tau217/BD tau | 1.16 (0.96, 1.41) | 2.21 (1.88, 2.60) | 5.26 (4.35, 6.38) |

Fold-changes are expressed relative to the Not AD reference group for each NIA-AA Alzheimer's disease neuropathologic change category. Estimates and 95% CIs were obtained from log-transformed linear models adjusted for age at death, sex, and the interval between last plasma sampling and autopsy, then exponentiated for presentation. Analyses used the common complete-case subset with non-missing outcome data, non-missing covariates, and non-missing positive values for all six plasma biomarkers. The effective analytic sample size was n=269.

**Supplementary Table 4.** Bootstrapped comparison of biomarker fold-change ratios according to NIA-AA Alzheimer's disease neuropathologic change.

| **Biomarker 1** | **Biomarker 2** | **FC ratio** | **95% CI** | **FDR p** |
| --- | --- | --- | --- | --- |
| **Low ADNC** | | | | |
| P-tau217 | P-tau217/Aβ42 | 1.0031 | 0.910 to 1.105 | 0.957 |
| P-tau217 | BD P-tau217 | 0.8756 | 0.781 to 0.989 | **0.044** |
| P-tau217 | BD P-tau217/Aβ42 | 0.8784 | 0.759 to 1.017 | 0.120 |
| P-tau217 | BD P-tau217/P-tau217 | 0.9736 | 0.770 to 1.247 | 0.858 |
| P-tau217 | BD P-tau217/BD tau | 0.9578 | 0.799 to 1.159 | 0.743 |
| P-tau217/Aβ42 | BD P-tau217 | 0.8729 | 0.746 to 1.028 | 0.133 |
| P-tau217/Aβ42 | BD P-tau217/Aβ42 | 0.8756 | 0.781 to 0.989 | **0.044** |
| P-tau217/Aβ42 | BD P-tau217/P-tau217 | 0.9706 | 0.773 to 1.245 | 0.858 |
| P-tau217/Aβ42 | BD P-tau217/BD tau | 0.9548 | 0.809 to 1.141 | 0.713 |
| BD P-tau217 | BD P-tau217/Aβ42 | 1.0031 | 0.910 to 1.105 | 0.957 |
| BD P-tau217 | BD P-tau217/P-tau217 | 1.1119 | 0.935 to 1.337 | 0.263 |
| BD P-tau217 | BD P-tau217/BD tau | 1.0938 | 0.944 to 1.284 | 0.268 |
| BD P-tau217/Aβ42 | BD P-tau217/P-tau217 | 1.1085 | 0.942 to 1.322 | 0.268 |
| BD P-tau217/Aβ42 | BD P-tau217/BD tau | 1.0904 | 0.960 to 1.248 | 0.206 |
| BD P-tau217/P-tau217 | BD P-tau217/BD tau | 0.9837 | 0.862 to 1.120 | 0.858 |
| **Int. ADNC** | | | | |
| P-tau217 | P-tau217/Aβ42 | 0.9125 | 0.839 to 0.989 | **0.041** |
| P-tau217 | BD P-tau217 | 0.7178 | 0.643 to 0.802 | **<0.001** |
| P-tau217 | BD P-tau217/Aβ42 | 0.6550 | 0.570 to 0.751 | **<0.001** |
| P-tau217 | BD P-tau217/P-tau217 | 1.2491 | 1.030 to 1.515 | **0.041** |
| P-tau217 | BD P-tau217/BD tau | 0.7879 | 0.668 to 0.936 | **0.007** |
| P-tau217/Aβ42 | BD P-tau217 | 0.7866 | 0.688 to 0.900 | **<0.001** |
| P-tau217/Aβ42 | BD P-tau217/Aβ42 | 0.7178 | 0.643 to 0.802 | **<0.001** |
| P-tau217/Aβ42 | BD P-tau217/P-tau217 | 1.3689 | 1.126 to 1.661 | **<0.001** |
| P-tau217/Aβ42 | BD P-tau217/BD tau | 0.8635 | 0.743 to 1.009 | 0.103 |
| BD P-tau217 | BD P-tau217/Aβ42 | 0.9125 | 0.839 to 0.989 | **0.041** |
| BD P-tau217 | BD P-tau217/P-tau217 | 1.7402 | 1.493 to 2.034 | **<0.001** |
| BD P-tau217 | BD P-tau217/BD tau | 1.0977 | 0.969 to 1.249 | 0.191 |
| BD P-tau217/Aβ42 | BD P-tau217/P-tau217 | 1.9070 | 1.636 to 2.227 | **<0.001** |
| BD P-tau217/Aβ42 | BD P-tau217/BD tau | 1.2030 | 1.080 to 1.350 | **0.002** |
| BD P-tau217/P-tau217 | BD P-tau217/BD tau | 0.6308 | 0.560 to 0.712 | **<0.001** |
| **High ADNC** | | | | |
| P-tau217 | P-tau217/Aβ42 | 0.8934 | 0.817 to 0.978 | **0.024** |
| P-tau217 | BD P-tau217 | 0.4569 | 0.413 to 0.506 | **<0.001** |
| P-tau217 | BD P-tau217/Aβ42 | 0.4082 | 0.357 to 0.468 | **<0.001** |
| P-tau217 | BD P-tau217/P-tau217 | 1.5402 | 1.257 to 1.890 | **<0.001** |
| P-tau217 | BD P-tau217/BD tau | 0.6402 | 0.536 to 0.767 | **<0.001** |
| P-tau217/Aβ42 | BD P-tau217 | 0.5115 | 0.448 to 0.586 | **<0.001** |
| P-tau217/Aβ42 | BD P-tau217/Aβ42 | 0.4569 | 0.413 to 0.506 | **<0.001** |
| P-tau217/Aβ42 | BD P-tau217/P-tau217 | 1.7240 | 1.423 to 2.085 | **<0.001** |
| P-tau217/Aβ42 | BD P-tau217/BD tau | 0.7166 | 0.615 to 0.841 | **<0.001** |
| BD P-tau217 | BD P-tau217/Aβ42 | 0.8934 | 0.817 to 0.978 | **0.024** |
| BD P-tau217 | BD P-tau217/P-tau217 | 3.3706 | 2.831 to 4.023 | **<0.001** |
| BD P-tau217 | BD P-tau217/BD tau | 1.4011 | 1.202 to 1.626 | **<0.001** |
| BD P-tau217/Aβ42 | BD P-tau217/P-tau217 | 3.7729 | 3.200 to 4.431 | **<0.001** |
| BD P-tau217/Aβ42 | BD P-tau217/BD tau | 1.5683 | 1.385 to 1.788 | **<0.001** |
| BD P-tau217/P-tau217 | BD P-tau217/BD tau | 0.4157 | 0.368 to 0.473 | **<0.001** |

Fold-changes are expressed relative to the Not AD reference group for each NIA-AA Alzheimer's disease neuropathologic change category. Biomarker log fold-changes were estimated from log-transformed models adjusted for age at death, sex, and the interval between last plasma sampling and autopsy. For this neuropathological outcome, analyses used the maximum complete-case sample with non-missing outcome data, non-missing covariates, and non-missing positive values for all six biomarkers, allowing direct pairwise comparison of biomarker fold-changes within this outcome. All biomarker models were fitted simultaneously on each resampled dataset so that pairwise differences are paired by construction; 95% confidence intervals and Benjamini-Hochberg FDR-adjusted bootstrap p-values are shown. Displayed fold-change ratios and 95% CIs are exponentiated paired differences in log fold-changes; values greater than 1 indicate a larger fold-change for biomarker 1 than biomarker 2. Bootstrap p-values are based on paired differences on the log fold-change scale.The effective analytic sample size for this analysis was n=269.

**Supplementary Table 5.** Observed adjusted fold-changes according to Thal amyloid phase.

| **Biomarker** | **A1** | **A2** | **A3** |
| --- | --- | --- | --- |
| P-tau217 | 1.14 (0.95, 1.36) | 1.74 (1.46, 2.09) | 2.98 (2.49, 3.57) |
| P-tau217/Aβ42 | 1.14 (0.95, 1.36) | 1.92 (1.61, 2.28) | 3.34 (2.80, 3.99) |
| BD P-tau217 | 1.20 (0.97, 1.49) | 2.57 (2.08, 3.18) | 6.05 (4.89, 7.49) |
| BD P-tau217/Aβ42 | 1.20 (0.98, 1.49) | 2.83 (2.30, 3.47) | 6.79 (5.52, 8.35) |
| BD P-tau217/P-tau217 | 1.06 (0.94, 1.19) | 1.48 (1.31, 1.66) | 2.03 (1.81, 2.28) |
| BD P-tau217/BD tau | 1.15 (0.97, 1.37) | 2.27 (1.91, 2.68) | 4.56 (3.85, 5.40) |

Fold-changes are expressed relative to the A0 reference group for each Thal amyloid phase category. Estimates and 95% CIs were obtained from log-transformed linear models adjusted for age at death, sex, and the interval between last plasma sampling and autopsy, then exponentiated for presentation. Analyses used the common complete-case subset with non-missing outcome data, non-missing covariates, and non-missing positive values for all six plasma biomarkers. The effective analytic sample size was n=268.

**Supplementary Table 6.** Bootstrapped comparison of biomarker fold-change ratios according to Thal amyloid phase.

| **Biomarker 1** | **Biomarker 2** | **FC ratio** | **95% CI** | **FDR p** |
| --- | --- | --- | --- | --- |
| **A1** | | | | |
| P-tau217 | P-tau217/Aβ42 | 0.9996 | 0.914 to 1.091 | 0.966 |
| P-tau217 | BD P-tau217 | 0.9429 | 0.841 to 1.063 | 0.428 |
| P-tau217 | BD P-tau217/Aβ42 | 0.9425 | 0.826 to 1.082 | 0.512 |
| P-tau217 | BD P-tau217/P-tau217 | 1.0701 | 0.849 to 1.343 | 0.659 |
| P-tau217 | BD P-tau217/BD tau | 0.9839 | 0.831 to 1.162 | 0.880 |
| P-tau217/Aβ42 | BD P-tau217 | 0.9433 | 0.813 to 1.108 | 0.571 |
| P-tau217/Aβ42 | BD P-tau217/Aβ42 | 0.9429 | 0.841 to 1.063 | 0.428 |
| P-tau217/Aβ42 | BD P-tau217/P-tau217 | 1.0705 | 0.853 to 1.361 | 0.659 |
| P-tau217/Aβ42 | BD P-tau217/BD tau | 0.9843 | 0.844 to 1.154 | 0.880 |
| BD P-tau217 | BD P-tau217/Aβ42 | 0.9996 | 0.914 to 1.091 | 0.966 |
| BD P-tau217 | BD P-tau217/P-tau217 | 1.1349 | 0.960 to 1.331 | 0.188 |
| BD P-tau217 | BD P-tau217/BD tau | 1.0435 | 0.905 to 1.205 | 0.659 |
| BD P-tau217/Aβ42 | BD P-tau217/P-tau217 | 1.1354 | 0.972 to 1.333 | 0.170 |
| BD P-tau217/Aβ42 | BD P-tau217/BD tau | 1.0439 | 0.929 to 1.182 | 0.577 |
| BD P-tau217/P-tau217 | BD P-tau217/BD tau | 0.9195 | 0.805 to 1.046 | 0.277 |
| **A2** | | | | |
| P-tau217 | P-tau217/Aβ42 | 0.9108 | 0.833 to 0.995 | 0.067 |
| P-tau217 | BD P-tau217 | 0.6778 | 0.601 to 0.764 | **<0.001** |
| P-tau217 | BD P-tau217/Aβ42 | 0.6173 | 0.528 to 0.719 | **<0.001** |
| P-tau217 | BD P-tau217/P-tau217 | 1.1818 | 0.948 to 1.469 | 0.185 |
| P-tau217 | BD P-tau217/BD tau | 0.7699 | 0.639 to 0.939 | **0.018** |
| P-tau217/Aβ42 | BD P-tau217 | 0.7442 | 0.644 to 0.859 | **<0.001** |
| P-tau217/Aβ42 | BD P-tau217/Aβ42 | 0.6778 | 0.601 to 0.764 | **<0.001** |
| P-tau217/Aβ42 | BD P-tau217/P-tau217 | 1.2976 | 1.049 to 1.604 | **0.030** |
| P-tau217/Aβ42 | BD P-tau217/BD tau | 0.8453 | 0.710 to 1.013 | 0.107 |
| BD P-tau217 | BD P-tau217/Aβ42 | 0.9108 | 0.833 to 0.995 | 0.067 |
| BD P-tau217 | BD P-tau217/P-tau217 | 1.7436 | 1.462 to 2.088 | **<0.001** |
| BD P-tau217 | BD P-tau217/BD tau | 1.1359 | 0.988 to 1.310 | 0.112 |
| BD P-tau217/Aβ42 | BD P-tau217/P-tau217 | 1.9145 | 1.603 to 2.291 | **<0.001** |
| BD P-tau217/Aβ42 | BD P-tau217/BD tau | 1.2472 | 1.100 to 1.414 | **<0.001** |
| BD P-tau217/P-tau217 | BD P-tau217/BD tau | 0.6515 | 0.570 to 0.740 | **<0.001** |
| **A3** | | | | |
| P-tau217 | P-tau217/Aβ42 | 0.8915 | 0.817 to 0.971 | **0.019** |
| P-tau217 | BD P-tau217 | 0.4927 | 0.449 to 0.543 | **<0.001** |
| P-tau217 | BD P-tau217/Aβ42 | 0.4392 | 0.387 to 0.501 | **<0.001** |
| P-tau217 | BD P-tau217/P-tau217 | 1.4690 | 1.214 to 1.773 | **<0.001** |
| P-tau217 | BD P-tau217/BD tau | 0.6535 | 0.553 to 0.769 | **<0.001** |
| P-tau217/Aβ42 | BD P-tau217 | 0.5526 | 0.488 to 0.631 | **<0.001** |
| P-tau217/Aβ42 | BD P-tau217/Aβ42 | 0.4927 | 0.449 to 0.543 | **<0.001** |
| P-tau217/Aβ42 | BD P-tau217/P-tau217 | 1.6477 | 1.373 to 1.972 | **<0.001** |
| P-tau217/Aβ42 | BD P-tau217/BD tau | 0.7330 | 0.632 to 0.850 | **<0.001** |
| BD P-tau217 | BD P-tau217/Aβ42 | 0.8915 | 0.817 to 0.971 | **0.019** |
| BD P-tau217 | BD P-tau217/P-tau217 | 2.9816 | 2.523 to 3.492 | **<0.001** |
| BD P-tau217 | BD P-tau217/BD tau | 1.3265 | 1.147 to 1.533 | **<0.001** |
| BD P-tau217/Aβ42 | BD P-tau217/P-tau217 | 3.3444 | 2.878 to 3.852 | **<0.001** |
| BD P-tau217/Aβ42 | BD P-tau217/BD tau | 1.4879 | 1.315 to 1.678 | **<0.001** |
| BD P-tau217/P-tau217 | BD P-tau217/BD tau | 0.4449 | 0.396 to 0.503 | **<0.001** |

Fold-changes are expressed relative to the A0 reference group for each Thal amyloid phase category. Biomarker log fold-changes were estimated from log-transformed models adjusted for age at death, sex, and the interval between last plasma sampling and autopsy. For this neuropathological outcome, analyses used the maximum complete-case sample with non-missing outcome data, non-missing covariates, and non-missing positive values for all six biomarkers, allowing direct pairwise comparison of biomarker fold-changes within this outcome. All biomarker models were fitted simultaneously on each resampled dataset so that pairwise differences are paired by construction; 95% confidence intervals and Benjamini-Hochberg FDR-adjusted bootstrap p-values are shown. Displayed fold-change ratios and 95% CIs are exponentiated paired differences in log fold-changes; values greater than 1 indicate a larger fold-change for biomarker 1 than biomarker 2. Bootstrap p-values are based on paired differences on the log fold-change scale.The effective analytic sample size for this analysis was n=268.

**Supplementary Table 7.** Observed adjusted fold-changes according to Braak stage.

| **Biomarker** | **III-IV** | **V-VI** |
| --- | --- | --- |
| P-tau217 | 1.20 (0.86, 1.67) | 2.67 (1.89, 3.75) |
| P-tau217/Aβ42 | 1.23 (0.87, 1.73) | 2.81 (1.97, 4.01) |
| BD P-tau217 | 1.23 (0.81, 1.86) | 4.74 (3.06, 7.32) |
| BD P-tau217/Aβ42 | 1.25 (0.81, 1.94) | 4.99 (3.17, 7.84) |
| BD P-tau217/P-tau217 | 1.02 (0.83, 1.26) | 1.78 (1.43, 2.21) |
| BD P-tau217/BD tau | 1.25 (0.89, 1.78) | 3.79 (2.64, 5.44) |

Fold-changes are expressed relative to the Braak I-II reference group for each Braak stage category. Estimates and 95% CIs were obtained from log-transformed linear models adjusted for age at death, sex, and the interval between last plasma sampling and autopsy, then exponentiated for presentation. Analyses used the common complete-case subset with non-missing outcome data, non-missing covariates, and non-missing positive values for all six plasma biomarkers. The effective analytic sample size was n=269.

**Supplementary Table 8.** Bootstrapped comparison of biomarker fold-change ratios according to Braak stage.

| **Biomarker 1** | **Biomarker 2** | **FC ratio** | **95% CI** | **FDR p** |
| --- | --- | --- | --- | --- |
| **III-IV** | | | | |
| P-tau217 | P-tau217/Aβ42 | 0.9761 | 0.858 to 1.116 | 0.926 |
| P-tau217 | BD P-tau217 | 0.9785 | 0.775 to 1.213 | 0.934 |
| P-tau217 | BD P-tau217/Aβ42 | 0.9551 | 0.716 to 1.289 | 0.926 |
| P-tau217 | BD P-tau217/P-tau217 | 1.1728 | 0.784 to 1.720 | 0.672 |
| P-tau217 | BD P-tau217/BD tau | 0.9550 | 0.700 to 1.291 | 0.926 |
| P-tau217/Aβ42 | BD P-tau217 | 1.0024 | 0.811 to 1.227 | 0.982 |
| P-tau217/Aβ42 | BD P-tau217/Aβ42 | 0.9785 | 0.775 to 1.213 | 0.934 |
| P-tau217/Aβ42 | BD P-tau217/P-tau217 | 1.2015 | 0.817 to 1.734 | 0.606 |
| P-tau217/Aβ42 | BD P-tau217/BD tau | 0.9783 | 0.742 to 1.284 | 0.934 |
| BD P-tau217 | BD P-tau217/Aβ42 | 0.9761 | 0.858 to 1.116 | 0.926 |
| BD P-tau217 | BD P-tau217/P-tau217 | 1.1987 | 0.904 to 1.572 | 0.371 |
| BD P-tau217 | BD P-tau217/BD tau | 0.9760 | 0.835 to 1.144 | 0.926 |
| BD P-tau217/Aβ42 | BD P-tau217/P-tau217 | 1.2280 | 0.912 to 1.656 | 0.363 |
| BD P-tau217/Aβ42 | BD P-tau217/BD tau | 0.9999 | 0.836 to 1.197 | 0.991 |
| BD P-tau217/P-tau217 | BD P-tau217/BD tau | 0.8142 | 0.655 to 1.011 | 0.130 |
| **V-VI** | | | | |
| P-tau217 | P-tau217/Aβ42 | 0.9493 | 0.829 to 1.091 | 0.672 |
| P-tau217 | BD P-tau217 | 0.5628 | 0.445 to 0.703 | **<0.001** |
| P-tau217 | BD P-tau217/Aβ42 | 0.5343 | 0.392 to 0.728 | **<0.001** |
| P-tau217 | BD P-tau217/P-tau217 | 1.5002 | 1.002 to 2.204 | 0.114 |
| P-tau217 | BD P-tau217/BD tau | 0.7035 | 0.515 to 0.955 | 0.063 |
| P-tau217/Aβ42 | BD P-tau217 | 0.5929 | 0.475 to 0.729 | **<0.001** |
| P-tau217/Aβ42 | BD P-tau217/Aβ42 | 0.5628 | 0.445 to 0.703 | **<0.001** |
| P-tau217/Aβ42 | BD P-tau217/P-tau217 | 1.5804 | 1.073 to 2.302 | 0.060 |
| P-tau217/Aβ42 | BD P-tau217/BD tau | 0.7411 | 0.558 to 0.970 | 0.076 |
| BD P-tau217 | BD P-tau217/Aβ42 | 0.9493 | 0.829 to 1.091 | 0.672 |
| BD P-tau217 | BD P-tau217/P-tau217 | 2.6654 | 1.971 to 3.559 | **<0.001** |
| BD P-tau217 | BD P-tau217/BD tau | 1.2499 | 1.052 to 1.478 | **0.040** |
| BD P-tau217/Aβ42 | BD P-tau217/P-tau217 | 2.8079 | 2.040 to 3.855 | **<0.001** |
| BD P-tau217/Aβ42 | BD P-tau217/BD tau | 1.3167 | 1.084 to 1.588 | **0.021** |
| BD P-tau217/P-tau217 | BD P-tau217/BD tau | 0.4689 | 0.372 to 0.589 | **<0.001** |

Fold-changes are expressed relative to the Braak I-II reference group for each Braak stage category. Biomarker log fold-changes were estimated from log-transformed models adjusted for age at death, sex, and the interval between last plasma sampling and autopsy. For this neuropathological outcome, analyses used the maximum complete-case sample with non-missing outcome data, non-missing covariates, and non-missing positive values for all six biomarkers, allowing direct pairwise comparison of biomarker fold-changes within this outcome. All biomarker models were fitted simultaneously on each resampled dataset so that pairwise differences are paired by construction; 95% confidence intervals and Benjamini-Hochberg FDR-adjusted bootstrap p-values are shown. Displayed fold-change ratios and 95% CIs are exponentiated paired differences in log fold-changes; values greater than 1 indicate a larger fold-change for biomarker 1 than biomarker 2. Bootstrap p-values are based on paired differences on the log fold-change scale.The effective analytic sample size for this analysis was n=269.

**Supplementary Table 9.** Model metrics for continuous plaque burden associations.

| **Biomarker** | **N** | **R² (base)** | **R² (linear, adj)** | **R² (GAM, adj)** | **R² (GAM, unadj)** | **ΔR² (GAM−base)** | **ΔR² (GAM−base) 95% CI** | **P-value** | **ΔAIC (linear−base)** | **ΔAIC (GAM−base)** | **ΔAIC (GAM−linear)** | **Best model** |
| --- | --- | --- | --- | --- | --- | --- | --- | --- | --- | --- | --- | --- |
| **Whole population** |  |  |  |  |  |  |  |  |  |  |  |  |
| P-tau217 | 269 | 0.018 | 0.295 | 0.450 | 0.433 | 0.432 | 0.336 to 0.527 | **<0.001** | -87 | -148 | -61 | GAM |
| P-tau217/Aβ42 | 269 | 0.018 | 0.313 | 0.555 | 0.547 | 0.537 | 0.447 to 0.620 | **<0.001** | -94 | -205 | -111 | GAM |
| BD P-tau217 | 269 | 0.018 | 0.282 | 0.590 | 0.578 | 0.572 | 0.484 to 0.654 | **<0.001** | -82 | -227 | -145 | GAM |
| BD P-tau217/Aβ42 | 269 | 0.018 | 0.278 | 0.646 | 0.640 | 0.628 | 0.535 to 0.706 | **<0.001** | -81 | -266 | -186 | GAM |
| BD P-tau217/P-tau217 | 269 | 0.018 | 0.366 | 0.486 | 0.468 | 0.468 | 0.378 to 0.569 | **<0.001** | -116 | -166 | -51 | GAM |
| BD P-tau217/BD tau | 269 | 0.018 | 0.345 | 0.660 | 0.659 | 0.642 | 0.553 to 0.716 | **<0.001** | -107 | -277 | -171 | GAM |
| **Non-AD** |  |  |  |  |  |  |  |  |  |  |  |  |
| P-tau217 | 111 | 0.056 | 0.070 | 0.082 | 0.015 | 0.026 | 0.000 to 0.161 | **<0.001** | 0 | 1 | 0 | Linear |
| P-tau217/Aβ42 | 111 | 0.056 | 0.065 | 0.114 | 0.069 | 0.059 | 0.001 to 0.322 | **<0.001** | 1 | -2 | -3 | GAM |
| BD P-tau217 | 111 | 0.056 | 0.089 | 0.270 | 0.191 | 0.214 | 0.050 to 0.397 | **<0.001** | -2 | -21 | -19 | GAM |
| BD P-tau217/Aβ42 | 111 | 0.056 | 0.110 | 0.239 | 0.202 | 0.183 | 0.054 to 0.468 | **<0.001** | -5 | -17 | -13 | GAM |
| BD P-tau217/P-tau217 | 111 | 0.056 | 0.134 | 0.150 | 0.127 | 0.095 | 0.013 to 0.352 | **<0.001** | -8 | -7 | 1 | Linear |
| BD P-tau217/BD tau | 111 | 0.056 | 0.219 | 0.298 | 0.268 | 0.242 | 0.025 to 0.460 | **<0.001** | -19 | -25 | -6 | GAM |
| **AD** |  |  |  |  |  |  |  |  |  |  |  |  |
| P-tau217 | 158 | 0.003 | 0.141 | 0.357 | 0.344 | 0.354 | 0.225 to 0.489 | **<0.001** | -22 | -61 | -40 | GAM |
| P-tau217/Aβ42 | 158 | 0.003 | 0.151 | 0.453 | 0.438 | 0.450 | 0.317 to 0.586 | **<0.001** | -23 | -87 | -63 | GAM |
| BD P-tau217 | 158 | 0.003 | 0.138 | 0.425 | 0.418 | 0.422 | 0.314 to 0.541 | **<0.001** | -21 | -79 | -58 | GAM |
| BD P-tau217/Aβ42 | 158 | 0.003 | 0.137 | 0.479 | 0.465 | 0.476 | 0.359 to 0.591 | **<0.001** | -21 | -95 | -74 | GAM |
| BD P-tau217/P-tau217 | 158 | 0.003 | 0.217 | 0.324 | 0.321 | 0.320 | 0.210 to 0.455 | **<0.001** | -36 | -54 | -18 | GAM |
| BD P-tau217/BD tau | 158 | 0.003 | 0.171 | 0.452 | 0.445 | 0.449 | 0.327 to 0.577 | **<0.001** | -27 | -87 | -59 | GAM |

This table summarizes covariate-only, adjusted linear, adjusted GAM, and unadjusted GAM model metrics for the association between plasma biomarkers and plaque burden. For each outcome-population combination, analyses were performed in the complete-case subset restricted to participants with non-missing values for the outcome, all six plasma biomarkers, age at death, sex, and the interval between last plasma collection and autopsy, with the effective analytic sample size shown in the N column. Adjusted models included age at death, sex, and the interval between last plasma collection and autopsy. Bootstrap percentile confidence intervals and FDR-corrected p-values are shown for ΔR² comparing the adjusted GAM with the covariate-only base model. R² was calculated from observed and fitted values using the same approach for the base, linear, and GAM models. GAM = generalized additive model; FDR = false discovery rate; R² = coefficient of determination.

**Supplementary Table 10.** Model metrics for continuous tangle burden associations.

| **Biomarker** | **N** | **R² (base)** | **R² (linear, adj)** | **R² (GAM, adj)** | **R² (GAM, unadj)** | **ΔR² (GAM−base)** | **ΔR² (GAM−base) 95% CI** | **P-value** | **ΔAIC (linear−base)** | **ΔAIC (GAM−base)** | **ΔAIC (GAM−linear)** | **Best model** |
| --- | --- | --- | --- | --- | --- | --- | --- | --- | --- | --- | --- | --- |
| **Whole population** |  |  |  |  |  |  |  |  |  |  |  |  |
| P-tau217 | 269 | 0.026 | 0.293 | 0.333 | 0.301 | 0.307 | 0.215 to 0.412 | **<0.001** | -84 | -96 | -11 | GAM |
| P-tau217/Aβ42 | 269 | 0.026 | 0.294 | 0.371 | 0.341 | 0.345 | 0.253 to 0.453 | **<0.001** | -85 | -111 | -26 | GAM |
| BD P-tau217 | 269 | 0.026 | 0.361 | 0.520 | 0.497 | 0.495 | 0.390 to 0.588 | **<0.001** | -112 | -184 | -72 | GAM |
| BD P-tau217/Aβ42 | 269 | 0.026 | 0.354 | 0.545 | 0.524 | 0.519 | 0.425 to 0.610 | **<0.001** | -109 | -197 | -89 | GAM |
| BD P-tau217/P-tau217 | 269 | 0.026 | 0.415 | 0.594 | 0.568 | 0.568 | 0.466 to 0.654 | **<0.001** | -135 | -228 | -92 | GAM |
| BD P-tau217/BD tau | 269 | 0.026 | 0.385 | 0.602 | 0.590 | 0.577 | 0.478 to 0.656 | **<0.001** | -122 | -233 | -112 | GAM |
| **Non-AD** |  |  |  |  |  |  |  |  |  |  |  |  |
| P-tau217 | 111 | 0.094 | 0.094 | 0.094 | 0.000 | 0.000 | 0.000 to 0.144 | **<0.001** | 2 | 2 | 0 | Linear |
| P-tau217/Aβ42 | 111 | 0.094 | 0.094 | 0.103 | 0.019 | 0.009 | 0.000 to 0.196 | **<0.001** | 2 | 2 | 0 | Linear |
| BD P-tau217 | 111 | 0.094 | 0.094 | 0.098 | 0.015 | 0.004 | 0.000 to 0.106 | **<0.001** | 2 | 2 | 0 | Linear |
| BD P-tau217/Aβ42 | 111 | 0.094 | 0.094 | 0.094 | 0.002 | 0.000 | 0.000 to 0.202 | **<0.001** | 2 | 2 | 0 | Linear |
| BD P-tau217/P-tau217 | 111 | 0.094 | 0.099 | 0.099 | 0.007 | 0.005 | 0.000 to 0.169 | **<0.001** | 1 | 1 | 0 | Linear |
| BD P-tau217/BD tau | 111 | 0.094 | 0.101 | 0.157 | 0.065 | 0.063 | 0.012 to 0.191 | **<0.001** | 1 | -3 | -4 | GAM |
| **AD** |  |  |  |  |  |  |  |  |  |  |  |  |
| P-tau217 | 158 | 0.075 | 0.296 | 0.373 | 0.268 | 0.298 | 0.199 to 0.430 | **<0.001** | -41 | -56 | -14 | GAM |
| P-tau217/Aβ42 | 158 | 0.075 | 0.276 | 0.415 | 0.311 | 0.340 | 0.228 to 0.466 | **<0.001** | -37 | -66 | -29 | GAM |
| BD P-tau217 | 158 | 0.075 | 0.348 | 0.587 | 0.505 | 0.512 | 0.392 to 0.614 | **<0.001** | -53 | -120 | -67 | GAM |
| BD P-tau217/Aβ42 | 158 | 0.075 | 0.320 | 0.600 | 0.539 | 0.525 | 0.410 to 0.633 | **<0.001** | -47 | -126 | -79 | GAM |
| BD P-tau217/P-tau217 | 158 | 0.075 | 0.426 | 0.681 | 0.671 | 0.606 | 0.483 to 0.713 | **<0.001** | -73 | -160 | -87 | GAM |
| BD P-tau217/BD tau | 158 | 0.075 | 0.347 | 0.651 | 0.633 | 0.576 | 0.451 to 0.679 | **<0.001** | -53 | -146 | -93 | GAM |

This table summarizes covariate-only, adjusted linear, adjusted GAM, and unadjusted GAM model metrics for the association between plasma biomarkers and tangle burden. For each outcome-population combination, analyses were performed in the complete-case subset restricted to participants with non-missing values for the outcome, all six plasma biomarkers, age at death, sex, and the interval between last plasma collection and autopsy, with the effective analytic sample size shown in the N column. Adjusted models included age at death, sex, and the interval between last plasma collection and autopsy. Bootstrap percentile confidence intervals and FDR-corrected p-values are shown for ΔR² comparing the adjusted GAM with the covariate-only base model. R² was calculated from observed and fitted values using the same approach for the base, linear, and GAM models. GAM = generalized additive model; FDR = false discovery rate; R² = coefficient of determination.

**Supplementary Table 11.** Bootstrapped pairwise comparison of variability explained by biomarker-pathology models for continuous plaque burden.

| **Biomarker 1** | **R^2^_1_** | **Biomarker 2** | **R^2^_2_** | **ΔR^2^ (1−2)** | **ΔR^2^ 95% CI** | **ΔR^2^ p (FDR)** | **ΔAIC (1−2)** |
| --- | --- | --- | --- | --- | --- | --- | --- |
| P-tau217 | 0.449 | P-tau217/Aβ42 | 0.553 | -0.105 | -0.161 to -0.046 | **0.001** | 56 |
| P-tau217 | 0.449 | BD P-tau217 | 0.588 | -0.140 | -0.203 to -0.078 | **<0.001** | 78 |
| P-tau217 | 0.449 | BD P-tau217/Aβ42 | 0.644 | -0.195 | -0.273 to -0.114 | **<0.001** | 117 |
| P-tau217 | 0.449 | BD P-tau217/P-tau217 | 0.485 | -0.037 | -0.164 to 0.075 | 0.492 | 18 |
| P-tau217 | 0.449 | BD P-tau217/BD tau | 0.658 | -0.210 | -0.312 to -0.103 | **<0.001** | 128 |
| P-tau217/Aβ42 | 0.553 | BD P-tau217 | 0.588 | -0.035 | -0.111 to 0.036 | 0.378 | 22 |
| P-tau217/Aβ42 | 0.553 | BD P-tau217/Aβ42 | 0.644 | -0.090 | -0.153 to -0.026 | **0.011** | 60 |
| P-tau217/Aβ42 | 0.553 | BD P-tau217/P-tau217 | 0.485 | 0.068 | -0.052 to 0.171 | 0.378 | -38 |
| P-tau217/Aβ42 | 0.553 | BD P-tau217/BD tau | 0.658 | -0.105 | -0.194 to -0.012 | **0.041** | 72 |
| BD P-tau217 | 0.588 | BD P-tau217/Aβ42 | 0.644 | -0.056 | -0.098 to -0.009 | **0.027** | 39 |
| BD P-tau217 | 0.588 | BD P-tau217/P-tau217 | 0.485 | 0.103 | 0.015 to 0.175 | **0.033** | -60 |
| BD P-tau217 | 0.588 | BD P-tau217/BD tau | 0.658 | -0.070 | -0.149 to 0.017 | 0.164 | 50 |
| BD P-tau217/Aβ42 | 0.644 | BD P-tau217/P-tau217 | 0.485 | 0.158 | 0.067 to 0.227 | **<0.001** | -99 |
| BD P-tau217/Aβ42 | 0.644 | BD P-tau217/BD tau | 0.658 | -0.014 | -0.093 to 0.065 | 0.774 | 11 |
| BD P-tau217/P-tau217 | 0.485 | BD P-tau217/BD tau | 0.658 | -0.173 | -0.245 to -0.078 | **<0.001** | 110 |

This table reports pairwise comparisons of adjusted generalized additive model (GAM) fit for the association between plasma biomarkers and continuous plaque burden in the whole population. Analyses were performed in the complete-case subset with non-missing values for all six plasma biomarker measures (n=269). GAMs were fit with a spline term for biomarker (k=5) and were adjusted for age at death, sex and interval between last plasma collection and autopsy. R² is the adjusted GAM R² from the full data; ΔR² is the difference in R² between biomarker 1 and biomarker 2; ΔAIC is AIC(biomarker 1) minus AIC(biomarker 2) from the full data. All six biomarker GAMs were fitted on a single resampled dataset per bootstrap replicate, so every pairwise ΔR² is paired by construction. Bootstrap percentile 95% confidence intervals and Benjamini-Hochberg FDR-adjusted bootstrap p-values are shown. R² was calculated from the observed and fitted values using the same approach for the base, linear, and GAM models. AIC = Akaike information criterion; CI = confidence interval; FDR = false discovery rate; GAM = generalized additive model.

**Supplementary Table 12.** Bootstrapped pairwise comparison of variability explained by biomarker-pathology models for continuous plaque burden stratified by neuropathological group.

| **Biomarker 1** | **R^2^_1_** | **Biomarker 2** | **R^2^_2_** | **ΔR^2^ (1−2)** | **ΔR^2^ 95% CI** | **ΔR^2^ p (FDR)** | **ΔAIC (1−2)** |
| --- | --- | --- | --- | --- | --- | --- | --- |
| **Non-AD** | | | | | | | |
| P-tau217 | 0.082 | P-tau217/Aβ42 | 0.114 | -0.032 | -0.243 to 0.056 | 0.675 | 3 |
| P-tau217 | 0.082 | BD P-tau217 | 0.270 | -0.188 | -0.321 to -0.011 | 0.372 | 21 |
| P-tau217 | 0.082 | BD P-tau217/Aβ42 | 0.239 | -0.157 | -0.412 to -0.001 | 0.372 | 18 |
| P-tau217 | 0.082 | BD P-tau217/P-tau217 | 0.150 | -0.068 | -0.318 to 0.096 | 0.675 | 8 |
| P-tau217 | 0.082 | BD P-tau217/BD tau | 0.298 | -0.216 | -0.423 to 0.060 | 0.660 | 26 |
| P-tau217/Aβ42 | 0.114 | BD P-tau217 | 0.270 | -0.155 | -0.305 to 0.105 | 0.675 | 19 |
| P-tau217/Aβ42 | 0.114 | BD P-tau217/Aβ42 | 0.239 | -0.124 | -0.351 to 0.094 | 0.675 | 16 |
| P-tau217/Aβ42 | 0.114 | BD P-tau217/P-tau217 | 0.150 | -0.036 | -0.303 to 0.263 | 0.819 | 5 |
| P-tau217/Aβ42 | 0.114 | BD P-tau217/BD tau | 0.298 | -0.184 | -0.400 to 0.195 | 0.675 | 23 |
| BD P-tau217 | 0.270 | BD P-tau217/Aβ42 | 0.239 | 0.031 | -0.214 to 0.140 | 0.987 | -3 |
| BD P-tau217 | 0.270 | BD P-tau217/P-tau217 | 0.150 | 0.119 | -0.159 to 0.277 | 0.675 | -14 |
| BD P-tau217 | 0.270 | BD P-tau217/BD tau | 0.298 | -0.028 | -0.264 to 0.207 | 0.987 | 4 |
| BD P-tau217/Aβ42 | 0.239 | BD P-tau217/P-tau217 | 0.150 | 0.089 | -0.137 to 0.318 | 0.675 | -10 |
| BD P-tau217/Aβ42 | 0.239 | BD P-tau217/BD tau | 0.298 | -0.059 | -0.237 to 0.225 | 0.987 | 8 |
| BD P-tau217/P-tau217 | 0.150 | BD P-tau217/BD tau | 0.298 | -0.148 | -0.290 to 0.094 | 0.675 | 18 |
| **AD** | | | | | | | |
| P-tau217 | 0.355 | P-tau217/Aβ42 | 0.454 | -0.099 | -0.180 to -0.011 | 0.100 | 26 |
| P-tau217 | 0.355 | BD P-tau217 | 0.422 | -0.067 | -0.154 to 0.016 | 0.231 | 17 |
| P-tau217 | 0.355 | BD P-tau217/Aβ42 | 0.477 | -0.122 | -0.221 to -0.013 | 0.100 | 33 |
| P-tau217 | 0.355 | BD P-tau217/P-tau217 | 0.324 | 0.031 | -0.116 to 0.164 | 0.800 | -7 |
| P-tau217 | 0.355 | BD P-tau217/BD tau | 0.450 | -0.094 | -0.216 to 0.032 | 0.232 | 25 |
| P-tau217/Aβ42 | 0.454 | BD P-tau217 | 0.422 | 0.032 | -0.082 to 0.139 | 0.765 | -9 |
| P-tau217/Aβ42 | 0.454 | BD P-tau217/Aβ42 | 0.477 | -0.023 | -0.105 to 0.064 | 0.765 | 7 |
| P-tau217/Aβ42 | 0.454 | BD P-tau217/P-tau217 | 0.324 | 0.130 | -0.022 to 0.266 | 0.231 | -33 |
| P-tau217/Aβ42 | 0.454 | BD P-tau217/BD tau | 0.450 | 0.004 | -0.116 to 0.119 | 0.971 | -1 |
| BD P-tau217 | 0.422 | BD P-tau217/Aβ42 | 0.477 | -0.055 | -0.108 to 0.013 | 0.231 | 16 |
| BD P-tau217 | 0.422 | BD P-tau217/P-tau217 | 0.324 | 0.099 | 0.009 to 0.178 | 0.100 | -24 |
| BD P-tau217 | 0.422 | BD P-tau217/BD tau | 0.450 | -0.027 | -0.113 to 0.068 | 0.765 | 8 |
| BD P-tau217/Aβ42 | 0.477 | BD P-tau217/P-tau217 | 0.324 | 0.153 | 0.052 to 0.232 | **0.030** | -40 |
| BD P-tau217/Aβ42 | 0.477 | BD P-tau217/BD tau | 0.450 | 0.027 | -0.056 to 0.109 | 0.765 | -8 |
| BD P-tau217/P-tau217 | 0.324 | BD P-tau217/BD tau | 0.450 | -0.126 | -0.215 to -0.023 | 0.100 | 32 |

This table reports pairwise comparisons of adjusted generalized additive model (GAM) fit for the association between plasma biomarkers and continuous plaque burden, fitted separately within Non-AD (Not AD and Low ADNC) and AD (Intermediate and High ADNC) participants. Analyses were performed in the complete-case subset with non-missing values for all six plasma biomarker measures (n=269). GAMs were fit with a spline term for biomarker (k=5) and were adjusted for age at death, sex and interval between last plasma collection and autopsy. R² is the adjusted GAM R² from the full data; ΔR² is the difference in R² between biomarker 1 and biomarker 2; ΔAIC is AIC(biomarker 1) minus AIC(biomarker 2) from the full data. All six biomarker GAMs were fitted on a single resampled dataset per bootstrap replicate, so every pairwise ΔR² is paired by construction. Bootstrap percentile 95% confidence intervals and Benjamini-Hochberg FDR-adjusted bootstrap p-values are shown. R² was calculated from the observed and fitted values using the same approach for the base, linear, and GAM models. AD = Alzheimer's disease; ADNC = Alzheimer's disease neuropathologic change; AIC = Akaike information criterion; CI = confidence interval; FDR = false discovery rate; GAM = generalized additive model.

**Supplementary Table 13.** Bootstrapped pairwise comparison of variability explained by biomarker-pathology models for continuous neurofibrillary tangle burden.

| **Biomarker 1** | **R^2^_1_** | **Biomarker 2** | **R^2^_2_** | **ΔR^2^ (1−2)** | **ΔR^2^ 95% CI** | **ΔR^2^ p (FDR)** | **ΔAIC (1−2)** |
| --- | --- | --- | --- | --- | --- | --- | --- |
| P-tau217 | 0.334 | P-tau217/Aβ42 | 0.376 | -0.042 | -0.096 to 0.004 | 0.097 | 17 |
| P-tau217 | 0.334 | BD P-tau217 | 0.522 | -0.188 | -0.234 to -0.130 | **<0.001** | 88 |
| P-tau217 | 0.334 | BD P-tau217/Aβ42 | 0.550 | -0.216 | -0.281 to -0.148 | **<0.001** | 103 |
| P-tau217 | 0.334 | BD P-tau217/P-tau217 | 0.595 | -0.261 | -0.342 to -0.161 | **<0.001** | 131 |
| P-tau217 | 0.334 | BD P-tau217/BD tau | 0.606 | -0.272 | -0.342 to -0.182 | **<0.001** | 139 |
| P-tau217/Aβ42 | 0.376 | BD P-tau217 | 0.522 | -0.146 | -0.201 to -0.072 | **<0.001** | 72 |
| P-tau217/Aβ42 | 0.376 | BD P-tau217/Aβ42 | 0.550 | -0.173 | -0.221 to -0.117 | **<0.001** | 87 |
| P-tau217/Aβ42 | 0.376 | BD P-tau217/P-tau217 | 0.595 | -0.219 | -0.296 to -0.120 | **<0.001** | 115 |
| P-tau217/Aβ42 | 0.376 | BD P-tau217/BD tau | 0.606 | -0.230 | -0.285 to -0.150 | **<0.001** | 123 |
| BD P-tau217 | 0.522 | BD P-tau217/Aβ42 | 0.550 | -0.027 | -0.079 to 0.009 | 0.157 | 15 |
| BD P-tau217 | 0.522 | BD P-tau217/P-tau217 | 0.595 | -0.072 | -0.134 to -0.010 | **0.033** | 43 |
| BD P-tau217 | 0.522 | BD P-tau217/BD tau | 0.606 | -0.084 | -0.140 to -0.027 | **0.001** | 51 |
| BD P-tau217/Aβ42 | 0.550 | BD P-tau217/P-tau217 | 0.595 | -0.045 | -0.097 to 0.015 | 0.178 | 28 |
| BD P-tau217/Aβ42 | 0.550 | BD P-tau217/BD tau | 0.606 | -0.057 | -0.088 to -0.011 | **0.015** | 36 |
| BD P-tau217/P-tau217 | 0.595 | BD P-tau217/BD tau | 0.606 | -0.012 | -0.056 to 0.042 | 0.730 | 8 |

This table reports pairwise comparisons of adjusted generalized additive model (GAM) fit for the association between plasma biomarkers and continuous neurofibrillary tangle burden in the whole population. Analyses were performed in the complete-case subset with non-missing values for all six plasma biomarker measures (n=269). GAMs were fit with a spline term for biomarker (k=5) and were adjusted for age at death, sex and interval between last plasma collection and autopsy. R² is the adjusted GAM R² from the full data; ΔR² is the difference in R² between biomarker 1 and biomarker 2; ΔAIC is AIC(biomarker 1) minus AIC(biomarker 2) from the full data. All six biomarker GAMs were fitted on a single resampled dataset per bootstrap replicate, so every pairwise ΔR² is paired by construction. Bootstrap percentile 95% confidence intervals and Benjamini-Hochberg FDR-adjusted bootstrap p-values are shown. R² was calculated from the observed and fitted values using the same approach for the base, linear, and GAM models. AIC = Akaike information criterion; CI = confidence interval; FDR = false discovery rate; GAM = generalized additive model.

**Supplementary Table 14.** Bootstrapped pairwise comparison of variability explained by biomarker-pathology models for continuous neurofibrillary tangle burden stratified by neuropathological group.

| **Biomarker 1** | **R^2^_1_** | **Biomarker 2** | **R^2^_2_** | **ΔR^2^ (1−2)** | **ΔR^2^ 95% CI** | **ΔR^2^ p (FDR)** | **ΔAIC (1−2)** |
| --- | --- | --- | --- | --- | --- | --- | --- |
| **Non-AD** | | | | | | | |
| P-tau217 | 0.094 | P-tau217/Aβ42 | 0.103 | -0.009 | -0.160 to 0.094 | 0.974 | 0 |
| P-tau217 | 0.094 | BD P-tau217 | 0.098 | -0.004 | -0.077 to 0.126 | 0.974 | 0 |
| P-tau217 | 0.094 | BD P-tau217/Aβ42 | 0.094 | -0.000 | -0.181 to 0.117 | 0.974 | 0 |
| P-tau217 | 0.094 | BD P-tau217/P-tau217 | 0.099 | -0.005 | -0.144 to 0.114 | 0.974 | 1 |
| P-tau217 | 0.094 | BD P-tau217/BD tau | 0.157 | -0.063 | -0.162 to 0.072 | 0.974 | 5 |
| P-tau217/Aβ42 | 0.103 | BD P-tau217 | 0.098 | 0.005 | -0.074 to 0.180 | 0.974 | 0 |
| P-tau217/Aβ42 | 0.103 | BD P-tau217/Aβ42 | 0.094 | 0.009 | -0.157 to 0.153 | 0.974 | 0 |
| P-tau217/Aβ42 | 0.103 | BD P-tau217/P-tau217 | 0.099 | 0.004 | -0.123 to 0.164 | 0.974 | 1 |
| P-tau217/Aβ42 | 0.103 | BD P-tau217/BD tau | 0.157 | -0.054 | -0.162 to 0.132 | 0.974 | 5 |
| BD P-tau217 | 0.098 | BD P-tau217/Aβ42 | 0.094 | 0.004 | -0.169 to 0.053 | 0.974 | 0 |
| BD P-tau217 | 0.098 | BD P-tau217/P-tau217 | 0.099 | -0.001 | -0.143 to 0.063 | 0.974 | 1 |
| BD P-tau217 | 0.098 | BD P-tau217/BD tau | 0.157 | -0.059 | -0.152 to 0.018 | 0.974 | 5 |
| BD P-tau217/Aβ42 | 0.094 | BD P-tau217/P-tau217 | 0.099 | -0.005 | -0.133 to 0.169 | 0.974 | 1 |
| BD P-tau217/Aβ42 | 0.094 | BD P-tau217/BD tau | 0.157 | -0.063 | -0.143 to 0.127 | 0.974 | 5 |
| BD P-tau217/P-tau217 | 0.099 | BD P-tau217/BD tau | 0.157 | -0.058 | -0.146 to 0.098 | 0.974 | 4 |
| **AD** | | | | | | | |
| P-tau217 | 0.373 | P-tau217/Aβ42 | 0.416 | -0.043 | -0.096 to 0.025 | 0.267 | 11 |
| P-tau217 | 0.373 | BD P-tau217 | 0.587 | -0.214 | -0.274 to -0.129 | **<0.001** | 65 |
| P-tau217 | 0.373 | BD P-tau217/Aβ42 | 0.603 | -0.230 | -0.302 to -0.138 | **<0.001** | 71 |
| P-tau217 | 0.373 | BD P-tau217/P-tau217 | 0.681 | -0.308 | -0.403 to -0.185 | **<0.001** | 104 |
| P-tau217 | 0.373 | BD P-tau217/BD tau | 0.654 | -0.281 | -0.359 to -0.169 | **<0.001** | 91 |
| P-tau217/Aβ42 | 0.416 | BD P-tau217 | 0.587 | -0.171 | -0.246 to -0.084 | **<0.001** | 54 |
| P-tau217/Aβ42 | 0.416 | BD P-tau217/Aβ42 | 0.603 | -0.187 | -0.253 to -0.118 | **<0.001** | 60 |
| P-tau217/Aβ42 | 0.416 | BD P-tau217/P-tau217 | 0.681 | -0.265 | -0.372 to -0.148 | **<0.001** | 93 |
| P-tau217/Aβ42 | 0.416 | BD P-tau217/BD tau | 0.654 | -0.238 | -0.320 to -0.137 | **<0.001** | 80 |
| BD P-tau217 | 0.587 | BD P-tau217/Aβ42 | 0.603 | -0.016 | -0.062 to 0.023 | 0.332 | 6 |
| BD P-tau217 | 0.587 | BD P-tau217/P-tau217 | 0.681 | -0.094 | -0.169 to -0.024 | **0.012** | 40 |
| BD P-tau217 | 0.587 | BD P-tau217/BD tau | 0.654 | -0.067 | -0.124 to -0.010 | **0.028** | 27 |
| BD P-tau217/Aβ42 | 0.603 | BD P-tau217/P-tau217 | 0.681 | -0.078 | -0.149 to -0.008 | **0.039** | 33 |
| BD P-tau217/Aβ42 | 0.603 | BD P-tau217/BD tau | 0.654 | -0.051 | -0.095 to 0.001 | 0.074 | 21 |
| BD P-tau217/P-tau217 | 0.681 | BD P-tau217/BD tau | 0.654 | 0.027 | -0.031 to 0.098 | 0.332 | -13 |

This table reports pairwise comparisons of adjusted generalized additive model (GAM) fit for the association between plasma biomarkers and continuous neurofibrillary tangle burden, fitted separately within Non-AD (Not AD and Low ADNC) and AD (Intermediate and High ADNC) participants. Analyses were performed in the complete-case subset with non-missing values for all six plasma biomarker measures (n=269). GAMs were fit with a spline term for biomarker (k=5) and were adjusted for age at death, sex and interval between last plasma collection and autopsy. R² is the adjusted GAM R² from the full data; ΔR² is the difference in R² between biomarker 1 and biomarker 2; ΔAIC is AIC(biomarker 1) minus AIC(biomarker 2) from the full data. All six biomarker GAMs were fitted on a single resampled dataset per bootstrap replicate, so every pairwise ΔR² is paired by construction. Bootstrap percentile 95% confidence intervals and Benjamini-Hochberg FDR-adjusted bootstrap p-values are shown. R² was calculated from the observed and fitted values using the same approach for the base, linear, and GAM models. AD = Alzheimer's disease; ADNC = Alzheimer's disease neuropathologic change; AIC = Akaike information criterion; CI = confidence interval; FDR = false discovery rate; GAM = generalized additive model.

**Supplementary Table 15.** Bootstrapped pairwise comparison of adjusted standardized linear associations and model R² between plasma biomarkers for continuous plaque burden.

|  |  | **Biomarker β-estimates** | | | | | **R²** | | | | |
| --- | --- | --- | --- | --- | --- | --- | --- | --- | --- | --- | --- |
| **Biomarker 1** | **Biomarker 2** | **β_1_** | **β_2_** | **Δβ (1−2)** | **Δβ 95% CI** | **Δβ p (FDR)** | **R^2^_1_** | **R^2^_2_** | **ΔR^2^ (1−2)** | **ΔR^2^ 95% CI** | **ΔR^2^ p (FDR)** |
| P-tau217 | P-tau217/Aβ42 | 3.188 | 3.289 | -0.101 | -0.458 to 0.168 | 0.812 | 0.293 | 0.309 | -0.016 | -0.088 to 0.031 | 0.865 |
| P-tau217 | BD P-tau217 | 3.188 | 3.123 | 0.065 | -0.431 to 0.479 | 0.975 | 0.293 | 0.281 | 0.012 | -0.074 to 0.079 | 0.932 |
| P-tau217 | BD P-tau217/Aβ42 | 3.188 | 3.126 | 0.062 | -0.657 to 0.512 | 0.984 | 0.293 | 0.276 | 0.017 | -0.122 to 0.092 | 0.932 |
| P-tau217 | BD P-tau217/P-tau217 | 3.188 | 3.614 | -0.426 | -1.065 to 0.181 | 0.505 | 0.293 | 0.366 | -0.072 | -0.191 to 0.033 | 0.526 |
| P-tau217 | BD P-tau217/BD tau | 3.188 | 3.487 | -0.299 | -1.177 to 0.413 | 0.812 | 0.293 | 0.342 | -0.049 | -0.213 to 0.069 | 0.865 |
| P-tau217/Aβ42 | BD P-tau217 | 3.289 | 3.123 | 0.166 | -0.456 to 0.732 | 0.941 | 0.309 | 0.281 | 0.028 | -0.076 to 0.128 | 0.865 |
| P-tau217/Aβ42 | BD P-tau217/Aβ42 | 3.289 | 3.126 | 0.163 | -0.588 to 0.698 | 0.947 | 0.309 | 0.276 | 0.033 | -0.106 to 0.123 | 0.865 |
| P-tau217/Aβ42 | BD P-tau217/P-tau217 | 3.289 | 3.614 | -0.325 | -1.024 to 0.381 | 0.812 | 0.309 | 0.366 | -0.056 | -0.184 to 0.071 | 0.865 |
| P-tau217/Aβ42 | BD P-tau217/BD tau | 3.289 | 3.487 | -0.198 | -1.123 to 0.589 | 0.941 | 0.309 | 0.342 | -0.033 | -0.206 to 0.107 | 0.865 |
| BD P-tau217 | BD P-tau217/Aβ42 | 3.123 | 3.126 | -0.003 | -0.309 to 0.146 | 0.947 | 0.281 | 0.276 | 0.005 | -0.060 to 0.037 | 0.932 |
| BD P-tau217 | BD P-tau217/P-tau217 | 3.123 | 3.614 | -0.491 | -0.854 to -0.098 | 0.188 | 0.281 | 0.366 | -0.085 | -0.155 to -0.014 | 0.218 |
| BD P-tau217 | BD P-tau217/BD tau | 3.123 | 3.487 | -0.364 | -0.895 to -0.018 | 0.188 | 0.281 | 0.342 | -0.062 | -0.167 to -0.001 | 0.218 |
| BD P-tau217/Aβ42 | BD P-tau217/P-tau217 | 3.126 | 3.614 | -0.488 | -0.854 to 0.025 | 0.257 | 0.276 | 0.366 | -0.090 | -0.169 to 0.008 | 0.317 |
| BD P-tau217/Aβ42 | BD P-tau217/BD tau | 3.126 | 3.487 | -0.361 | -0.884 to -0.020 | 0.188 | 0.276 | 0.342 | -0.067 | -0.180 to -0.002 | 0.218 |
| BD P-tau217/P-tau217 | BD P-tau217/BD tau | 3.614 | 3.487 | 0.127 | -0.445 to 0.474 | 0.947 | 0.366 | 0.342 | 0.023 | -0.091 to 0.083 | 0.932 |

This table reports pairwise comparisons of the standardized linear association between plasma biomarkers and continuous plaque burden in the whole population. For comparability, analyses were performed in the complete-case subset with non-missing values for all six plasma biomarker measures (n=269). Each adjusted linear model was adjusted for age at death, sex and interval between last plasma collection and autopsy. Because the biomarker was z-scored within each analysis cell, β is the standardized change in plaque burden per +1 SD increase in the biomarker. β and R² are from the full-data adjusted linear models; Δβ and ΔR² are the signed differences between biomarker 1 and biomarker 2. All six biomarker linear models were fitted on a single resampled dataset per bootstrap replicate, so every pairwise Δβ and ΔR² is paired by construction. Bootstrap percentile 95% confidence intervals and Benjamini-Hochberg FDR-adjusted bootstrap p-values are shown. R² was computed from observed versus fitted values. CI = confidence interval; FDR = false discovery rate; SD = standard deviation.

**Supplementary Table 16.** Bootstrapped pairwise comparison of adjusted standardized linear associations and model R² between plasma biomarkers for continuous plaque burden stratified by neuropathological group.

|  |  | **Biomarker β-estimates** | | | | | **R²** | | | | |
| --- | --- | --- | --- | --- | --- | --- | --- | --- | --- | --- | --- |
| **Biomarker 1** | **Biomarker 2** | **β_1_** | **β_2_** | **Δβ (1−2)** | **Δβ 95% CI** | **Δβ p (FDR)** | **R^2^_1_** | **R^2^_2_** | **ΔR^2^ (1−2)** | **ΔR^2^ 95% CI** | **ΔR^2^ p (FDR)** |
| **Non-AD** | | | | | | | | | | | |
| P-tau217 | P-tau217/Aβ42 | 0.282 | 0.230 | 0.052 | -0.437 to 0.315 | 0.873 | 0.070 | 0.065 | 0.005 | -0.077 to 0.044 | 0.965 |
| P-tau217 | BD P-tau217 | 0.282 | 0.440 | -0.158 | -0.720 to 0.197 | 0.624 | 0.070 | 0.089 | -0.019 | -0.133 to 0.038 | 0.601 |
| P-tau217 | BD P-tau217/Aβ42 | 0.282 | 0.568 | -0.286 | -1.089 to 0.248 | 0.624 | 0.070 | 0.110 | -0.040 | -0.260 to 0.039 | 0.601 |
| P-tau217 | BD P-tau217/P-tau217 | 0.282 | 0.682 | -0.399 | -1.229 to 0.494 | 0.624 | 0.070 | 0.134 | -0.065 | -0.211 to 0.085 | 0.601 |
| P-tau217 | BD P-tau217/BD tau | 0.282 | 0.956 | -0.674 | -1.527 to 0.187 | 0.624 | 0.070 | 0.219 | -0.149 | -0.361 to 0.036 | 0.601 |
| P-tau217/Aβ42 | BD P-tau217 | 0.230 | 0.440 | -0.210 | -0.788 to 0.384 | 0.628 | 0.065 | 0.089 | -0.024 | -0.135 to 0.080 | 0.697 |
| P-tau217/Aβ42 | BD P-tau217/Aβ42 | 0.230 | 0.568 | -0.338 | -0.843 to 0.097 | 0.624 | 0.065 | 0.110 | -0.045 | -0.217 to 0.019 | 0.601 |
| P-tau217/Aβ42 | BD P-tau217/P-tau217 | 0.230 | 0.682 | -0.451 | -1.254 to 0.542 | 0.624 | 0.065 | 0.134 | -0.069 | -0.211 to 0.103 | 0.601 |
| P-tau217/Aβ42 | BD P-tau217/BD tau | 0.230 | 0.956 | -0.726 | -1.531 to 0.203 | 0.624 | 0.065 | 0.219 | -0.154 | -0.358 to 0.041 | 0.601 |
| BD P-tau217 | BD P-tau217/Aβ42 | 0.440 | 0.568 | -0.128 | -0.755 to 0.359 | 0.831 | 0.089 | 0.110 | -0.021 | -0.192 to 0.066 | 0.821 |
| BD P-tau217 | BD P-tau217/P-tau217 | 0.440 | 0.682 | -0.242 | -0.800 to 0.505 | 0.701 | 0.089 | 0.134 | -0.046 | -0.169 to 0.103 | 0.697 |
| BD P-tau217 | BD P-tau217/BD tau | 0.440 | 0.956 | -0.516 | -1.152 to 0.216 | 0.624 | 0.089 | 0.219 | -0.130 | -0.323 to 0.043 | 0.601 |
| BD P-tau217/Aβ42 | BD P-tau217/P-tau217 | 0.568 | 0.682 | -0.114 | -0.730 to 0.720 | 0.873 | 0.110 | 0.134 | -0.025 | -0.160 to 0.203 | 0.915 |
| BD P-tau217/Aβ42 | BD P-tau217/BD tau | 0.568 | 0.956 | -0.389 | -1.004 to 0.376 | 0.624 | 0.110 | 0.219 | -0.109 | -0.291 to 0.110 | 0.601 |
| BD P-tau217/P-tau217 | BD P-tau217/BD tau | 0.682 | 0.956 | -0.275 | -0.746 to 0.282 | 0.624 | 0.134 | 0.219 | -0.084 | -0.239 to 0.060 | 0.601 |
| **AD** | | | | | | | | | | | |
| P-tau217 | P-tau217/Aβ42 | 1.261 | 1.310 | -0.049 | -0.242 to 0.088 | 0.821 | 0.140 | 0.148 | -0.008 | -0.058 to 0.021 | 0.997 |
| P-tau217 | BD P-tau217 | 1.261 | 1.266 | -0.005 | -0.359 to 0.294 | 0.958 | 0.140 | 0.137 | 0.003 | -0.073 to 0.064 | 0.999 |
| P-tau217 | BD P-tau217/Aβ42 | 1.261 | 1.286 | -0.025 | -0.461 to 0.308 | 0.948 | 0.140 | 0.135 | 0.005 | -0.101 to 0.071 | 0.999 |
| P-tau217 | BD P-tau217/P-tau217 | 1.261 | 1.608 | -0.347 | -0.875 to 0.116 | 0.623 | 0.140 | 0.216 | -0.076 | -0.207 to 0.029 | 0.562 |
| P-tau217 | BD P-tau217/BD tau | 1.261 | 1.416 | -0.155 | -0.771 to 0.296 | 0.821 | 0.140 | 0.169 | -0.029 | -0.180 to 0.063 | 0.997 |
| P-tau217/Aβ42 | BD P-tau217 | 1.310 | 1.266 | 0.044 | -0.373 to 0.431 | 0.948 | 0.148 | 0.137 | 0.011 | -0.078 to 0.096 | 0.999 |
| P-tau217/Aβ42 | BD P-tau217/Aβ42 | 1.310 | 1.286 | 0.024 | -0.454 to 0.411 | 0.958 | 0.148 | 0.135 | 0.013 | -0.094 to 0.095 | 0.999 |
| P-tau217/Aβ42 | BD P-tau217/P-tau217 | 1.310 | 1.608 | -0.297 | -0.829 to 0.219 | 0.643 | 0.148 | 0.216 | -0.068 | -0.200 to 0.054 | 0.639 |
| P-tau217/Aβ42 | BD P-tau217/BD tau | 1.310 | 1.416 | -0.106 | -0.747 to 0.406 | 0.945 | 0.148 | 0.169 | -0.021 | -0.178 to 0.091 | 0.999 |
| BD P-tau217 | BD P-tau217/Aβ42 | 1.266 | 1.286 | -0.020 | -0.177 to 0.063 | 0.821 | 0.137 | 0.135 | 0.003 | -0.043 to 0.025 | 0.999 |
| BD P-tau217 | BD P-tau217/P-tau217 | 1.266 | 1.608 | -0.342 | -0.642 to -0.092 | 0.066 | 0.137 | 0.216 | -0.079 | -0.162 to -0.019 | 0.102 |
| BD P-tau217 | BD P-tau217/BD tau | 1.266 | 1.416 | -0.150 | -0.519 to 0.051 | 0.623 | 0.137 | 0.169 | -0.032 | -0.127 to 0.010 | 0.562 |
| BD P-tau217/Aβ42 | BD P-tau217/P-tau217 | 1.286 | 1.608 | -0.322 | -0.585 to -0.062 | 0.114 | 0.135 | 0.216 | -0.081 | -0.160 to -0.017 | 0.117 |
| BD P-tau217/Aβ42 | BD P-tau217/BD tau | 1.286 | 1.416 | -0.130 | -0.454 to 0.048 | 0.623 | 0.135 | 0.169 | -0.035 | -0.130 to 0.007 | 0.562 |
| BD P-tau217/P-tau217 | BD P-tau217/BD tau | 1.608 | 1.416 | 0.192 | -0.128 to 0.381 | 0.643 | 0.216 | 0.169 | 0.047 | -0.035 to 0.088 | 0.639 |

This table reports pairwise comparisons of the standardized linear association between plasma biomarkers and continuous plaque burden fitted separately within Non-AD (Not AD and Low ADNC) and AD (Intermediate and High ADNC) participants. For comparability, analyses were performed in the complete-case subset with non-missing values for all six plasma biomarker measures (n=269). Each adjusted linear model was adjusted for age at death, sex and interval between last plasma collection and autopsy. Because the biomarker was z-scored within each analysis cell, β is the standardized change in plaque burden per +1 SD increase in the biomarker. β and R² are from the full-data adjusted linear models; Δβ and ΔR² are the signed differences between biomarker 1 and biomarker 2. All six biomarker linear models were fitted on a single resampled dataset per bootstrap replicate, so every pairwise Δβ and ΔR² is paired by construction. Bootstrap percentile 95% confidence intervals and Benjamini-Hochberg FDR-adjusted bootstrap p-values are shown. R² was computed from observed versus fitted values. AD = Alzheimer's disease; ADNC = Alzheimer's disease neuropathologic change; CI = confidence interval; FDR = false discovery rate; SD = standard deviation.

**Supplementary Table 17.** Bootstrapped pairwise comparison of adjusted standardized linear associations and model R² between plasma biomarkers for continuous tangle burden.

|  |  | **Biomarker β-estimates** | | | | | **R²** | | | | |
| --- | --- | --- | --- | --- | --- | --- | --- | --- | --- | --- | --- |
| **Biomarker 1** | **Biomarker 2** | **β_1_** | **β_2_** | **Δβ (1−2)** | **Δβ 95% CI** | **Δβ p (FDR)** | **R^2^_1_** | **R^2^_2_** | **ΔR^2^ (1−2)** | **ΔR^2^ 95% CI** | **ΔR^2^ p (FDR)** |
| P-tau217 | P-tau217/Aβ42 | 1.782 | 1.797 | -0.015 | -0.208 to 0.133 | 0.819 | 0.294 | 0.297 | -0.003 | -0.069 to 0.038 | 0.901 |
| P-tau217 | BD P-tau217 | 1.782 | 1.999 | -0.217 | -0.705 to 0.205 | 0.677 | 0.294 | 0.362 | -0.068 | -0.229 to 0.057 | 0.671 |
| P-tau217 | BD P-tau217/Aβ42 | 1.782 | 2.002 | -0.220 | -0.809 to 0.228 | 0.677 | 0.294 | 0.356 | -0.062 | -0.277 to 0.070 | 0.671 |
| P-tau217 | BD P-tau217/P-tau217 | 1.782 | 2.167 | -0.385 | -0.913 to 0.117 | 0.677 | 0.294 | 0.416 | -0.122 | -0.298 to 0.036 | 0.671 |
| P-tau217 | BD P-tau217/BD tau | 1.782 | 2.081 | -0.299 | -1.046 to 0.315 | 0.677 | 0.294 | 0.386 | -0.092 | -0.357 to 0.088 | 0.671 |
| P-tau217/Aβ42 | BD P-tau217 | 1.797 | 1.999 | -0.202 | -0.754 to 0.283 | 0.677 | 0.297 | 0.362 | -0.065 | -0.244 to 0.082 | 0.671 |
| P-tau217/Aβ42 | BD P-tau217/Aβ42 | 1.797 | 2.002 | -0.205 | -0.836 to 0.290 | 0.677 | 0.297 | 0.356 | -0.059 | -0.283 to 0.087 | 0.671 |
| P-tau217/Aβ42 | BD P-tau217/P-tau217 | 1.797 | 2.167 | -0.370 | -0.948 to 0.171 | 0.677 | 0.297 | 0.416 | -0.119 | -0.307 to 0.052 | 0.671 |
| P-tau217/Aβ42 | BD P-tau217/BD tau | 1.797 | 2.081 | -0.284 | -1.067 to 0.365 | 0.677 | 0.297 | 0.386 | -0.089 | -0.363 to 0.106 | 0.671 |
| BD P-tau217 | BD P-tau217/Aβ42 | 1.999 | 2.002 | -0.003 | -0.188 to 0.073 | 0.819 | 0.362 | 0.356 | 0.006 | -0.074 to 0.040 | 0.901 |
| BD P-tau217 | BD P-tau217/P-tau217 | 1.999 | 2.167 | -0.168 | -0.374 to 0.061 | 0.677 | 0.362 | 0.416 | -0.054 | -0.126 to 0.023 | 0.671 |
| BD P-tau217 | BD P-tau217/BD tau | 1.999 | 2.081 | -0.082 | -0.437 to 0.158 | 0.692 | 0.362 | 0.386 | -0.024 | -0.167 to 0.045 | 0.671 |
| BD P-tau217/Aβ42 | BD P-tau217/P-tau217 | 2.002 | 2.167 | -0.165 | -0.359 to 0.131 | 0.677 | 0.356 | 0.416 | -0.060 | -0.137 to 0.057 | 0.671 |
| BD P-tau217/Aβ42 | BD P-tau217/BD tau | 2.002 | 2.081 | -0.079 | -0.405 to 0.123 | 0.699 | 0.356 | 0.386 | -0.031 | -0.176 to 0.033 | 0.671 |
| BD P-tau217/P-tau217 | BD P-tau217/BD tau | 2.167 | 2.081 | 0.086 | -0.273 to 0.312 | 0.824 | 0.416 | 0.386 | 0.029 | -0.114 to 0.093 | 0.901 |

This table reports pairwise comparisons of the standardized linear association between plasma biomarkers and continuous tangle burden in the whole population. For comparability, analyses were performed in the complete-case subset with non-missing values for all six plasma biomarker measures (n=269). Each adjusted linear model was adjusted for age at death, sex and interval between last plasma collection and autopsy. Because the biomarker was z-scored within each analysis cell, β is the standardized change in tangle burden per +1 SD increase in the biomarker. β and R² are from the full-data adjusted linear models; Δβ and ΔR² are the signed differences between biomarker 1 and biomarker 2. All six biomarker linear models were fitted on a single resampled dataset per bootstrap replicate, so every pairwise Δβ and ΔR² is paired by construction. Bootstrap percentile 95% confidence intervals and Benjamini-Hochberg FDR-adjusted bootstrap p-values are shown. R² was computed from observed versus fitted values. CI = confidence interval; FDR = false discovery rate; SD = standard deviation.

**Supplementary Table 18.** Bootstrapped pairwise comparison of adjusted standardized linear associations and model R² between plasma biomarkers for continuous tangle burden stratified by neuropathological group.

|  |  | **Biomarker β-estimates** | | | | | **R²** | | | | |
| --- | --- | --- | --- | --- | --- | --- | --- | --- | --- | --- | --- |
| **Biomarker 1** | **Biomarker 2** | **β_1_** | **β_2_** | **Δβ (1−2)** | **Δβ 95% CI** | **Δβ p (FDR)** | **R^2^_1_** | **R^2^_2_** | **ΔR^2^ (1−2)** | **ΔR^2^ 95% CI** | **ΔR^2^ p (FDR)** |
| **Non-AD** | | | | | | | | | | | |
| P-tau217 | P-tau217/Aβ42 | -0.016 | -0.015 | -0.002 | -0.201 to 0.301 | 0.962 | 0.094 | 0.094 | 0.000 | -0.033 to 0.020 | 0.982 |
| P-tau217 | BD P-tau217 | -0.016 | -0.031 | 0.015 | -0.393 to 0.341 | 0.962 | 0.094 | 0.094 | -0.000 | -0.023 to 0.028 | 0.982 |
| P-tau217 | BD P-tau217/Aβ42 | -0.016 | -0.024 | 0.008 | -0.421 to 0.475 | 0.962 | 0.094 | 0.094 | -0.000 | -0.029 to 0.030 | 0.982 |
| P-tau217 | BD P-tau217/P-tau217 | -0.016 | 0.169 | -0.185 | -0.897 to 0.528 | 0.962 | 0.094 | 0.099 | -0.005 | -0.059 to 0.026 | 0.982 |
| P-tau217 | BD P-tau217/BD tau | -0.016 | 0.197 | -0.214 | -0.886 to 0.373 | 0.962 | 0.094 | 0.101 | -0.007 | -0.098 to 0.029 | 0.982 |
| P-tau217/Aβ42 | BD P-tau217 | -0.015 | -0.031 | 0.016 | -0.537 to 0.384 | 0.962 | 0.094 | 0.094 | -0.000 | -0.027 to 0.047 | 0.982 |
| P-tau217/Aβ42 | BD P-tau217/Aβ42 | -0.015 | -0.024 | 0.009 | -0.409 to 0.389 | 0.962 | 0.094 | 0.094 | -0.000 | -0.024 to 0.039 | 0.982 |
| P-tau217/Aβ42 | BD P-tau217/P-tau217 | -0.015 | 0.169 | -0.183 | -0.970 to 0.502 | 0.962 | 0.094 | 0.099 | -0.005 | -0.059 to 0.034 | 0.982 |
| P-tau217/Aβ42 | BD P-tau217/BD tau | -0.015 | 0.197 | -0.212 | -0.900 to 0.296 | 0.962 | 0.094 | 0.101 | -0.007 | -0.096 to 0.043 | 0.982 |
| BD P-tau217 | BD P-tau217/Aβ42 | -0.031 | -0.024 | -0.007 | -0.217 to 0.340 | 0.962 | 0.094 | 0.094 | 0.000 | -0.026 to 0.021 | 0.982 |
| BD P-tau217 | BD P-tau217/P-tau217 | -0.031 | 0.169 | -0.199 | -0.607 to 0.280 | 0.962 | 0.094 | 0.099 | -0.005 | -0.061 to 0.019 | 0.982 |
| BD P-tau217 | BD P-tau217/BD tau | -0.031 | 0.197 | -0.228 | -0.727 to 0.211 | 0.962 | 0.094 | 0.101 | -0.007 | -0.095 to 0.016 | 0.982 |
| BD P-tau217/Aβ42 | BD P-tau217/P-tau217 | -0.024 | 0.169 | -0.193 | -0.651 to 0.221 | 0.962 | 0.094 | 0.099 | -0.005 | -0.061 to 0.030 | 0.982 |
| BD P-tau217/Aβ42 | BD P-tau217/BD tau | -0.024 | 0.197 | -0.221 | -0.687 to 0.109 | 0.962 | 0.094 | 0.101 | -0.007 | -0.093 to 0.030 | 0.982 |
| BD P-tau217/P-tau217 | BD P-tau217/BD tau | 0.169 | 0.197 | -0.029 | -0.507 to 0.373 | 0.962 | 0.099 | 0.101 | -0.002 | -0.075 to 0.037 | 0.982 |
| **AD** | | | | | | | | | | | |
| P-tau217 | P-tau217/Aβ42 | 1.633 | 1.582 | 0.051 | -0.203 to 0.219 | 0.867 | 0.295 | 0.276 | 0.018 | -0.066 to 0.055 | 0.761 |
| P-tau217 | BD P-tau217 | 1.633 | 1.839 | -0.206 | -0.860 to 0.353 | 0.829 | 0.295 | 0.346 | -0.052 | -0.254 to 0.088 | 0.761 |
| P-tau217 | BD P-tau217/Aβ42 | 1.633 | 1.795 | -0.162 | -0.955 to 0.429 | 0.829 | 0.295 | 0.320 | -0.026 | -0.306 to 0.111 | 0.761 |
| P-tau217 | BD P-tau217/P-tau217 | 1.633 | 2.104 | -0.471 | -1.266 to 0.247 | 0.829 | 0.295 | 0.425 | -0.130 | -0.380 to 0.066 | 0.761 |
| P-tau217 | BD P-tau217/BD tau | 1.633 | 1.848 | -0.215 | -1.259 to 0.567 | 0.829 | 0.295 | 0.346 | -0.051 | -0.388 to 0.138 | 0.761 |
| P-tau217/Aβ42 | BD P-tau217 | 1.582 | 1.839 | -0.257 | -0.985 to 0.406 | 0.829 | 0.276 | 0.346 | -0.070 | -0.282 to 0.102 | 0.761 |
| P-tau217/Aβ42 | BD P-tau217/Aβ42 | 1.582 | 1.795 | -0.213 | -1.067 to 0.448 | 0.829 | 0.276 | 0.320 | -0.044 | -0.320 to 0.115 | 0.761 |
| P-tau217/Aβ42 | BD P-tau217/P-tau217 | 1.582 | 2.104 | -0.522 | -1.355 to 0.278 | 0.829 | 0.276 | 0.425 | -0.148 | -0.400 to 0.076 | 0.761 |
| P-tau217/Aβ42 | BD P-tau217/BD tau | 1.582 | 1.848 | -0.266 | -1.369 to 0.587 | 0.829 | 0.276 | 0.346 | -0.069 | -0.410 to 0.147 | 0.761 |
| BD P-tau217 | BD P-tau217/Aβ42 | 1.839 | 1.795 | 0.044 | -0.200 to 0.145 | 0.867 | 0.346 | 0.320 | 0.026 | -0.074 to 0.065 | 0.761 |
| BD P-tau217 | BD P-tau217/P-tau217 | 1.839 | 2.104 | -0.265 | -0.593 to 0.020 | 0.552 | 0.346 | 0.425 | -0.078 | -0.192 to 0.007 | 0.627 |
| BD P-tau217 | BD P-tau217/BD tau | 1.839 | 1.848 | -0.009 | -0.550 to 0.287 | 0.901 | 0.346 | 0.346 | 0.001 | -0.180 to 0.071 | 0.914 |
| BD P-tau217/Aβ42 | BD P-tau217/P-tau217 | 1.795 | 2.104 | -0.310 | -0.613 to 0.027 | 0.552 | 0.320 | 0.425 | -0.105 | -0.230 to 0.011 | 0.627 |
| BD P-tau217/Aβ42 | BD P-tau217/BD tau | 1.795 | 1.848 | -0.053 | -0.556 to 0.199 | 0.867 | 0.320 | 0.346 | -0.026 | -0.216 to 0.049 | 0.761 |
| BD P-tau217/P-tau217 | BD P-tau217/BD tau | 2.104 | 1.848 | 0.257 | -0.154 to 0.454 | 0.829 | 0.425 | 0.346 | 0.079 | -0.054 to 0.120 | 0.761 |

This table reports pairwise comparisons of the standardized linear association between plasma biomarkers and continuous tangle burden fitted separately within Non-AD (Not AD and Low ADNC) and AD (Intermediate and High ADNC) participants. For comparability, analyses were performed in the complete-case subset with non-missing values for all six plasma biomarker measures (n=269). Each adjusted linear model was adjusted for age at death, sex and interval between last plasma collection and autopsy. Because the biomarker was z-scored within each analysis cell, β is the standardized change in tangle burden per +1 SD increase in the biomarker. β and R² are from the full-data adjusted linear models; Δβ and ΔR² are the signed differences between biomarker 1 and biomarker 2. All six biomarker linear models were fitted on a single resampled dataset per bootstrap replicate, so every pairwise Δβ and ΔR² is paired by construction. Bootstrap percentile 95% confidence intervals and Benjamini-Hochberg FDR-adjusted bootstrap p-values are shown. R² was computed from observed versus fitted values. AD = Alzheimer's disease; ADNC = Alzheimer's disease neuropathologic change; CI = confidence interval; FDR = false discovery rate; SD = standard deviation.

**Supplementary Table 19.** Pairwise DeLong comparisons of plasma biomarker AUCs for classification of NIA-AA neuropathological change.

| **Biomarker 1** | **AUC_1_** | **Biomarker 2** | **AUC_2_** | **ΔAUC (1−2)** | **Raw p** | **p (FDR)** |
| --- | --- | --- | --- | --- | --- | --- |
| **NIA-AA: Not/Low vs Int/High** | | | | | | |
| P-tau217 | 0.824 | P-tau217/Aβ42 | 0.856 | -0.032 | 0.028 | 0.061 |
| P-tau217 | 0.824 | BD P-tau217 | 0.861 | -0.037 | 0.024 | 0.061 |
| P-tau217 | 0.824 | BD P-tau217/Aβ42 | 0.874 | -0.050 | 0.010 | **0.039** |
| P-tau217 | 0.824 | BD P-tau217/P-tau217 | 0.811 | 0.013 | 0.690 | 0.739 |
| P-tau217 | 0.824 | BD P-tau217/BD tau | 0.892 | -0.068 | 0.010 | **0.039** |
| P-tau217/Aβ42 | 0.856 | BD P-tau217 | 0.861 | -0.005 | 0.786 | 0.786 |
| P-tau217/Aβ42 | 0.856 | BD P-tau217/Aβ42 | 0.874 | -0.018 | 0.209 | 0.261 |
| P-tau217/Aβ42 | 0.856 | BD P-tau217/P-tau217 | 0.811 | 0.045 | 0.163 | 0.222 |
| P-tau217/Aβ42 | 0.856 | BD P-tau217/BD tau | 0.892 | -0.036 | 0.108 | 0.180 |
| BD P-tau217 | 0.861 | BD P-tau217/Aβ42 | 0.874 | -0.013 | 0.152 | 0.222 |
| BD P-tau217 | 0.861 | BD P-tau217/P-tau217 | 0.811 | 0.050 | 0.018 | 0.053 |
| BD P-tau217 | 0.861 | BD P-tau217/BD tau | 0.892 | -0.031 | 0.101 | 0.180 |
| BD P-tau217/Aβ42 | 0.874 | BD P-tau217/P-tau217 | 0.811 | 0.063 | 0.003 | **0.026** |
| BD P-tau217/Aβ42 | 0.874 | BD P-tau217/BD tau | 0.892 | -0.018 | 0.290 | 0.334 |
| BD P-tau217/P-tau217 | 0.811 | BD P-tau217/BD tau | 0.892 | -0.081 | <0.001 | **0.002** |
| **NIA-AA: Not vs High** | | | | | | |
| P-tau217 | 0.956 | P-tau217/Aβ42 | 0.980 | -0.024 | 0.049 | 0.229 |
| P-tau217 | 0.956 | BD P-tau217 | 0.990 | -0.034 | 0.018 | 0.132 |
| P-tau217 | 0.956 | BD P-tau217/Aβ42 | 0.993 | -0.037 | 0.015 | 0.132 |
| P-tau217 | 0.956 | BD P-tau217/P-tau217 | 0.972 | -0.016 | 0.470 | 0.628 |
| P-tau217 | 0.956 | BD P-tau217/BD tau | 0.989 | -0.033 | 0.061 | 0.229 |
| P-tau217/Aβ42 | 0.980 | BD P-tau217 | 0.990 | -0.010 | 0.295 | 0.449 |
| P-tau217/Aβ42 | 0.980 | BD P-tau217/Aβ42 | 0.993 | -0.012 | 0.182 | 0.390 |
| P-tau217/Aβ42 | 0.980 | BD P-tau217/P-tau217 | 0.972 | 0.009 | 0.609 | 0.687 |
| P-tau217/Aβ42 | 0.980 | BD P-tau217/BD tau | 0.989 | -0.008 | 0.502 | 0.628 |
| BD P-tau217 | 0.990 | BD P-tau217/Aβ42 | 0.993 | -0.002 | 0.274 | 0.449 |
| BD P-tau217 | 0.990 | BD P-tau217/P-tau217 | 0.972 | 0.019 | 0.169 | 0.390 |
| BD P-tau217 | 0.990 | BD P-tau217/BD tau | 0.989 | 0.002 | 0.859 | 0.859 |
| BD P-tau217/Aβ42 | 0.993 | BD P-tau217/P-tau217 | 0.972 | 0.021 | 0.124 | 0.372 |
| BD P-tau217/Aβ42 | 0.993 | BD P-tau217/BD tau | 0.989 | 0.004 | 0.641 | 0.687 |
| BD P-tau217/P-tau217 | 0.972 | BD P-tau217/BD tau | 0.989 | -0.017 | 0.299 | 0.449 |

Pairwise DeLong tests comparing ROC curves for plasma biomarker classification of NIA-AA neuropathological change. Comparisons are shown for the two binary contrasts used in the ROC analyses: Not AD/Low ADNC versus Intermediate/High ADNC and Not AD versus High ADNC. AUC1 and AUC2 correspond to the AUC estimates for biomarker 1 and biomarker 2, respectively. Delta AUC is calculated as AUC1 minus AUC2, such that positive values indicate a higher AUC for biomarker 1. Raw p-values are from paired DeLong tests. FDR p-values were calculated using the Benjamini-Hochberg method within each binary contrast. Bold FDR p-values indicate p < 0.05. For comparability, analyses were performed in the complete-case subset with non-missing values for the six plasma biomarker measures (n=269). ADNC = Alzheimer's disease neuropathologic change; AUC = area under the curve; FDR = false discovery rate; NIA-AA = National Institute on Aging-Alzheimer's Association; ROC = receiver operating characteristic.

**Supplementary Table 20.** Pairwise DeLong comparisons of plasma biomarker AUCs for classification of Thal amyloid phase categories.

| **Biomarker 1** | **AUC_1_** | **Biomarker 2** | **AUC_2_** | **ΔAUC (1−2)** | **Raw p** | **p (FDR)** |
| --- | --- | --- | --- | --- | --- | --- |
| **Thal: A0/A1 vs A2/A3** | | | | | | |
| P-tau217 | 0.841 | P-tau217/Aβ42 | 0.878 | -0.037 | 0.005 | **0.015** |
| P-tau217 | 0.841 | BD P-tau217 | 0.908 | -0.067 | <0.001 | **<0.001** |
| P-tau217 | 0.841 | BD P-tau217/Aβ42 | 0.924 | -0.083 | <0.001 | **<0.001** |
| P-tau217 | 0.841 | BD P-tau217/P-tau217 | 0.876 | -0.035 | 0.242 | 0.302 |
| P-tau217 | 0.841 | BD P-tau217/BD tau | 0.923 | -0.082 | 0.001 | **0.004** |
| P-tau217/Aβ42 | 0.878 | BD P-tau217 | 0.908 | -0.030 | 0.069 | 0.103 |
| P-tau217/Aβ42 | 0.878 | BD P-tau217/Aβ42 | 0.924 | -0.046 | <0.001 | **0.004** |
| P-tau217/Aβ42 | 0.878 | BD P-tau217/P-tau217 | 0.876 | 0.002 | 0.944 | 0.944 |
| P-tau217/Aβ42 | 0.878 | BD P-tau217/BD tau | 0.923 | -0.045 | 0.038 | 0.071 |
| BD P-tau217 | 0.908 | BD P-tau217/Aβ42 | 0.924 | -0.016 | 0.063 | 0.103 |
| BD P-tau217 | 0.908 | BD P-tau217/P-tau217 | 0.876 | 0.032 | 0.086 | 0.117 |
| BD P-tau217 | 0.908 | BD P-tau217/BD tau | 0.923 | -0.014 | 0.402 | 0.464 |
| BD P-tau217/Aβ42 | 0.924 | BD P-tau217/P-tau217 | 0.876 | 0.048 | 0.013 | **0.028** |
| BD P-tau217/Aβ42 | 0.924 | BD P-tau217/BD tau | 0.923 | 0.001 | 0.930 | 0.944 |
| BD P-tau217/P-tau217 | 0.876 | BD P-tau217/BD tau | 0.923 | -0.047 | 0.013 | **0.028** |
| **Thal: A0 vs A3** | | | | | | |
| P-tau217 | 0.945 | P-tau217/Aβ42 | 0.979 | -0.034 | 0.022 | 0.112 |
| P-tau217 | 0.945 | BD P-tau217 | 0.989 | -0.044 | 0.004 | **0.043** |
| P-tau217 | 0.945 | BD P-tau217/Aβ42 | 0.993 | -0.048 | 0.006 | **0.043** |
| P-tau217 | 0.945 | BD P-tau217/P-tau217 | 0.963 | -0.018 | 0.478 | 0.552 |
| P-tau217 | 0.945 | BD P-tau217/BD tau | 0.981 | -0.037 | 0.083 | 0.227 |
| P-tau217/Aβ42 | 0.979 | BD P-tau217 | 0.989 | -0.010 | 0.370 | 0.505 |
| P-tau217/Aβ42 | 0.979 | BD P-tau217/Aβ42 | 0.993 | -0.014 | 0.200 | 0.398 |
| P-tau217/Aβ42 | 0.979 | BD P-tau217/P-tau217 | 0.963 | 0.016 | 0.411 | 0.513 |
| P-tau217/Aβ42 | 0.979 | BD P-tau217/BD tau | 0.981 | -0.002 | 0.892 | 0.892 |
| BD P-tau217 | 0.989 | BD P-tau217/Aβ42 | 0.993 | -0.004 | 0.212 | 0.398 |
| BD P-tau217 | 0.989 | BD P-tau217/P-tau217 | 0.963 | 0.026 | 0.091 | 0.227 |
| BD P-tau217 | 0.989 | BD P-tau217/BD tau | 0.981 | 0.008 | 0.547 | 0.586 |
| BD P-tau217/Aβ42 | 0.993 | BD P-tau217/P-tau217 | 0.963 | 0.030 | 0.039 | 0.145 |
| BD P-tau217/Aβ42 | 0.993 | BD P-tau217/BD tau | 0.981 | 0.011 | 0.345 | 0.505 |
| BD P-tau217/P-tau217 | 0.963 | BD P-tau217/BD tau | 0.981 | -0.019 | 0.350 | 0.505 |

Pairwise DeLong tests comparing ROC curves for plasma biomarker classification of Thal amyloid phase categories. Comparisons are shown for the two binary contrasts used in the ROC analyses: Thal amyloid phase A0-A1 versus A2-A3 and A0 versus A3. AUC1 and AUC2 correspond to the AUC estimates for biomarker 1 and biomarker 2, respectively. Delta AUC is calculated as AUC1 minus AUC2, such that positive values indicate a higher AUC for biomarker 1. Raw p-values are from paired DeLong tests. FDR p-values were calculated using the Benjamini-Hochberg method within each binary contrast. Bold FDR p-values indicate p < 0.05. For comparability, analyses were performed in the complete-case subset with non-missing values for the six plasma biomarker measures (n=269). AUC = area under the curve; FDR = false discovery rate; ROC = receiver operating characteristic.

**Supplementary Table 21.** Pairwise DeLong comparisons of plasma biomarker AUCs for classification of Braak stage categories.

| **Biomarker 1** | **AUC_1_** | **Biomarker 2** | **AUC_2_** | **ΔAUC (1−2)** | **Raw p** | **p (FDR)** |
| --- | --- | --- | --- | --- | --- | --- |
| **Braak: I-II vs III-VI** | | | | | | |
| P-tau217 | 0.720 | P-tau217/Aβ42 | 0.696 | 0.024 | 0.463 | 0.951 |
| P-tau217 | 0.720 | BD P-tau217 | 0.722 | -0.002 | 0.964 | 0.990 |
| P-tau217 | 0.720 | BD P-tau217/Aβ42 | 0.699 | 0.021 | 0.745 | 0.990 |
| P-tau217 | 0.720 | BD P-tau217/P-tau217 | 0.635 | 0.084 | 0.466 | 0.951 |
| P-tau217 | 0.720 | BD P-tau217/BD tau | 0.721 | -0.001 | 0.990 | 0.990 |
| P-tau217/Aβ42 | 0.696 | BD P-tau217 | 0.722 | -0.026 | 0.570 | 0.951 |
| P-tau217/Aβ42 | 0.696 | BD P-tau217/Aβ42 | 0.699 | -0.003 | 0.955 | 0.990 |
| P-tau217/Aβ42 | 0.696 | BD P-tau217/P-tau217 | 0.635 | 0.061 | 0.561 | 0.951 |
| P-tau217/Aβ42 | 0.696 | BD P-tau217/BD tau | 0.721 | -0.025 | 0.700 | 0.990 |
| BD P-tau217 | 0.722 | BD P-tau217/Aβ42 | 0.699 | 0.024 | 0.255 | 0.951 |
| BD P-tau217 | 0.722 | BD P-tau217/P-tau217 | 0.635 | 0.087 | 0.217 | 0.951 |
| BD P-tau217 | 0.722 | BD P-tau217/BD tau | 0.721 | 0.002 | 0.958 | 0.990 |
| BD P-tau217/Aβ42 | 0.699 | BD P-tau217/P-tau217 | 0.635 | 0.063 | 0.335 | 0.951 |
| BD P-tau217/Aβ42 | 0.699 | BD P-tau217/BD tau | 0.721 | -0.022 | 0.403 | 0.951 |
| BD P-tau217/P-tau217 | 0.635 | BD P-tau217/BD tau | 0.721 | -0.085 | 0.131 | 0.951 |
| **Braak: I-II vs V-VI** | | | | | | |
| P-tau217 | 0.904 | P-tau217/Aβ42 | 0.908 | -0.003 | 0.892 | 0.949 |
| P-tau217 | 0.904 | BD P-tau217 | 0.943 | -0.038 | 0.261 | 0.652 |
| P-tau217 | 0.904 | BD P-tau217/Aβ42 | 0.927 | -0.023 | 0.527 | 0.741 |
| P-tau217 | 0.904 | BD P-tau217/P-tau217 | 0.879 | 0.025 | 0.697 | 0.805 |
| P-tau217 | 0.904 | BD P-tau217/BD tau | 0.929 | -0.024 | 0.555 | 0.741 |
| P-tau217/Aβ42 | 0.908 | BD P-tau217 | 0.943 | -0.035 | 0.141 | 0.620 |
| P-tau217/Aβ42 | 0.908 | BD P-tau217/Aβ42 | 0.927 | -0.020 | 0.365 | 0.741 |
| P-tau217/Aβ42 | 0.908 | BD P-tau217/P-tau217 | 0.879 | 0.029 | 0.592 | 0.741 |
| P-tau217/Aβ42 | 0.908 | BD P-tau217/BD tau | 0.929 | -0.021 | 0.464 | 0.741 |
| BD P-tau217 | 0.943 | BD P-tau217/Aβ42 | 0.927 | 0.015 | 0.185 | 0.620 |
| BD P-tau217 | 0.943 | BD P-tau217/P-tau217 | 0.879 | 0.064 | 0.100 | 0.620 |
| BD P-tau217 | 0.943 | BD P-tau217/BD tau | 0.929 | 0.014 | 0.414 | 0.741 |
| BD P-tau217/Aβ42 | 0.927 | BD P-tau217/P-tau217 | 0.879 | 0.048 | 0.207 | 0.620 |
| BD P-tau217/Aβ42 | 0.927 | BD P-tau217/BD tau | 0.929 | -0.001 | 0.949 | 0.949 |
| BD P-tau217/P-tau217 | 0.879 | BD P-tau217/BD tau | 0.929 | -0.049 | 0.119 | 0.620 |

Pairwise DeLong tests comparing ROC curves for plasma biomarker classification of Braak stage categories. Comparisons are shown for the two binary contrasts used in the ROC analyses: Braak stage I-II versus III-VI and I-II versus V-VI. AUC1 and AUC2 correspond to the AUC estimates for biomarker 1 and biomarker 2, respectively. Delta AUC is calculated as AUC1 minus AUC2, such that positive values indicate a higher AUC for biomarker 1. Raw p-values are from paired DeLong tests. FDR p-values were calculated using the Benjamini-Hochberg method within each binary contrast. Bold FDR p-values indicate p < 0.05. For comparability, analyses were performed in the complete-case subset with non-missing values for the six plasma biomarker measures (n=269). AUC = area under the curve; FDR = false discovery rate; ROC = receiver operating characteristic.

**Supplementary Table 22.** Biomarker-time coefficients for longitudinal mixed-effects models of cognitive change.

| **Biomarker** | **N** | **n observations** | **β (time × baseline)** | **SE** | **95% CI low** | **95% CI high** | **df** | **p** |
| --- | --- | --- | --- | --- | --- | --- | --- | --- |
| **CDR-SoB** |  |  |  |  |  |  |  |  |
| P-tau217 | 273 | 606 | -0.413 | 0.111 | -0.631 | -0.195 | 157 | <0.001 |
| P-tau217/Aβ42 | 273 | 606 | -0.472 | 0.108 | -0.684 | -0.260 | 124 | <0.001 |
| BD P-tau217 | 273 | 606 | -0.540 | 0.112 | -0.759 | -0.322 | 162 | <0.001 |
| BD P-tau217/Aβ42 | 273 | 606 | -0.578 | 0.115 | -0.802 | -0.353 | 145 | <0.001 |
| BD P-tau217/P-tau217 | 273 | 606 | -0.487 | 0.095 | -0.674 | -0.300 | 125 | <0.001 |
| BD P-tau217/BD tau | 273 | 606 | -0.549 | 0.101 | -0.747 | -0.352 | 169 | <0.001 |
| **MMSE** |  |  |  |  |  |  |  |  |
| P-tau217 | 209 | 518 | -0.554 | 0.131 | -0.810 | -0.297 | 96 | <0.001 |
| P-tau217/Aβ42 | 209 | 518 | -0.562 | 0.121 | -0.799 | -0.325 | 67 | <0.001 |
| BD P-tau217 | 209 | 518 | -0.674 | 0.133 | -0.935 | -0.413 | 106 | <0.001 |
| BD P-tau217/Aβ42 | 209 | 518 | -0.688 | 0.133 | -0.950 | -0.427 | 80 | <0.001 |
| BD P-tau217/P-tau217 | 209 | 518 | -0.416 | 0.114 | -0.640 | -0.192 | 93 | <0.001 |
| BD P-tau217/BD tau | 209 | 518 | -0.623 | 0.124 | -0.865 | -0.381 | 121 | <0.001 |

The table depicts fixed-effect estimates for the interaction between follow-up time and baseline biomarker concentration in longitudinal mixed-effects models of CDR-SoB and MMSE. The longitudinal complete-case subset was defined separately from the pathology complete-case subset: participants were required to have complete baseline data for all six blood biomarker variables, non-missing age and years of education, and an available cognitive measure at the first visit for the relevant outcome, but neuropathology variables such as NIA-AA ADNC, Braak stage, and Thal phase were not required. N indicates the number of participants included in each model and n observations indicates the number of longitudinal cognitive observations. Biomarkers were standardized before model fitting. CDR-SoB was modeled as -CDR-SoB so that higher values indicate better cognition. Models included follow-up time, baseline biomarker, their interaction, age, years of education, sex, and a participant-level random intercept and random slope for time. CI = confidence interval; SE = standard error.

**Supplementary Table 23.** Model-fit metrics with bootstrapped ΔR² versus the base model from longitudinal mixed-effects models of cognitive change.

| **Biomarker** | **N (obs)** | **N (subjects)** | **R^2^ (base)** | **R^2^ (model)** | **ΔR^2^ (model−base)** | **ΔR^2^ (model−base) 95% CI** | **P-value** | **AIC (model)** | **ΔAIC (model−base)** |
| --- | --- | --- | --- | --- | --- | --- | --- | --- | --- |
| **CDR-SoB** | | | | | | | | | |
| P-tau217 | 606 | 273 | 0.077 | 0.161 | 0.084 | 0.015 to 0.207 | **0.011** | 3017 | -43 |
| P-tau217/Aβ42 | 606 | 273 | 0.077 | 0.203 | 0.126 | 0.041 to 0.256 | **0.010** | 3004 | -55 |
| BD P-tau217 | 606 | 273 | 0.077 | 0.225 | 0.148 | 0.055 to 0.264 | **0.010** | 2982 | -77 |
| BD P-tau217/Aβ42 | 606 | 273 | 0.077 | 0.237 | 0.160 | 0.071 to 0.286 | **0.010** | 2980 | -79 |
| BD P-tau217/P-tau217 | 606 | 273 | 0.077 | 0.255 | 0.178 | 0.092 to 0.269 | **0.011** | 2989 | -70 |
| BD P-tau217/BD tau | 606 | 273 | 0.077 | 0.228 | 0.151 | 0.056 to 0.288 | **0.010** | 2986 | -73 |
| **MMSE** | | | | | | | | | |
| P-tau217 | 518 | 209 | 0.104 | 0.199 | 0.095 | 0.019 to 0.451 | **0.027** | 2877 | -28 |
| P-tau217/Aβ42 | 518 | 209 | 0.104 | 0.236 | 0.132 | 0.035 to 0.488 | **0.027** | 2870 | -35 |
| BD P-tau217 | 518 | 209 | 0.104 | 0.298 | 0.194 | 0.064 to 0.395 | **0.027** | 2846 | -59 |
| BD P-tau217/Aβ42 | 518 | 209 | 0.104 | 0.328 | 0.224 | 0.080 to 0.438 | **0.027** | 2840 | -65 |
| BD P-tau217/P-tau217 | 518 | 209 | 0.104 | 0.284 | 0.180 | 0.073 to 0.301 | **0.027** | 2857 | -49 |
| BD P-tau217/BD tau | 518 | 209 | 0.104 | 0.284 | 0.180 | 0.052 to 0.531 | **0.027** | 2848 | -57 |

This table reports model-fit metrics from longitudinal mixed-effects models evaluating the interaction between follow-up time and baseline biomarker concentration for CDR-SoB and MMSE. The base model included follow-up time, age, years of education, sex, and a participant-level random intercept and random slope for time; biomarker models additionally included baseline biomarker concentration and its interaction with follow-up time. Analyses used the longitudinal complete-case subset, defined separately from the pathology complete-case subset: participants were required to have complete baseline data for all six blood biomarker variables, non-missing age and years of education, and available longitudinal cognitive data for the relevant outcome, but neuropathology variables such as NIA-AA ADNC, Braak stage, and Thal phase were not required. Biomarkers were z-scored within the analysis cell before model fitting. CDR-SoB was modeled as -CDR-SoB so that higher values indicate better cognition. R² was computed from observed versus marginal fixed-effects-only fitted values, applied identically to the base and biomarker models on the same in-sample rows. ΔR² compares each biomarker model with the base model. Bootstrap percentile 95% confidence intervals and Benjamini-Hochberg FDR-adjusted bootstrap p-values were obtained using a cluster bootstrap resampling participants with replacement. ΔAIC is the biomarker-model AIC minus the base-model AIC. ADNC = Alzheimer disease neuropathologic change; AIC = Akaike information criterion; CDR-SoB = Clinical Dementia Rating Sum of Boxes; CI = confidence interval; FDR = false discovery rate; MMSE = Mini-Mental State Examination.

**Supplementary Table 24.** Bootstrapped pairwise comparison of the standardized time × baseline biomarker interaction (β) and marginal model R² between plasma biomarkers in longitudinal mixed-effects models of CDR-SoB.

|  |  | **Biomarker β-estimates** | | | | | **R²** | | | | |
| --- | --- | --- | --- | --- | --- | --- | --- | --- | --- | --- | --- |
| **Biomarker 1** | **Biomarker 2** | **β_1_** | **β_2_** | **Δβ (1−2)** | **Δβ 95% CI** | **Δβ p (FDR)** | **R^2^_1_** | **R^2^_2_** | **ΔR^2^ (1−2)** | **ΔR^2^ 95% CI** | **ΔR^2^ p (FDR)** |
| P-tau217 | P-tau217/Aβ42 | -0.413 | -0.472 | 0.059 | -0.033 to 0.163 | 0.473 | 0.161 | 0.203 | -0.042 | -0.075 to -0.002 | 0.184 |
| P-tau217 | BD P-tau217 | -0.413 | -0.540 | 0.127 | 0.031 to 0.240 | 0.091 | 0.161 | 0.225 | -0.064 | -0.111 to -0.013 | 0.184 |
| P-tau217 | BD P-tau217/Aβ42 | -0.413 | -0.578 | 0.165 | 0.023 to 0.354 | 0.091 | 0.161 | 0.237 | -0.076 | -0.152 to -0.000 | 0.184 |
| P-tau217 | BD P-tau217/P-tau217 | -0.413 | -0.487 | 0.074 | -0.158 to 0.300 | 0.684 | 0.161 | 0.255 | -0.094 | -0.177 to 0.013 | 0.232 |
| P-tau217 | BD P-tau217/BD tau | -0.413 | -0.549 | 0.136 | -0.007 to 0.280 | 0.235 | 0.161 | 0.228 | -0.068 | -0.139 to -0.001 | 0.184 |
| P-tau217/Aβ42 | BD P-tau217 | -0.472 | -0.540 | 0.068 | -0.041 to 0.175 | 0.473 | 0.203 | 0.225 | -0.022 | -0.072 to 0.036 | 0.645 |
| P-tau217/Aβ42 | BD P-tau217/Aβ42 | -0.472 | -0.578 | 0.106 | 0.019 to 0.225 | 0.091 | 0.203 | 0.237 | -0.034 | -0.096 to 0.020 | 0.546 |
| P-tau217/Aβ42 | BD P-tau217/P-tau217 | -0.472 | -0.487 | 0.015 | -0.212 to 0.225 | 0.857 | 0.203 | 0.255 | -0.052 | -0.135 to 0.059 | 0.574 |
| P-tau217/Aβ42 | BD P-tau217/BD tau | -0.472 | -0.549 | 0.077 | -0.062 to 0.196 | 0.473 | 0.203 | 0.228 | -0.026 | -0.091 to 0.036 | 0.620 |
| BD P-tau217 | BD P-tau217/Aβ42 | -0.540 | -0.578 | 0.037 | -0.044 to 0.151 | 0.604 | 0.225 | 0.237 | -0.012 | -0.062 to 0.030 | 0.723 |
| BD P-tau217 | BD P-tau217/P-tau217 | -0.540 | -0.487 | -0.053 | -0.235 to 0.120 | 0.684 | 0.225 | 0.255 | -0.030 | -0.097 to 0.050 | 0.645 |
| BD P-tau217 | BD P-tau217/BD tau | -0.540 | -0.549 | 0.009 | -0.110 to 0.126 | 0.857 | 0.225 | 0.228 | -0.003 | -0.076 to 0.050 | 0.889 |
| BD P-tau217/Aβ42 | BD P-tau217/P-tau217 | -0.578 | -0.487 | -0.090 | -0.325 to 0.090 | 0.604 | 0.237 | 0.255 | -0.018 | -0.084 to 0.067 | 0.766 |
| BD P-tau217/Aβ42 | BD P-tau217/BD tau | -0.578 | -0.549 | -0.028 | -0.202 to 0.087 | 0.833 | 0.237 | 0.228 | 0.009 | -0.065 to 0.073 | 0.872 |
| BD P-tau217/P-tau217 | BD P-tau217/BD tau | -0.487 | -0.549 | 0.062 | -0.083 to 0.198 | 0.604 | 0.255 | 0.228 | 0.027 | -0.067 to 0.100 | 0.723 |

This table reports bootstrapped pairwise comparisons between plasma biomarkers of the standardized fixed-effect interaction coefficient (β) between follow-up time and baseline biomarker concentration, and of the marginal model R², from longitudinal mixed-effects models of CDR-SoB. CDR-SoB was modeled as -CDR-SoB so that higher values indicate better cognition. Analyses used the longitudinal complete-case subset, defined separately from the pathology complete-case subset: participants were required to have complete baseline data for all six blood biomarker variables, non-missing age and years of education, and available CDR-SoB data, but neuropathology variables such as NIA-AA ADNC, Braak stage, and Thal phase were not required. Each model included follow-up time, baseline biomarker concentration, their interaction, age, sex, years of education, and a participant-level random intercept and random slope for time. Biomarkers were z-scored within the analysis cell before model fitting. β and R² are from the full-data models; Δβ and ΔR² are the signed differences between biomarker 1 and biomarker 2. A cluster bootstrap resampling participants with replacement was used; within each replicate, the base model and all six biomarker models were refitted on the same resampled participants, so every pairwise comparison is paired by construction. Bootstrap percentile 95% confidence intervals and Benjamini-Hochberg FDR-adjusted bootstrap p-values are shown. Marginal R² was computed from observed versus marginal fixed-effects-only fitted values. ADNC = Alzheimer disease neuropathologic change; CDR-SoB = Clinical Dementia Rating Sum of Boxes; FDR = false discovery rate.

**Supplementary Table 25.** Bootstrapped pairwise comparison of the standardized time × baseline biomarker interaction (β) and marginal model R² between plasma biomarkers in longitudinal mixed-effects models of MMSE.

|  |  | **Biomarker β-estimates** | | | | | **R²** | | | | |
| --- | --- | --- | --- | --- | --- | --- | --- | --- | --- | --- | --- |
| **Biomarker 1** | **Biomarker 2** | **β_1_** | **β_2_** | **Δβ (1−2)** | **Δβ 95% CI** | **Δβ p (FDR)** | **R^2^_1_** | **R^2^_2_** | **ΔR^2^ (1−2)** | **ΔR^2^ 95% CI** | **ΔR^2^ p (FDR)** |
| P-tau217 | P-tau217/Aβ42 | -0.554 | -0.562 | 0.008 | -0.129 to 0.141 | 0.967 | 0.199 | 0.236 | -0.037 | -0.083 to 0.019 | 0.403 |
| P-tau217 | BD P-tau217 | -0.554 | -0.674 | 0.121 | -0.049 to 0.238 | 0.662 | 0.199 | 0.298 | -0.099 | -0.159 to -0.009 | 0.288 |
| P-tau217 | BD P-tau217/Aβ42 | -0.554 | -0.688 | 0.135 | -0.125 to 0.305 | 0.710 | 0.199 | 0.328 | -0.129 | -0.204 to 0.181 | 0.288 |
| P-tau217 | BD P-tau217/P-tau217 | -0.554 | -0.416 | -0.138 | -0.412 to 0.189 | 0.662 | 0.199 | 0.284 | -0.085 | -0.183 to 0.288 | 0.422 |
| P-tau217 | BD P-tau217/BD tau | -0.554 | -0.623 | 0.069 | -0.133 to 0.309 | 0.662 | 0.199 | 0.284 | -0.085 | -0.371 to 0.256 | 0.291 |
| P-tau217/Aβ42 | BD P-tau217 | -0.562 | -0.674 | 0.113 | -0.088 to 0.235 | 0.662 | 0.236 | 0.298 | -0.062 | -0.134 to 0.248 | 0.291 |
| P-tau217/Aβ42 | BD P-tau217/Aβ42 | -0.562 | -0.688 | 0.127 | -0.047 to 0.222 | 0.662 | 0.236 | 0.328 | -0.092 | -0.149 to 0.004 | 0.288 |
| P-tau217/Aβ42 | BD P-tau217/P-tau217 | -0.562 | -0.416 | -0.146 | -0.385 to 0.135 | 0.662 | 0.236 | 0.284 | -0.048 | -0.150 to 0.333 | 0.542 |
| P-tau217/Aβ42 | BD P-tau217/BD tau | -0.562 | -0.623 | 0.061 | -0.107 to 0.273 | 0.662 | 0.236 | 0.284 | -0.048 | -0.317 to 0.290 | 0.291 |
| BD P-tau217 | BD P-tau217/Aβ42 | -0.674 | -0.688 | 0.014 | -0.127 to 0.139 | 0.966 | 0.298 | 0.328 | -0.030 | -0.076 to 0.023 | 0.458 |
| BD P-tau217 | BD P-tau217/P-tau217 | -0.674 | -0.416 | -0.258 | -0.476 to 0.067 | 0.662 | 0.298 | 0.284 | 0.014 | -0.073 to 0.146 | 0.786 |
| BD P-tau217 | BD P-tau217/BD tau | -0.674 | -0.623 | -0.051 | -0.158 to 0.152 | 0.967 | 0.298 | 0.284 | 0.014 | -0.318 to 0.076 | 0.786 |
| BD P-tau217/Aβ42 | BD P-tau217/P-tau217 | -0.688 | -0.416 | -0.272 | -0.494 to 0.037 | 0.662 | 0.328 | 0.284 | 0.044 | -0.040 to 0.312 | 0.458 |
| BD P-tau217/Aβ42 | BD P-tau217/BD tau | -0.688 | -0.623 | -0.065 | -0.157 to 0.155 | 0.966 | 0.328 | 0.284 | 0.044 | -0.288 to 0.102 | 0.447 |
| BD P-tau217/P-tau217 | BD P-tau217/BD tau | -0.416 | -0.623 | 0.207 | -0.021 to 0.443 | 0.662 | 0.284 | 0.284 | 0.001 | -0.350 to 0.091 | 0.910 |

This table reports bootstrapped pairwise comparisons between plasma biomarkers of the standardized fixed-effect interaction coefficient (β) between follow-up time and baseline biomarker concentration, and of the marginal model R², from longitudinal mixed-effects models of MMSE. Analyses used the longitudinal complete-case subset, defined separately from the pathology complete-case subset: participants were required to have complete baseline data for all six blood biomarker variables, non-missing age and years of education, and available MMSE data, but neuropathology variables such as NIA-AA ADNC, Braak stage, and Thal phase were not required. Each model included follow-up time, baseline biomarker concentration, their interaction, age, sex, years of education, and a participant-level random intercept and random slope for time. Biomarkers were z-scored within the analysis cell before model fitting. β and R² are from the full-data models; Δβ and ΔR² are the signed differences between biomarker 1 and biomarker 2. A cluster bootstrap resampling participants with replacement was used; within each replicate, the base model and all six biomarker models were refitted on the same resampled participants, so every pairwise comparison is paired by construction. Bootstrap percentile 95% confidence intervals and Benjamini-Hochberg FDR-adjusted bootstrap p-values are shown. Marginal R² was computed from observed versus marginal fixed-effects-only fitted values. ADNC = Alzheimer disease neuropathologic change; FDR = false discovery rate; MMSE = Mini-Mental State Examination.

**Supplementary Table 26.** Sensitivity analyses evaluating associations between cerebral amyloid angiopathy and plasma biomarkers across nested adjustment models.

| **Biomarker** | **N** | **Unadjusted** | **p** | **Age + sex + interval** | **p** | **Age + sex + interval + plaques** | **p** | **Age + sex + interval + plaques + tangles** | **p** | **Interpretation** |
| --- | --- | --- | --- | --- | --- | --- | --- | --- | --- | --- |
| P-tau217 | 269 | 0.691 [0.461, 0.921] | <0.001 | 0.686 [0.454, 0.917] | <0.001 | 0.000 [-0.225, 0.226] | 0.999 | -0.099 [-0.313, 0.116] | 0.366 | Association attenuated after plaque adjustment |
| P-tau217/Aβ42 | 269 | 0.785 [0.559, 1.010] | <0.001 | 0.800 [0.573, 1.028] | <0.001 | 0.071 [-0.142, 0.283] | 0.513 | -0.018 [-0.221, 0.185] | 0.862 | Association attenuated after plaque adjustment |
| BD P-tau217 | 269 | 0.858 [0.636, 1.080] | <0.001 | 0.850 [0.626, 1.074] | <0.001 | 0.081 [-0.119, 0.280] | 0.426 | -0.047 [-0.222, 0.127] | 0.592 | Association attenuated after plaque adjustment |
| BD P-tau217/Aβ42 | 269 | 0.919 [0.701, 1.137] | <0.001 | 0.926 [0.705, 1.147] | <0.001 | 0.129 [-0.059, 0.318] | 0.178 | 0.010 [-0.156, 0.175] | 0.909 | Association attenuated after plaque adjustment |
| BD P-tau217/P-tau217 | 269 | 0.778 [0.552, 1.004] | <0.001 | 0.769 [0.541, 0.997] | <0.001 | 0.173 [-0.061, 0.406] | 0.148 | 0.050 [-0.166, 0.265] | 0.651 | Association attenuated after plaque adjustment |
| BD P-tau217/BD tau | 269 | 0.900 [0.681, 1.120] | <0.001 | 0.917 [0.697, 1.137] | <0.001 | 0.127 [-0.062, 0.316] | 0.186 | -0.001 [-0.163, 0.160] | 0.988 | Association attenuated after plaque adjustment |

The table depicts linear regression estimates for the association between cerebral amyloid angiopathy and each plasma biomarker. Biomarkers were log-transformed and standardized before model fitting, so β values represent mean differences in biomarker levels in SD units for co-pathology present versus absent. Analyses used the maximized co-pathology-specific complete-case subset for cerebral amyloid angiopathy. This subset required complete data for all six plasma biomarker variables, age at death, sex, interval between last plasma collection and autopsy, plaque burden, tangle burden, and cerebral amyloid angiopathy status, but did not require complete data for the other co-pathologies. The effective analytic sample size was n=269. Models followed a nested adjustment ladder: unadjusted; adjusted for age, sex, and autopsy-blood interval; additionally adjusted for plaque burden; and additionally adjusted for both plaque and tangle burden. The interpretation column summarizes the nominal p-value pattern across this adjustment ladder using p < 0.05. 'Attenuated' indicates that an initially significant association was no longer nominally significant after the specified adjustment. CI = confidence interval.

**Supplementary Table 27.** Sensitivity analyses evaluating associations between Lewy body pathology and plasma biomarkers across nested adjustment models.

| **Biomarker** | **N** | **Unadjusted** | **p** | **Age + sex + interval** | **p** | **Age + sex + interval + plaques** | **p** | **Age + sex + interval + plaques + tangles** | **p** | **Interpretation** |
| --- | --- | --- | --- | --- | --- | --- | --- | --- | --- | --- |
| P-tau217 | 262 | -0.042 [-0.287, 0.203] | 0.736 | -0.008 [-0.262, 0.246] | 0.951 | -0.052 [-0.249, 0.145] | 0.603 | -0.071 [-0.256, 0.115] | 0.454 | No association |
| P-tau217/Aβ42 | 262 | 0.065 [-0.180, 0.310] | 0.601 | 0.052 [-0.204, 0.307] | 0.691 | 0.004 [-0.182, 0.189] | 0.970 | -0.014 [-0.189, 0.161] | 0.878 | No association |
| BD P-tau217 | 262 | 0.020 [-0.225, 0.265] | 0.875 | 0.053 [-0.202, 0.308] | 0.683 | 0.002 [-0.172, 0.176] | 0.983 | -0.022 [-0.172, 0.128] | 0.772 | No association |
| BD P-tau217/Aβ42 | 262 | 0.095 [-0.150, 0.340] | 0.445 | 0.094 [-0.161, 0.349] | 0.469 | 0.041 [-0.124, 0.205] | 0.627 | 0.018 [-0.124, 0.159] | 0.805 | No association |
| BD P-tau217/P-tau217 | 262 | 0.110 [-0.135, 0.355] | 0.377 | 0.130 [-0.124, 0.384] | 0.315 | 0.087 [-0.112, 0.285] | 0.392 | 0.063 [-0.116, 0.242] | 0.490 | No association |
| BD P-tau217/BD tau | 262 | 0.237 [-0.007, 0.480] | 0.057 | 0.199 [-0.054, 0.452] | 0.123 | 0.146 [-0.018, 0.309] | 0.080 | 0.122 [-0.016, 0.259] | 0.083 | No association |

The table depicts linear regression estimates for the association between Lewy body pathology and each plasma biomarker. Biomarkers were log-transformed and standardized before model fitting, so β values represent mean differences in biomarker levels in SD units for co-pathology present versus absent. Analyses used the maximized co-pathology-specific complete-case subset for Lewy body pathology. This subset required complete data for all six plasma biomarker variables, age at death, sex, interval between last plasma collection and autopsy, plaque burden, tangle burden, and Lewy body pathology status, but did not require complete data for the other co-pathologies. The effective analytic sample size was n=262. Models followed a nested adjustment ladder: unadjusted; adjusted for age, sex, and autopsy-blood interval; additionally adjusted for plaque burden; and additionally adjusted for both plaque and tangle burden. The interpretation column summarizes the nominal p-value pattern across this adjustment ladder using p < 0.05. 'Attenuated' indicates that an initially significant association was no longer nominally significant after the specified adjustment. CI = confidence interval.

**Supplementary Table 28.** Sensitivity analyses evaluating associations between TDP-43 pathology and plasma biomarkers across nested adjustment models.

| **Biomarker** | **N** | **Unadjusted** | **p** | **Age + sex + interval** | **p** | **Age + sex + interval + plaques** | **p** | **Age + sex + interval + plaques + tangles** | **p** | **Interpretation** |
| --- | --- | --- | --- | --- | --- | --- | --- | --- | --- | --- |
| P-tau217 | 255 | 0.364 [0.101, 0.626] | 0.007 | 0.344 [0.077, 0.611] | 0.012 | 0.110 [-0.104, 0.324] | 0.311 | -0.017 [-0.223, 0.188] | 0.868 | Association attenuated after plaque adjustment |
| P-tau217/Aβ42 | 255 | 0.363 [0.101, 0.626] | 0.007 | 0.363 [0.095, 0.632] | 0.008 | 0.108 [-0.095, 0.311] | 0.295 | -0.008 [-0.204, 0.187] | 0.934 | Association attenuated after plaque adjustment |
| BD P-tau217 | 255 | 0.477 [0.218, 0.737] | <0.001 | 0.477 [0.212, 0.742] | <0.001 | 0.209 [0.020, 0.399] | 0.030 | 0.048 [-0.120, 0.216] | 0.574 | Association attenuated after plaque and tangle adjustment |
| BD P-tau217/Aβ42 | 255 | 0.474 [0.214, 0.733] | <0.001 | 0.487 [0.221, 0.753] | <0.001 | 0.205 [0.026, 0.385] | 0.025 | 0.053 [-0.107, 0.213] | 0.514 | Association attenuated after plaque and tangle adjustment |
| BD P-tau217/P-tau217 | 255 | 0.472 [0.213, 0.732] | <0.001 | 0.502 [0.239, 0.765] | <0.001 | 0.284 [0.066, 0.501] | 0.011 | 0.131 [-0.072, 0.334] | 0.205 | Association attenuated after plaque and tangle adjustment |
| BD P-tau217/BD tau | 255 | 0.533 [0.275, 0.791] | <0.001 | 0.536 [0.272, 0.799] | <0.001 | 0.257 [0.078, 0.435] | 0.005 | 0.096 [-0.059, 0.252] | 0.223 | Association attenuated after plaque and tangle adjustment |

The table depicts linear regression estimates for the association between TDP-43 pathology and each plasma biomarker. Biomarkers were log-transformed and standardized before model fitting, so β values represent mean differences in biomarker levels in SD units for co-pathology present versus absent. Analyses used the maximized co-pathology-specific complete-case subset for TDP-43 pathology. This subset required complete data for all six plasma biomarker variables, age at death, sex, interval between last plasma collection and autopsy, plaque burden, tangle burden, and TDP-43 pathology status, but did not require complete data for the other co-pathologies. The effective analytic sample size was n=255. Models followed a nested adjustment ladder: unadjusted; adjusted for age, sex, and autopsy-blood interval; additionally adjusted for plaque burden; and additionally adjusted for both plaque and tangle burden. The interpretation column summarizes the nominal p-value pattern across this adjustment ladder using p < 0.05. 'Attenuated' indicates that an initially significant association was no longer nominally significant after the specified adjustment. CI = confidence interval.

**Supplementary Table 29.** Sensitivity analyses evaluating associations between vascular pathology and plasma biomarkers across nested adjustment models.

| **Biomarker** | **N** | **Unadjusted** | **p** | **Age + sex + interval** | **p** | **Age + sex + interval + plaques** | **p** | **Age + sex + interval + plaques + tangles** | **p** | **Interpretation** |
| --- | --- | --- | --- | --- | --- | --- | --- | --- | --- | --- |
| P-tau217 | 269 | -0.032 [-0.274, 0.210] | 0.795 | -0.092 [-0.341, 0.156] | 0.465 | -0.016 [-0.209, 0.177] | 0.872 | -0.031 [-0.213, 0.151] | 0.737 | No association |
| P-tau217/Aβ42 | 269 | -0.021 [-0.263, 0.221] | 0.866 | -0.044 [-0.294, 0.207] | 0.732 | 0.040 [-0.142, 0.222] | 0.663 | 0.026 [-0.146, 0.199] | 0.762 | No association |
| BD P-tau217 | 269 | -0.021 [-0.262, 0.221] | 0.866 | -0.056 [-0.306, 0.193] | 0.656 | 0.032 [-0.139, 0.203] | 0.711 | 0.012 [-0.135, 0.160] | 0.871 | No association |
| BD P-tau217/Aβ42 | 269 | -0.013 [-0.255, 0.229] | 0.917 | -0.022 [-0.272, 0.228] | 0.864 | 0.071 [-0.090, 0.233] | 0.385 | 0.053 [-0.087, 0.192] | 0.460 | No association |
| BD P-tau217/P-tau217 | 269 | 0.005 [-0.237, 0.246] | 0.970 | 0.021 [-0.228, 0.270] | 0.869 | 0.093 [-0.108, 0.294] | 0.361 | 0.074 [-0.109, 0.256] | 0.427 | No association |
| BD P-tau217/BD tau | 269 | -0.071 [-0.312, 0.171] | 0.566 | -0.081 [-0.330, 0.168] | 0.522 | 0.011 [-0.151, 0.173] | 0.892 | -0.009 [-0.146, 0.128] | 0.896 | No association |

The table depicts linear regression estimates for the association between vascular pathology and each plasma biomarker. Biomarkers were log-transformed and standardized before model fitting, so β values represent mean differences in biomarker levels in SD units for co-pathology present versus absent. Analyses used the maximized co-pathology-specific complete-case subset for vascular pathology. This subset required complete data for all six plasma biomarker variables, age at death, sex, interval between last plasma collection and autopsy, plaque burden, tangle burden, and vascular pathology status, but did not require complete data for the other co-pathologies. The effective analytic sample size was n=269. Models followed a nested adjustment ladder: unadjusted; adjusted for age, sex, and autopsy-blood interval; additionally adjusted for plaque burden; and additionally adjusted for both plaque and tangle burden. The interpretation column summarizes the nominal p-value pattern across this adjustment ladder using p < 0.05. 'Attenuated' indicates that an initially significant association was no longer nominally significant after the specified adjustment. CI = confidence interval.

**Supplementary Figure 1.** Plasma biomarker levels and adjusted fold-changes across Thal amyloid phase categories.

Boxplots with individual datapoints show group-wise distributions of p-tau217 (A), p-tau217/Aβ42 (B), BD p-tau217 (C), BD p-tau217/Aβ42 (D), BD p-tau217/p-tau217 (E), and BD p-tau217/BD tau (F) across Thal amyloid phase categories A0, A1, A2, and A3. For boxplots, each biomarker was analyzed using the maximum available analytic sample for that biomarker, restricted to participants with non-missing Thal phase category and model covariates, with the effective sample size shown in each panel title. P-values are from post-hoc contrasts from linear models adjusted for age at death, sex, and interval between plasma sampling and autopsy, with Sidak adjustment for multiple pairwise comparisons. Forest plots show mean fold-changes and 95% CIs for A1 (G), A2 (H), and A3 (I) compared with A0 as the reference group, adjusted for age at death, sex, and interval between plasma sampling and autopsy. For comparability, fold-changes were estimated in a common complete-case subset with non-missing values for all six biomarkers, Thal phase category, and model covariates, with the effective sample size shown in each forest plot title. Aβ = amyloid-β; BD = brain-derived; CI = confidence interval; p-tau = phosphorylated tau.

**Supplementary Figure 2.** Plasma biomarker levels and adjusted fold-changes across Braak stage categories.

Boxplots with individual datapoints show group-wise distributions of p-tau217 (A), p-tau217/Aβ42 (B), BD p-tau217 (C), BD p-tau217/Aβ42 (D), BD p-tau217/p-tau217 (E), and BD p-tau217/BD tau (F) across Braak stage categories I-II, III-IV, and V-VI. For boxplots, each biomarker was analyzed using the maximum available analytic sample for that biomarker, restricted to participants with non-missing Braak stage and model covariates, with the effective sample size shown in each panel title. P-values are from post-hoc contrasts from linear models adjusted for age at death, sex, and interval between plasma sampling and autopsy, with Sidak adjustment for multiple pairwise comparisons. Forest plots show mean fold-changes and 95% CIs for III-IV (G) and V-VI (H) compared with I-II as the reference group, adjusted for age at death, sex, and interval between plasma sampling and autopsy. For comparability, fold-changes were estimated in a common complete-case subset with non-missing values for all six biomarkers, Braak stage, and model covariates, with the effective sample size shown in each forest plot title. Aβ = amyloid-β; BD = brain-derived; CI = confidence interval; p-tau = phosphorylated tau.

**Supplementary Figure 3.** Sensitivity analysis of adjusted fold-changes using the maximum available analytic sample for each biomarker.

Forest plots show adjusted mean fold-changes and 95% CIs for the six plasma biomarker measures across neuropathologic categories using maximum available analytic samples for each biomarker. Panels show fold-changes for Low (A), Intermediate (B), and High (C) NIA-AA AD neuropathologic change compared with Not AD; Thal A1 (D), A2 (E), and A3 (F) compared with A0; and Braak III-IV (G) and V-VI (H) compared with Braak I-II. Models were fit separately for each biomarker and pathology outcome and adjusted for age at death, sex, and interval between plasma sampling and autopsy. This sensitivity analysis did not require complete data across all six biomarkers. Instead, each model used the maximum available analytic sample with non-missing data for the specific biomarker, pathology outcome, and model covariates. The effective sample size for each biomarker-pathology model is shown next to the corresponding biomarker label. AD = Alzheimer's disease; CI = confidence interval; NIA-AA = National Institute on Aging-Alzheimer's Association.

**Supplementary Figure 4.** Continuous associations between plasma biomarkers and neuropathological measures of plaques and tangles using the maximum available analytic sample for each biomarker.

Scatterplots show associations of plasma biomarkers with semiquantitative plaque and tangle measures at autopsy. Panels include p-tau217, p-tau217/Aβ42, BD p-tau217, BD p-tau217/Aβ42, BD p-tau217/p-tau217, and BD p-tau217/BD tau. Panels A-F represent biomarker-plaque associations for the unstratified whole population; G-L for the pathology-stratified Non-AD (Not AD or Low ADNC) and AD (Intermediate and High ADNC) populations. Panels M-R represent biomarker-tangle associations for the unstratified whole population; S-X for the pathology-stratified Non-AD and AD populations. Model annotations show R² and p-values for the smooth term from pathology-biomarker models adjusted for age at death, sex, and interval between plasma sampling and autopsy. Curves represent generalized additive model smooths. This sensitivity analysis did not require complete data across all six blood biomarker variables. Instead, each panel used the maximum available analytic sample with non-missing data for the specific biomarker, pathology outcome, and model covariates. The effective sample size for each model is shown within the corresponding panel. AD = Alzheimer's disease; ADNC = Alzheimer's disease neuropathologic change; BD = brain-derived.

**Supplementary Figure 5.** Linear associations of plasma biomarkers with amyloid plaque burden.

Forest plots show adjusted standardized β coefficients and 95% CIs for the associations between plasma biomarker measures and total amyloid plaque score. Estimates represent the difference in total amyloid plaque score per 1-SD higher biomarker concentration, estimated from linear models adjusted for age at death, sex, and interval between plasma sampling and autopsy. Panels show associations in the unstratified whole study populations, and after stratification into Non-AD and AD groups based on ADNC categories (Non-AD: Not AD and Low ADNC; AD: Intermediate and High ADNC). Analyses were performed in the complete-case subset with non-missing values for all evaluated plasma biomarkers, model covariates, and total amyloid plaque score, with the effective sample size indicated in each panel. ADNC = Alzheimer's disease neuropathologic change.

**Supplementary Figure 6.** Linear associations of plasma biomarkers with neurofibrillary tangle burden.

Forest plots show adjusted standardized β coefficients and 95% CIs for the associations between plasma biomarker measures and total neurofibrillary tangle score. Estimates represent the difference in total neurofibrillary tangle score per 1-SD higher biomarker concentration, estimated from linear models adjusted for age at death, sex, and interval between plasma sampling and autopsy. Panels show associations in the unstratified whole study populations, and after stratification into Non-AD and AD groups based on ADNC categories (Non-AD: Not AD and Low ADNC; AD: Intermediate and High ADNC). Analyses were performed in the complete-case subset with non-missing values for all evaluated plasma biomarkers, model covariates, and total neurofibrillary tangle score, with the effective sample size indicated in each panel. ADNC = Alzheimer's disease neuropathologic change.

**Supplementary Figure 7.** Associations of plasma biomarker measures with neurofibrillary tangle burden in the tau-PET subset.

Scatterplots show associations between plasma biomarker measures and total neurofibrillary tangle score in participants with available tau-PET data. Panels show P-tau217 (A), P-tau217/Aβ42 (B), BD P-tau217/Aβ42 (C), BD P-tau217/P-tau217 (D), and BD P-tau217/BD tau (E). Lines represent fitted linear trends and shaded regions represent 95% confidence intervals. Spearman correlation coefficients and p-values are shown within each panel. Analyses were performed in the tau-PET subset (n=15), restricted to participants with available total tangle score, and the plasma biomarker measure shown in the panel. Aβ = amyloid-β; BD = brain-derived; PET = positron emission tomography.

**Supplementary Figure 8.** Associations between tau-PET uptake and plasma biomarker measures.

Scatterplots show associations between tau-PET uptake and plasma biomarker measures. Rows correspond to tau-PET measures: average cortical FTP SUVr, inferior temporal FTP SUVr, and middle temporal FTP SUVr. Columns correspond to plasma biomarker measures: P-tau217, P-tau217/Aβ42, BD P-tau217, BD P-tau217/Aβ42, BD P-tau217/P-tau217, and BD P-tau217/BD tau. Lines represent fitted linear trends and shaded regions represent 95% confidence intervals. Spearman correlation coefficients and p-values are shown within each panel. Analyses were performed in the tau-PET subset (n=15), restricted to participants with available tau-PET measures and the plasma biomarker measures shown. Aβ = amyloid-β; BD = brain-derived; FTP = flortaucipir; PET = positron emission tomography; SUVr = standardized uptake value ratio.

**Supplementary Figure 9.** Sensitivity analyses of plasma biomarker associations with non-AD co-pathologies across nested adjustment models.

Forest plots show linear regression estimates and 95% CIs for the association between each co-pathology and each plasma biomarker. Biomarkers were log-transformed and standardized before model fitting, so estimates represent mean differences in biomarker levels in SD units for co-pathology present versus absent. Co-pathologies include cerebral amyloid angiopathy, Lewy body pathology, TDP-43 pathology, and vascular pathology. Models followed a nested adjustment ladder: unadjusted; adjusted for age at death, sex, and interval between last plasma collection and autopsy; additionally adjusted for plaque burden; and additionally adjusted for both plaque and tangle burden. Each co-pathology used its own maximized complete-case subset, requiring complete data for all six plasma biomarker variables, age at death, sex, autopsy-blood interval, plaque burden, tangle burden, and the co-pathology being analyzed, but not requiring complete data for the other co-pathologies. The effective analytic sample size is shown in each panel title. AD = Alzheimer’s disease; CAA = cerebral amyloid angiopathy; CI = confidence interval; SD = standard deviation; TDP-43 = transactive response DNA-binding protein 43.
